# TCF25 serves as a nutrient sensor to orchestrate metabolic adaptation and cell death by enhancing lysosomal acidification under glucose starvation

**DOI:** 10.1016/j.celrep.2025.116186

**Published:** 2025-08-21

**Authors:** Wenqing Ren, Hui Jiang, Qianqian Song, Yiliang Chen, Chenxiao Tang, Fang Wang, Jing Zhu, Jingming Ren, Yaxing Zhao, Yuan He, Jin Cai, Tianle Zhang, Zhuhong Wang, Chenjie Zhu, Wen Xue, Ai Peng, Xiaona Feng, Yue Liu, Jianqiang Yu, Zheng-gang Liu, Zhenyu Cai

**Affiliations:** 1 Tongji University Cancer Center, Shanghai Tenth People’s Hospital, School of Medicine, Tongji University, Shanghai 200072, China; 2 Department of Biochemistry and Molecular Biology, School of Medicine, Tongji University, Shanghai 200331, China; 3 Center for Nephrology & Metabolomics, Division of Nephrology, Shanghai Tenth People’s Hospital, School of Medicine, Tongji University, Shanghai 200072, China; 4 College of Pharmacy, Ningxia Medical University, Yinchuan, Ningxia Hui Autonomous Region, Yinchuan 750004, China; 5 Laboratory of Immune Cell Biology, Center for Cancer Research, National Cancer Institute, National Institutes of Health, Bethesda, MD 20892, USA; 6 State Key Laboratory of Cardiology and Medical Innovation Center, Shanghai East Hospital, School of Medicine, Tongji University, Shanghai 200120, China; 7 These authors contributed equally; 8 Lead contact

## Abstract

Cells adapt to nutrient limitation by activating catabolic and inhibiting anabolic pathways, yet prolonged stress may lead to cell death. How cells orchestrate metabolic adaptation and cell death to nutrient stress is poorly understood. We conduct a genome-wide CRISPR-Cas9 screen to identify regulators in glucose-starvation-induced cell death and find a group of genes in lysosomal pathway is enriched following glucose starvation. We focus on one candidate gene, Transcriptional Factor 25 (TCF25). We find TCF25 enhances lysosomal acidification by targeting V-ATPase, promoting autophagy and ATP generation under glucose starvation. However, prolonged glucose starvation constitutively activates ferritinophagy via TCF25, increasing lysosomal membrane permeability (LMP) and leading to lysosome-dependent cell death (LDCD). Knocking out TCF25 or V-ATPase components prevents cell death. Furthermore, TCF25 deficiency protects mice from hepatic ischemia-reperfusion injury. Our findings identify TCF25 as a crucial nutrient sensor that regulates lysosomal activity, offering potential therapeutic targets for metabolic and ischemic disorders.

## In brief

Ren et al. demonstrate that TCF25 enhances lysosomal acidification via V-ATPase during glucose starvation, promoting autophagy to maintain energy balance. However, prolonged starvation makes TCF25-mediated lysosomal-autophagic activity crucial for ferritinophagy, ultimately causing lysosome-dependent cell death.

## Graphical Abstract

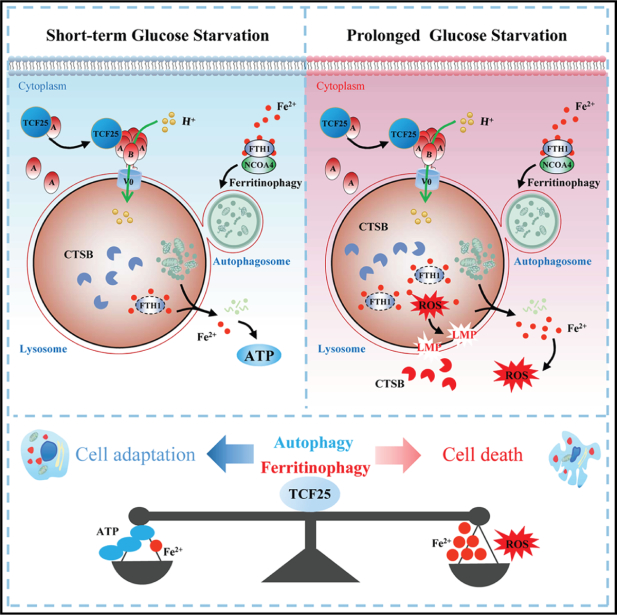

## INTRODUCTION

Glucose is the major carbon source to generate energy (in the form of ATP) and provide the building blocks for growing cells.^1^ When glucose is scarce, cells initiate several responses to adapt to the low glucose environment. One of the key responses is the activation of AMP-activated protein kinase (AMPK), which is a key sensor of cellular energy status.^2,3^ Activated AMPK promotes energy conservation and production by increasing catabolic and decreasing anabolic pathways under glucose starvation.^2,3^ Cells can also undergo autophagy in response to glucose deprivation,^4^ which is a lysosome-dependent cellular recycling process to generate energy and maintain cellular function under stress conditions. However, sustained or severe glucose starvation may cause cell death, leading to tissue injury and pathogenesis of various diseases.^5,6^ Although various regulators involved in metabolic reprogramming under glucose starvation have been extensively investigated, how cells orchestrate metabolic adaptation and cell death during glucose starvation is not well understood.

As an essential catabolic organelle, the lysosome plays multiple roles in cellular adaptation to glucose starvation, including facilitating autophagy, mediating glycogen breakdown and protein degradation, as well as regulating energy-sensing AMPK/mammalian target of rapamycin (mTOR) signaling pathways.^7–9^ By orchestrating these processes, lysosomes help cells cope with nutrient stress and maintain energy balance during periods of glucose starvation. However, it has been shown that lysosome dysfunction may cause lysosomal membrane permeability (LMP) to trigger lysosome-dependent cell death (LDCD) in ischemia-reperfusion injury (IRI).^10^ Therefore, the structural integrity and functional balance of lysosomes need be precisely maintained to ensure cell survival under low-glucose conditions.

Lysosomal activity is mainly regulated by its luminal acidification, as dozens of lysosomal hydrolytic enzymes need to degrade macromolecules under low pH.^11^ Lysosome pH gradients are maintained by the vacuolar (H^+^) ATPase (or V-ATPase), which is a large multi-subunit complex that pumps protons from the cytosol into the lysosomal lumen by consuming ATP.^12^ The V-ATPase is organized into two distinct sectors: a cytosolic ATP-hydrolyzing V1 domain and a membrane-embedded V0 proton channel, which function together by coupling the energy of ATP hydrolysis to the transport of protons across the lipid bilayer.^13^ Changes in V-ATPase activity can modulate AMPK/mTOR activation,^14^ autophagic flux,^15^ and lysosomal degradative activity,^12^ which are required for nutrient sensing and metabolic homeostasis. A previous study showed that glucose starvation increased V-ATPase assembly and lysosomal acidification in mammalian cells, which may contribute to energy production under low-glucose conditions.^16^ However, the mechanisms by which glucose starvation increases lysosomal acidification are not well understood.

In this study, we initially perform a genome-wide CRISPR-Cas9 screen to identify potential regulators in glucose-starvation-induced cell death. We find a set of single-guide RNA (sgRNA)-targeting genes involved in lysosome pathway is significantly enriched after glucose starvation. We further focus on one candidate gene named Transcriptional Factor 25 (TCF25) and show that its encoded protein localizes to lysosome and enhances lysosomal acidification by targeting V-ATPase in response to glucose starvation. This process is required for energy maintenance by promoting catabolism under low-glucose conditions. However, sustained glucose starvation constitutively activates TCF25-mediated ferritinophagy and generates redox-active iron to eventually trigger LDCD. These findings suggest that TCF25-regulated lysosomal activity represents a previously unrecognized metabolic checkpoint to dictate cell fate under glucose starvation, which may provide potential therapeutic targets for the treatment of metabolic and ischemic disorders.

## RESULTS

### A genome-wide CRISPR-Cas9 screen identifies TCF25 as a key mediator in glucose-starvation-induced cell death

**Figure 1.**
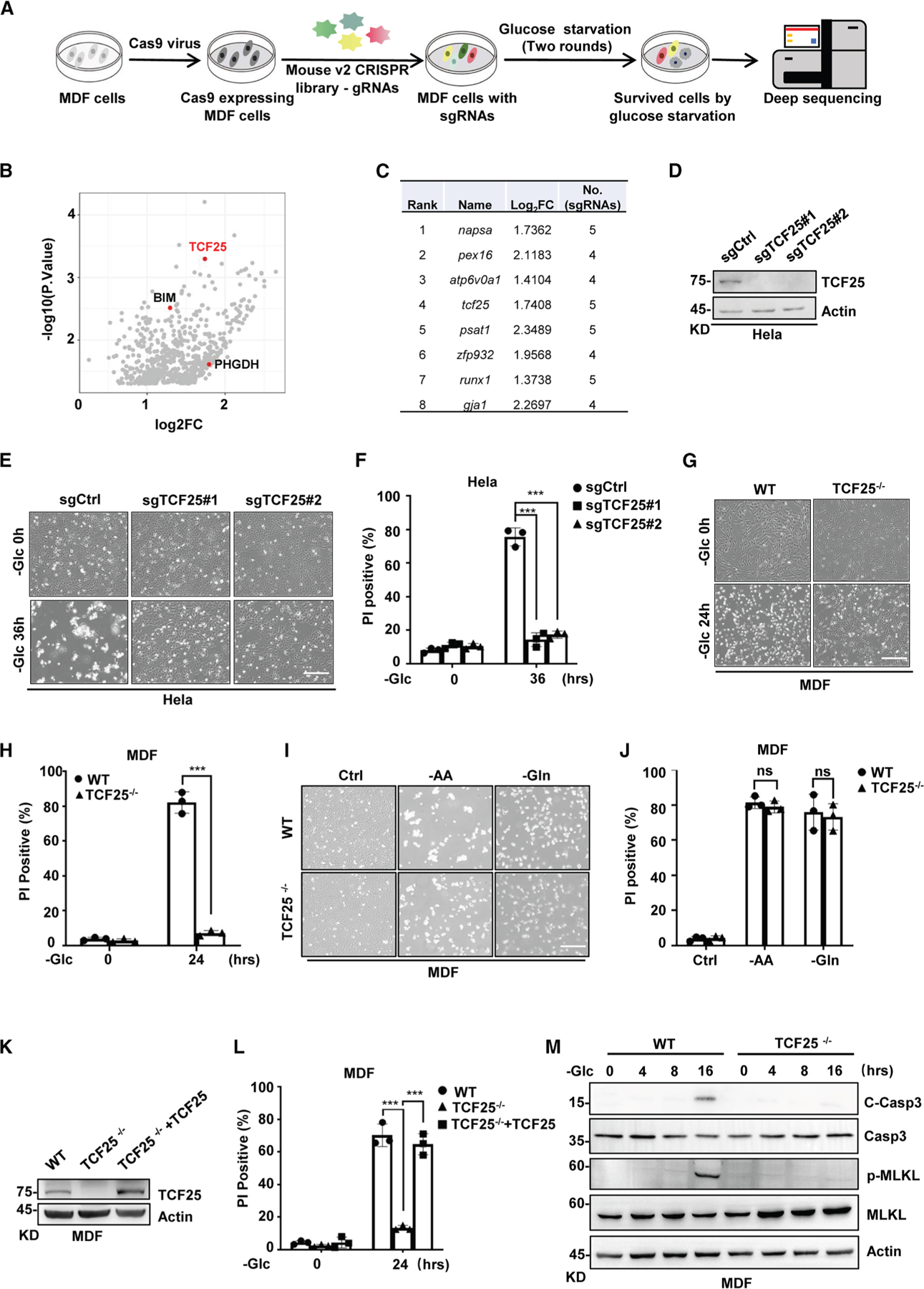
A genome-wide CRISPR screen identifies TCF25 as a key mediator in glucose-starvation-induced cell death (A) CRISPR-Cas9 screening workflow to identify regulators in glucose-starvation-induced cell death. (B) A scatterplot showing the distribution of enriched sgRNAs in MDFs starved without glucose for 24 h. The sgRNAs targeting TCF25 and known genes that contribute to glucose-starvation-induce cell death are highlighted. (C) List of top-ranked sgRNA hits from the CRISPR screen. The fold change (Log_2_FC) and the number of individual sgRNAs are shown. (D) HeLa cells were stably transfected with sgRNA-Control (sgCtrl) and two individual sgRNAs targeting TCF25 (sgTCF25#1 and sgTCF25#2), respectively. TCF25 expression was examined by immunoblotting with its specific antibody. (E) sgCtrl, sgTCF25#1, and sgTCF25#2 HeLa cells were starved without glucose for 36 h and the representative images are shown. Scale bar, 100 μm. (F) Cell death of the HeLa cells in (E) was determined by PI staining. (G) WT and TCF25^−/−^ MDFs were starved without glucose for 24 h and the representative images are shown. Scale bar, 100 μm. (H) Cell death of the MDFs in (G) was determined by PI staining. (I) WT and TCF25^−/−^ MDFs were starved without amino acids (−AA) for 24 h or glutamine (−Gln) for 48 h and the representative images are shown. Scale bar, 100 μm. (J) Cell death of the MDFs in (I) was determined by PI staining. (K) TCF25 expression was examined in WT, TCF25^−/−^, and TCF25 reconstituted MDFs by immunoblotting with its specific antibody. (L) WT, TCF25^−/−^, and TCF25 reconstituted MDFs were starved without glucose for 24 h and cell death was determined by PI staining. (M) WT and TCF25^−/−^ MDFs were starved without glucose at the indicated time points. Cells were lysed and immunoblotted with the indicated antibodies. All western data are representative of three independent experiments. Bar graphs represent the mean ± SD from three independent experiments. Statistical analysis was performed using a two-sided student’s t test. The levels of significance are indicated by ***p < 0.001; ns, not significant (two-way ANOVA).

To identify genes that are involved in glucose-starvation-induced cell death, we transfected immortalized mouse dermal fibroblasts (MDFs) with Lenti-CRISPR library (Addgene #67988) targeting 18,424 mouse genes and then starved these cells without glucose for 24 h to induce cell death. After treatment with two rounds of glucose-deprived medium, surviving cells were collected to determine sgRNA representation by deep sequencing (Figure 1A). The enriched candidate genes were picked on the basis of the following criteria: (1) normalized sgRNA count was not less than 20 and (2) at least three out of five different sgRNAs were enriched for the candidate gene (Figure 1B; Table S1). Several known regulators that contribute to glucose-starvation-induced cell death were identified by our screening, such as phosphohydroxyglutamate dehydrogenase (PHGDH)^17^ and BCL2-like 11 (BIM)^18,19^ (Figure 1B), indicating the validity of our experimental screen. Among these candidate genes, a gene named TCF25 was of particular interest to us, because it was among the top 10 candidate genes ranked by robust rank aggregation (RRA) score^20^ in our screening and its biological function in metabolic stress is unknown (Figure 1C). TCF25, also named as nuclear-localized protein 1 (Nulp1), was initially reported as a member of the basic-helix-loop-helix (bHLH) family of transcription factors and has been shown to alleviate cardiac hypertrophy by acting as a transcriptional repressor.^21–23^ Since TCF25 is a highly conserved gene during evolution,^22^ we first tested its requirement for glucose-starvation-induced cell death in other human cell lines. By generating TCF25-deficient human HeLa and HT-29 cells, we found TCF25 knockout protected these cells from glucose-starvation-induced cell death (Figures 1D–1F and S1A–S1C), suggesting TCF25 is most likely involved in glucose-starvation-induced cell death.

To further investigate the role of TCF25 in glucose-starvation-induced cell death, we generated TCF25 knockout mice using the CRISPR-Cas9 system (Figures S2A–S2C). Consistent with a previous study,^23^ TCF25 deficiency did not cause any morphological difference at the basal level (Figures S2D and S2E). We then isolated primary mouse dermal fibroblasts (MDFs), mouse lung fibroblasts (MLFs), bone marrow-derived macrophages (BMDMs), and adipocytes from TCF25-deficient mice to investigate TCF25 function in glucose-starvation-induced cell death more broadly. In any of the examined cell types, we found loss of TCF25 significantly protected cells from glucose-starvation-induced cell death (Figures 1G, 1H, and S2F–S2N). Additionally, TCF25 knockout also protected MDFs from glucose transporter 1 inhibitor BAY-876-induced cell death (Figures S1D and S1E). Furthermore, we found TCF25 knockout had no effect on other types of metabolic-stress-induced cell death, such as amino acid- or glutamine-deprivation-induced cell death (Figures 1I and 1J). To preclude any off-target effects in TCF25^−/−^ MDFs, TCF25 expression was reconstituted in these cells (Figure 1K) and glucose-starvation-induced cell death was re-established (Figure 1L). Thus, these data demonstrate that TCF25 is a key mediator in glucose-starvation-induced cell death.

Glucose starvation can induce either apoptosis or necrotic-like cell death such as necroptosis.^24,25^ By examining cleavage of caspase-3 (Casp-3) for apoptosis and phosphorylation of MLKL for necroptosis, we found glucose starvation induced both apoptosis and necroptosis in MDFs, while TCF25 knockout blocked these two types of PCD (Figure 1M). These results suggest that TCF25 acts as an upstream regulator of apoptosis and necroptosis in response to glucose starvation. However, TCF25 knockout had no effect on tumor necrosis factor (TNF)-α-induced apoptosis or necroptosis (Figures S1F and S1G), indicating TCF25 is specific for glucose-starvation-induced cell death. Therefore, these data collectively indicate that TCF25 plays a previously uncharacterized role in metabolic stress by mediating glucose-starvation-induced cell death.

### TCF25 localizes to lysosomes and enhances lysosomal acidification in response to glucose starvation

**Figure 2.**
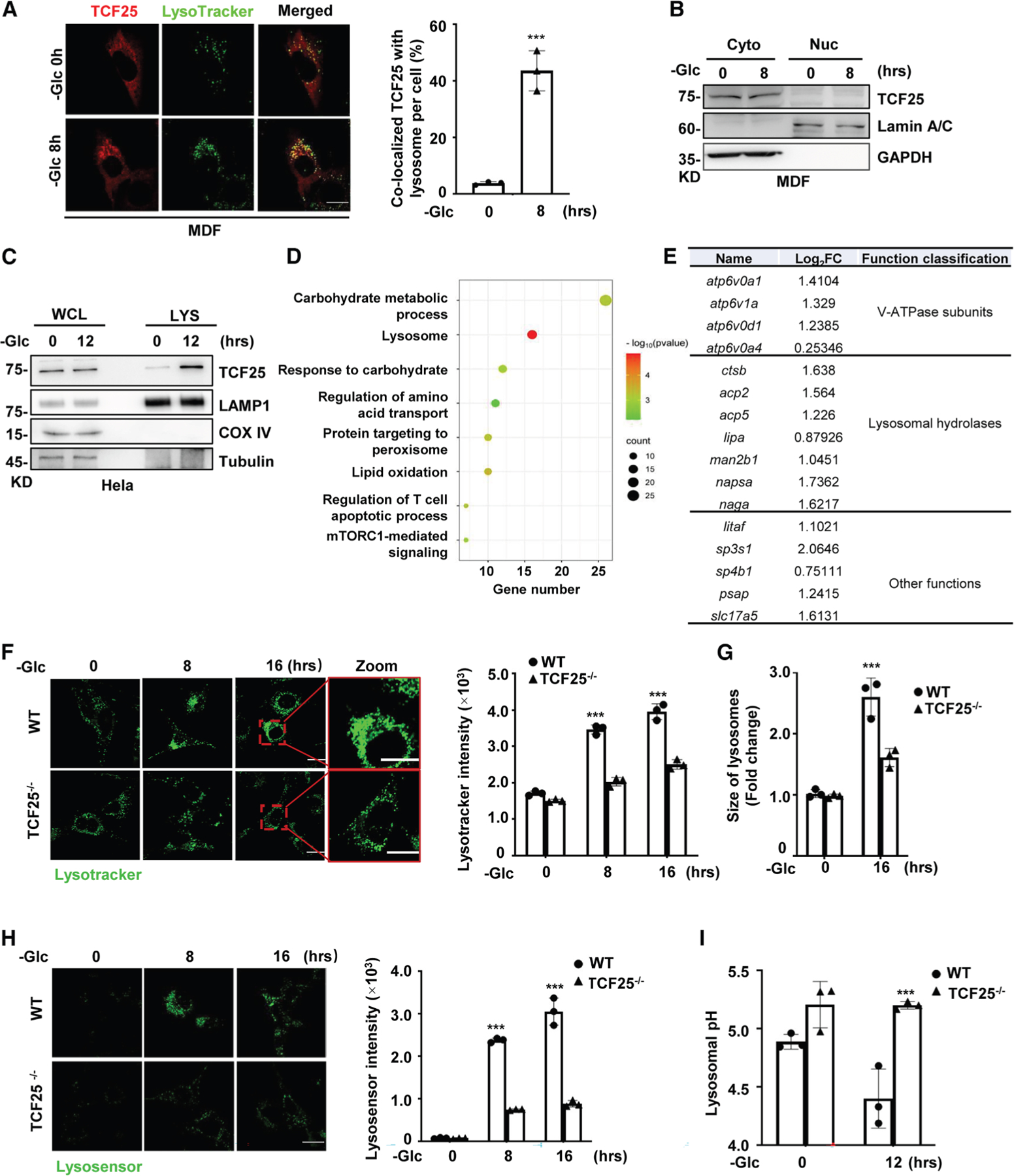
TCF25 localizes to lysosomes and enhances lysosomal acidification in response to glucose starvation (A) MDFs stably expressing mCherry-TCF25 were starved without glucose at the indicated time points and then stained with LysoTracker Green. Left, representative confocal images of the cells are shown. Right, statistical analysis of the co-localized mCherry-TCF25 with LysoTracker Green is shown. Scale bar, 20 μm. (B) MDFs were starved without glucose at the indicated time points and then fractionated into cytosolic (Cyto) and nuclear (Nuc) fractions. The fractions were analyzed by immunoblotting with the indicated antibodies. (C) HeLa cells were starved without glucose at the indicated time points and then fractionated into lysosomal fraction by gradient centrifugation. The whole-cell lysates (WCLs) and lysosomal fraction lysates (LYSs) were analyzed by immunoblotting with anti-TCF25, lysosome marker anti-LAMP1, mitochondrial marker anti-COX IV, and cytoplasmic marker anti-tubulin antibodies, respectively. (D) GO analysis of the sgRNA-targeting genes enriched by glucose starvation in MDF cells. (E) List of the enriched sgRNA hits in lysosome pathway. (F) WT and TCF25^−/−^ MDFs were starved without glucose at the indicated time points and then stained with LysoTracker Green. Left, representative confocal images of the cells are shown. Right, statistical analysis of the LysoTracker Green fluorescence intensity is shown. Scale bar, 20 μm. (G) Statistical analysis of the relative lysosome size in (F) is shown. (H) WT and TCF25^−/−^ MDFs were starved without glucose at the indicated time points and then stained with LysoSensor. Left, representative confocal images of the cells are shown. Right, statistical analysis of the LysoSensor fluorescence intensity is shown. Scale bar, 20 μm. (I) WT and TCF25^−/−^ MDFs were starved without glucose at the indicated time points. Lysosomal pH was measured using fluorescein isothiocyanate (FITC) dextran and tetramethylrhodamine (TMR) dextran as described in the STAR Methods. All western data are representative of three independent experiments. Bar graphs represent the mean ± SD from three independent experiments. Statistical analysis was performed using a two-sided student’s t test. The levels of significance are indicated by *p < 0.05; **p < 0.01; ***p < 0.001; n.s., not significant (two-way ANOVA).

TCF25 was initially identified as a nuclear-localized protein that belonged to a member of the basic-helix-loop-helix (bHLH) family of transcription factors.^21,22^ To investigate nuclear events in glucose-starvation-induced cell death, we first examined the subcellular localization of TCF25 by stably expressing mCherry-tagged TCF25 in cells. To our surprise, we found mCherry-TCF25 protein was uniformly diffused in the cytosol of living cells, while it formed cytosolic puncta under glucose starvation (Figures 2A and S3A). The cytosolic localization of TCF25 was further verified by examining the endogenous TCF25 protein through nuclear and cytosolic fractionation (Figures 2B and S3B). Thus, in contrast to previous reports,^21,22^ our results indicate that TCF25 is mainly expressed in cytoplasm. Interestingly, the puncta of mCherry-TCF25 exhibited extensive co-localization with the lysosome marker LysoTracker (Figures 2A and S3A), and later endosome marker RAB7 (Figure S3C), but no co-localization with the mitochondrial marker MitoTracker (Figure S3D). Furthermore, we isolated the lysosomal fraction from HeLa cells by ultracentrifugation and found the endogenous TCF25 protein was significantly enriched in the lysosomal fraction under glucose starvation (Figure 2C). Additionally, we performed soluble and membrane-bound fractionation analysis and found TCF25 was enriched in the membrane-bound fraction under glucose starvation (Figure S3E), suggesting TCF25 associates with lysosome membrane under glucose starvation. Together, these data indicate that TCF25 is predominantly expressed in cytoplasm but redistributes to lysosome during glucose starvation.

The translocation of TCF25 to lysosomes prompted us to investigate the effects of TCF25 on lysosome status under glucose starvation. Intriguingly, Gene Ontology (GO) enrichment analysis revealed that a set of enriched sgRNA-targeting genes is related to the lysosome pathway (Figure 2D). As shown in Figure 2E, we found these enriched genes in lysosome can be classified into three groups: genes encoding the subunits of V-ATPase complex such as *atp6v1a* and *atp6v0d1*, genes encoding lysosomal hydrolases such as *ctsb* (*cathepsin B*) and *acp2/5* (*acid phosphatase 2/5*), and genes with other functions. We therefore first used LysoTracker Green dye to investigate the status of lysosomes during glucose starvation. Notably, the fluorescence intensity of LysoTracker staining was increased time dependently in wild-type (WT) MDFs during glucose starvation, while such fluorescence intensity only marginally increased in TCF25^−/−^ MDFs (Figure 2F). Furthermore, we found the lysosomes were enlarged in WT MDFs compared to those in TCF25^−/−^ MDFs under prolonged glucose starvation (Figure 2G), suggesting the lysosomes may be dysfunctional. Since the amount of fluorescence obtained by LysoTracker staining arises from the volume of the acidic compartment,^26^ we then examined the lysosomal acidification by using LysoSensor DND-189 probes. The fluorescence intensity of this dye is inversely correlated with the pH in lysosomes. As shown in Figure 2H, we found glucose starvation time-dependently increased fluorescence intensity of LysoSensor in WT but not TCF25^−/−^ MDFs. Similar results were also observed in human HeLa cells (Figure S3F). To further confirm these results, we quantitated the lysosomal pH in both WT and TCF25^−/−^ MDFs. We found glucose starvation further decreased lysosomal pH from around 5 to 4.3 in WT MDFs, while the lysosomal pH barely changed in TCF25^−/−^ MDFs under glucose starvation (Figure 2I). Taken together, these data reveal that TCF25 enhances lysosomal acidification under glucose starvation, which may lead to lysosomal dysfunction.

### TCF25 targets V-ATPase to enhance lysosomal acidification in response to glucose starvation

**Figure 3.**
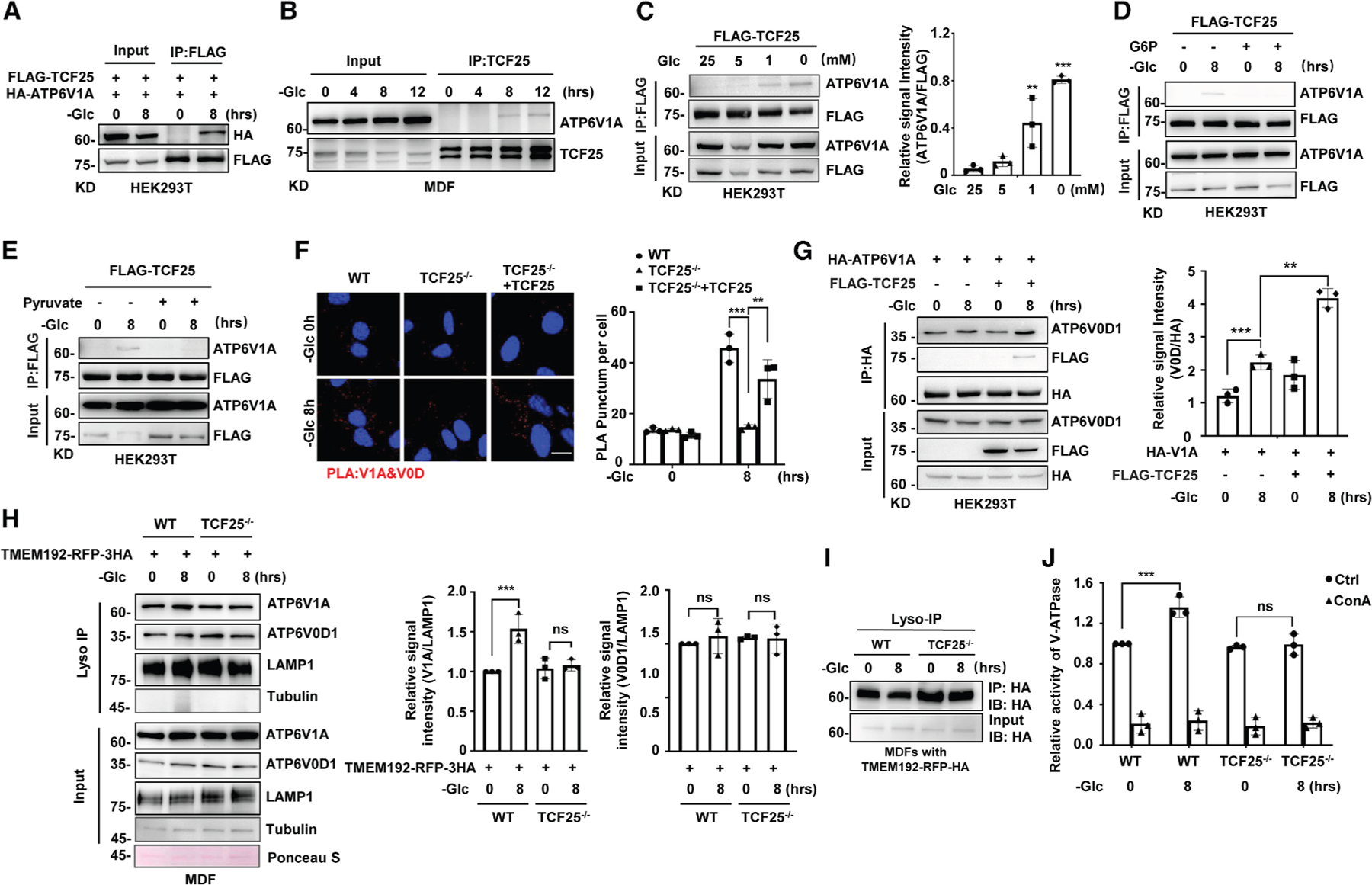
TCF25 enhances lysosomal acidification by targeting V-ATPase in response to glucose starvation (A) HEK293T cells were co-transfected with FLAG-TCF25 and HA-ATP6V1A. The cells were then starved without glucose at the indicated time points. Cell lysates were immunoprecipitated with FLAG antibody (IP:FLAG) and analyzed by immunoblotting with the indicated antibodies. (B) MDFs were starved without glucose at the indicated time points. Cell lysates were immunoprecipitated with TCF25 antibody (IP:TCF25) and analyzed by immunoblotting with the indicated antibodies. (C) HEK293T cells were transfected with FLAG-TCF25 and then treated with different concentrations of glucose for 8 h. Left, cell lysates were immunoprecipitated with FLAG antibody (IP:FLAG) and analyzed by immunoblotting with the indicated antibodies. Right, quantification analysis of co-precipitated ATP6V1A with FLAG-TCF25 as described in the STAR Methods. (D and E) (D) HEK293T cells were transfected with FLAG-TCF25 and then starved without glucose in the presence of glucose 6-phosphate (G6P, 25 mM) or (E) pyruvate (25 mM) at the indicated time points. Cell lysates were immunoprecipitated with FLAG antibody (IP:FLAG) and analyzed by immunoblotting with the indicated antibodies. (F) WT, TCF25^−/−^, and TCF25 reconstituted MDFs were starved without glucose at the indicated time points. The physical associations between ATP6V1A and ATP6V0D1 were detected as red spots by PLA assay. Left, representative confocal images of the cells are shown. Right, statistical analysis of the PLA punctum is shown. Scale bar, 20 μm. (G) HEK293T cells were transfected with HA-ATP6V1A with/out FLAG-TCF25 as indicated and then starved without glucose at the indicated time points. Left, cell lysates were immunoprecipitated with HA antibody (IP:HA) and analyzed by immunoblotting with the indicated antibodies. Right, quantification analysis of co-precipitated HA-ATP6V1A with endogenous ATP6V0D1 as described in the STAR Methods. (H) WT and TCF25^−/−^ MDFs stably expressed TMEM192-RFP-3×HA were starved without glucose at the indicated time points. Lysosomes were immunoprecipitated with anti-HA magnetic beads as described in the STAR Methods. Left, lysosomes were immunoprecipitated with HA antibody (IP:HA) and analyzed by immunoblotting with the indicated antibodies. Ponceau S staining was used as a loading control. Right, quantification analysis of ATP6V1A and ATP6V0D1 expression in the lysosome fraction as described in the STAR Methods. (I) WT and TCF25^−/−^ MDFs stably expressed HA-TMEM192 were starved without glucose at the indicated time points and then immunoprecipitated with HA antibody (IP:HA) to purify lysosome (Lyso-IP). The total cell lysates (input) and purified lysosome fraction were analyzed by immunoblotting with HA antibody. (J) The activities of V-ATPase in the lysosome fraction were measured as described in the STAR Methods. All western data are representative of three independent experiments. Bar graphs represent the mean ± SD from three independent experiments. Statistical analysis was performed using a two-sided student’s t test. The levels of significance are indicated by **p < 0.01; ***p < 0.001; n.s., not significant (two-way ANOVA).

As we found TCF25 is required for lysosomal acidification under glucose starvation and lysosomal acidity is mainly regulated by V-ATPase complex, we then asked whether TCF25 was able to interact with the components of V-ATPase in response to glucose starvation. V-ATPase is a large multi-subunit complex composed of a cytosolic ATP-hydrolyzing V1 domain and a membrane-embedded V0 proton channel.^12^ We first expressed FLAG-tagged TCF25, hemagglutinin (HA)-tagged V-ATPase V1 subunit A (ATP6V1A) or Myc-tagged V0 subunit D1 (ATP6V0D1) in HEK293T cells and examined the potential interaction between TCF25 and ATP6V1A or ATP6V0D1. We found that ATP6V1A, but not ATP6V0D1, was co-precipitated with TCF25 in response to glucose starvation (Figures 3A, S4A, and S4B). TCF25 contains a predicted DNA-binding bHLH domain and a conserved domain of unknown function named DUF654.^21,22^ To determine which TCF25 domain interacts with ATP6V1A, we co-expressed HA-tagged ATP6V1A and FLAG-tagged full-length TCF25, its N-terminal bHLH domain, or its C-terminal DUF654 domain in HEK293T cells. Under glucose starvation, both full-length TCF25 and the bHLH domain, but not the DUF654 domain, coimmunoprecipitated with ATP6V1A, indicating that the bHLH domain mediates this interaction (Figure S4C). We next tested whether TCF25 interacts with other V1 subunits. Co-immunoprecipitation in HEK293T cells expressing FLAG-tagged TCF25 and HA-tagged ATP6V1B2, ATP6V1G1, or ATP6V1H revealed that only ATP6V1A, but not the other V1 subunits, associated with TCF25 under glucose starvation (Figures S4D, S4E, and S4F). Furthermore, the endogenous interactions between TCF25 and ATP6V1A were confirmed in both MDF and HeLa cells (Figures 3B and S4G). Additionally, when glucose level was lower than the normal blood glucose concentration of 5 mM, the association between TCF25 and ATP6V1A was progressively increased (Figure 3C). Importantly, the replenishment of intermediates in glycolysis, glucose-6-phosphate (G6P) or pyruvate, in glucose-deprived medium suppressed the association between TCF25 and ATP6V1A (Figures 3D and 3E). Additionally, the association between TCF25 and ATP6V1A was specifically detected under glucose starvation but not amino acid deprivation (Figure S4H). Together, our data suggest that glucose starvation specifically induces the association between TCF25 and ATP6V1A, which is most likely regulated by signaling pathways in glycolysis.

Prompted by the association of TCF25 with cytosolic ATP6V1A subunit under glucose starvation, we then investigated whether TCF25 was able to regulate the activity of the V-ATPase. Among various regulatory modes of V-ATPase activity, the most important is the regulation of cytosolic V1 domain (V1A) association with membrane-bound V0 domain (V0D) in V-ATPase complex.^12,13^ Therefore, we first investigated whether TCF25 regulated the assembly of V1 and V0 domains of the V-ATPase complex in response to glucose starvation by proximity ligation amplification assay (PLA). Confocal imaging by using antibodies against ATP6V1A and ATP6V0D1 showed a marked increase of V1A-V0D1 association in WT and reconstituted TCF25^−/−^, but not TCF25 ^−/−^, MDFs in response to glucose starvation (Figure 3F). Moreover, overexpression of TCF25 in HEK293T cells increased the association between HA-ATP6V1A and endogenous ATP6V0D1 under glucose starvation (Figure 3G). Thus, these results indicate that TCF25 promotes the assembly of V-ATPase under glucose starvation. We then used lysosome immunoprecipitation (Lyso-IP) followed by lysosomal fractionation analysis. We observed increased ATP6V1A levels in the lysosomal fraction upon glucose starvation. This increase was attenuated by TCF25 knockout (Figure 3H). Therefore, our findings suggest that TCF25 facilitates ATP6V1A delivery to lysosomes through their association. Finally, the hydrolysis activity of lysosomal V-ATPase was investigated and we found concanamycin A (ConA)-sensitive ATPase activity was increased by glucose starvation in the immunopurified V-ATPase complex from WT but not TCF25 ^−/−^ MDFs (Figures 3I and 3J). Taken together, these data indicate that TCF25 enhances lysosomal acidification by increasing the ATPase activity of the V1 domain in the assembled V-ATPase complex under glucose starvation.

### TCF25 promotes lysosomal degradative activity to maintain cellular energy balance during short-term glucose starvation

**Figure 4.**
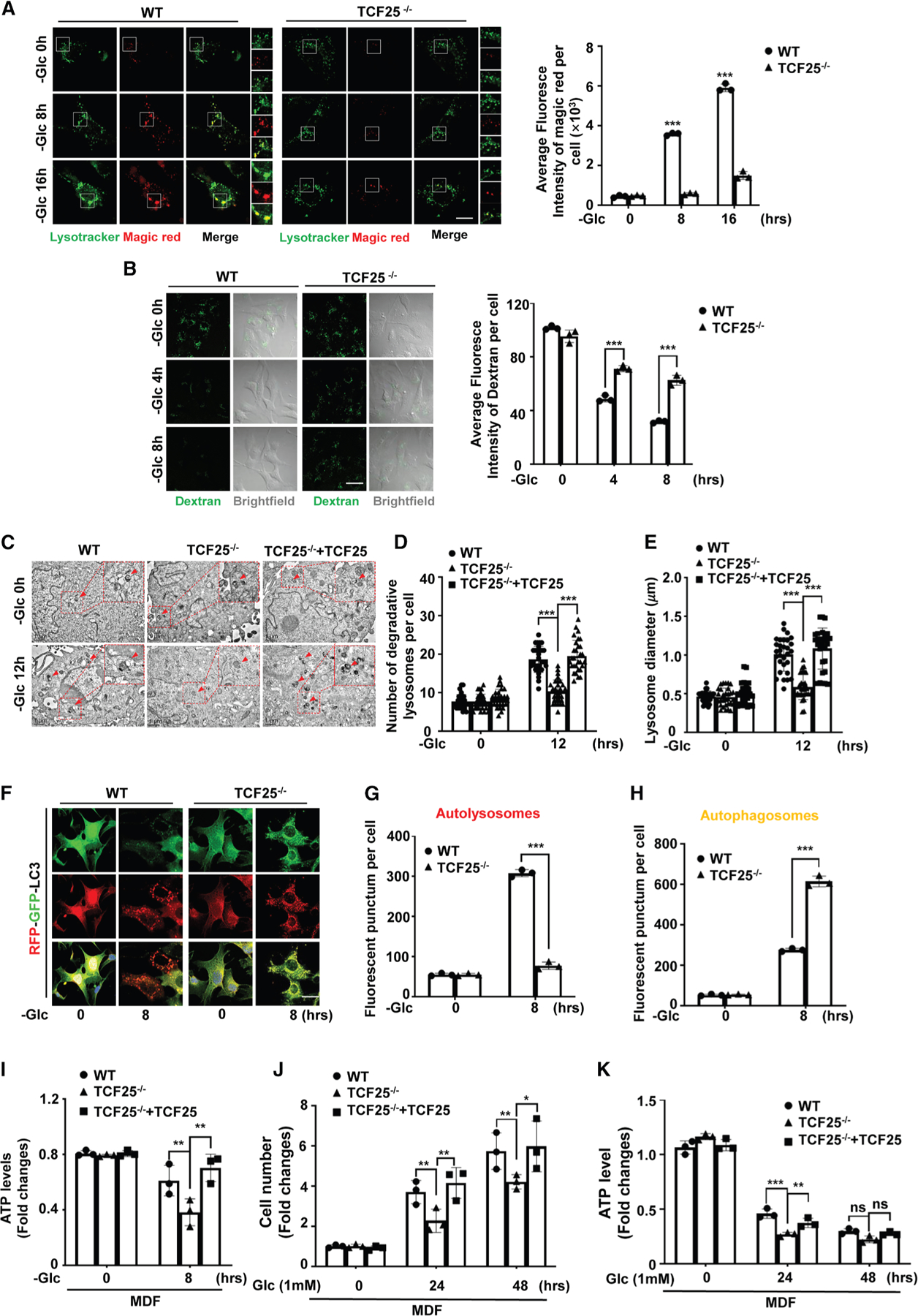
TCF25 promotes lysosomal degradative activity to maintain cellular energy balance during short-term glucose starvation (A) WT and TCF25^−/−^ MDFs were starved without glucose at the indicated time points and then stained with Magic Red and LysoTracker Green. Left, representative confocal images of the cells are shown. Right, statistical analysis of the Magic Red fluorescence intensity is shown. Scale bar, 20 μm. (B) WT and TCF25^−/−^ MDFs were loaded with Alexa Fluor 488-dextran for 20 min and then starved without glucose at the indicated time points. Left, representative confocal images of the cells are shown. Right, statistical analysis of the Alexa Fluor 488-dextran fluorescence intensity is shown. Scale bar, 20 μm. (C) WT, TCF25^−/−^, and TCF25 reconstituted MDFs were starved without glucose at the indicated time points. Representative transmission electron microscopy (TEM) images of the cells are shown. Lysosomes are indicated by red arrow. Scale bar, 1 μm. (D) Quantification of lysosome number of in (C). A total of 30 cells from three independent experiments were analyzed. (E) Quantification of lysosome size in (C). A total of 30 cells from three independent experiments were analyzed. (F) WT and TCF25^−/−^ MDFs stably expressed RFP-GFP-LC3 were starved without glucose at the indicated time points. Representative confocal images of the cells are shown. Scale bar, 20 μm. (G) Statistical analysis of autolysosomes in (F). (H) Statistical analysis of autophagosome in (F). (I) WT, TCF25^−/−^, and TCF25 reconstituted MDFs were starved without glucose at the indicated time points. The relative ATP levels were measured by ATP-Glo bioluminometric assay and normalized to cell number. (J) WT, TCF25^−/−^, and TCF25 reconstituted MDFs were starved with low glucose (1 mM) at the indicated time points. The relative cell number is shown. (K) The relative ATP levels of MDFs in (J) were measured by ATP-Glo bioluminometric assay and normalized to cell number. Bar graphs represent the mean ± SD from three independent experiments. Statistical analysis was performed using a two-sided student’s t test. The levels of significance are indicated by *p < 0.05; **p < 0.01; ***p < 0.001; n.s., not significant (two-way ANOVA).

Lysosomal acidification is critical for its degradative function since most of the hydrolytic enzyme resident in lysosomes such as cathepsins are activated at low pH.^12^ We then measured CTSB activity, which is a major cysteine protease involved in lysosomal proteolysis,^10^ in both WT and TCF25^−/−^ MDFs to investigate whether its activity was regulated by TCF25 under glucose starvation. By using the Magic Red CTSB assay, which is based on an engineered CTSB substrate that generates red fluorescence upon cleavage, we found CTSB activity was time-dependently increased during glucose starvation in WT MDFs, while its protease activity was only marginally increased in TCF25^−/−^ MDFs (Figure 4A), indicating TCF25 is required for increased CTSB activity under glucose starvation. We further examined lysosomal degradative activity by using a pH-insensitive Alexa Fluor 488-dextran probe, which can be taken up by the cells through endocytosis and degraded in the lysosomes.^27^ We found glucose starvation time-dependently decreased the fluorescence intensity of lysosomal dextran conjugated to Alexa Fluor 488 in WT MDFs (Figure 4B), indicating the activation of lysosomal degradative activity. However, TCF25^−/−^ MDFs had longer half-life of lysosomal dextran fluorescence compared to WT MDFs under glucose starvation (Figure 4B), suggesting TCF25 is required for lysosomal degradative activity in response to glucose starvation. Furthermore, by examining lysosomes with transmission electron microscopy, we found degradative lysosomes, which were indicated as large electron-dense organelles,^28^ were significantly accumulated in glucose-starved WT and TCF25-reconstituted MDFs, while these degradative lysosomes were barely observed in starved TCF25^−/−^ MDFs (Figures 4C–4E). Together, these data indicate that TCF25 contributes to enhanced lysosomal degradative function by promoting V-ATPase-dependent lysosomal acidification under glucose starvation.

The early response to glucose starvation is the activation of AMPK.^2^ The activated AMPK suppresses mTOR complex 1 (mTORC1) activity to positively regulate autophagy and activate catabolism under glucose starvation.^29,30^ We then examined AMPK and mTORC1 activities in both WT and TCF25^−/−^ MDFs by performing western blotting for phosphorylation of AMPK (p-AMPK) and mTORC1 (p-mTORC1), respectively. Consistent with previous studies,^14^ p-AMPK increased and p-mTORC1 decreased in WT MDFs under glucose starvation, and these responses were unaffected by TCF25 knockout (Figure S5A). These results indicate that TCF25 does not modify the activation status of AMPK signaling in response to glucose starvation.

As we demonstrate that TCF25 targets V-ATPase to enhance lysosomal acidification under glucose starvation and the V-ATPase activity is required for proper autophagic flux,^15,31^ we then investigated whether autophagy was affected by TCF25 in response to glucose starvation. By examining the conversion of LC3-I to LC3-II, a hallmark of autophagy, we showed that TCF25^−/−^ MDFs have more accumulated LC3-II during glucose starvation as compared to WT MDFs (Figure S5B). Since the accumulation of LC3-II may represent either an increase in autophagosome formation or a block of autophagic flux,^32^ we then treated cells with lysosomal inhibitor BafA1 to block lysosomal degradation of autophagosomes. Under this condition, we found glucose starvation increased expression of LC3-II in WT but not TCF25^−/−^ MDFs (Figure S5C), suggesting TCF25 is required for autophagic flux under glucose starvation. To investigate whether TCF25 is required for autolysosome formation, we transfected the mCherry-LC3 plasmid into both WT and TCF25^−/−^ MDFs and starved these cells without glucose for 8 h. Subsequent co-localization analysis using LysoTracker Green revealed distinct patterns: while the majority of mCherry-LC3 puncta co-localized with lysosomal compartments in WT MDFs under glucose starvation, this co-localization was significantly reduced in TCF25^−/−^ cells (Figure S5D), suggesting TCF25 may contribute to autolysosome formation during glucose deprivation. We further measured the quantity of autophagosomes and autolysosomes with mRFP-GFP-LC3 reporter.^32^ Under glucose starvation, we found autolysosomes (red-fluorescent puncta) were predominant in WT MDFs (Figures 4F–4H). However, in starved TCF25^−/−^ MDFs, the formation of autolysosomes was inhibited and autophagosomes (yellow-fluorescent puncta) were predominant (Figures 4F–4H), suggesting TCF25 is crucial for autolysosome formation in response to glucose starvation.

A previous study suggest that autophagy is a critical energy provider during glucose starvation.^33^ We then examined whether TCF25 was able to regulate intracellular ATP levels, the main energy source used by cells, under glucose starvation. It is known that glucose starvation decreases ATP production.^34^ We found the intracellular ATP level was dramatically decreased in TCF25^−/−^ MDFs as compared to that in WT MDFs when these cells were starved without glucose for 8 h (Figure 4I). Similar results were also observed in HeLa cells (Figure S5E). Importantly, reconstituted expression of TCF25 in TCF25^−/−^ MDFs restored ATP level to that in WT MDFs under glucose starvation (Figure 4I). These data suggest that TCF25 is critical for energy maintenance under short-term glucose starvation. Additionally, it is unlikely that glucose-starvation-induced cell death is caused by ATP depletion because the ATP level in WT MDFs was even higher than that in TCF25^−/−^ MDFs under glucose starvation (Figure 4I).

Cells with high ATP level typically indicate high cellular viability. To determine whether TCF25 can maintain cell viability under low-glucose conditions, we starved MDFs with low glucose (1 mM) and measured cell proliferation by counting the cell number. Under such low-glucose conditions, the cells did not die and could grow for a while (Figure 4J). Interestingly, TCF25 knockout significantly retarded cell proliferation as compared to WT MDFs under low-glucose conditions, while reconstituted expression of TCF25 in TCF25^−/−^ MDFs restored cell growth (Figure 4J). Furthermore, the intracellular ATP level in TCF25^−/−^ MDFs was lower than that in WT cells under low-glucose conditions (Figure 4K). Together, these results indicate that TCF25 is crucial for energy maintenance under low-glucose conditions by promoting autophagic-lysosomal activity.

### Prolonged glucose starvation increases LMP to induce LDCD, which is dependent on TCF25-mediated ferritinophagy

**Figure 5.**
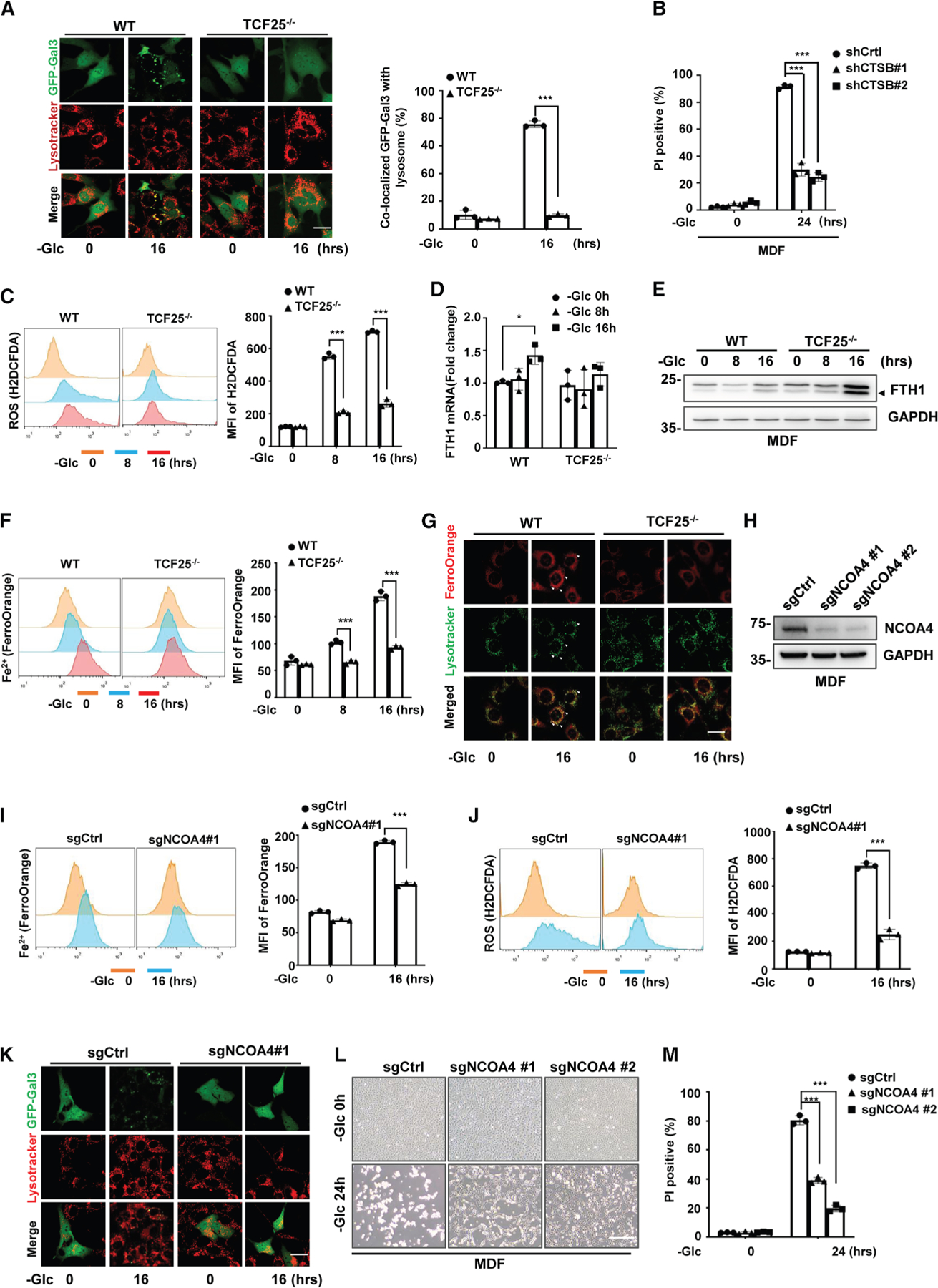
Sustained glucose starvation increases LMP to induce LDCD, which is dependent on TCF25-regulated ferritinophagy (A) WT and TCF25^−/−^ MDFs were transfected with GFP-Gal3 and then starved without glucose at the indicated time points. The cells were stained with LysoTracker Red and the representative confocal images are shown. Right, statistical analysis of the co-localized GFP-Gal3 with LysoTracker Red is shown. Scale bar, 20 μm. (B) shCtrl, shCTSB#1, and shCTSB#2 MDFs were starved without glucose at the indicated time points and the cell death was determined by PI staining. (C) WT and TCF25^−/−^ MDFs were starved without glucose at the indicated time points. The cells were stained with ROS indicator H2DCFDA. Left, representative flow cytometry histograms are shown. Right, mean fluorescence intensity (MFI) of the cells was analyzed. (D) Relative mRNA levels of FTH1 in the cells from (C) was determined by real time-qPCR. (E) The cells in (C) were lysed and immunoblotted with FTH1 antibody. (F) The cells in (C) were stained with Fe^2+^ indicator FerroOrange. Left, representative flow cytometry histograms are shown. Right, MFI of the cells was analyzed. (G) WT and TCF25^−/−^ MDFs were starved without glucose for 16 h and then co-stained with FerroOrange and LysoTracker Green. Representative confocal images of the cells are shown. (H) MDFs were stably transfected with sgRNA-Control (sgCtrl) and two individual sgRNAs targeting NCOA4 (sgNCOA4 #1 and sgNCOA4 #2), respectively. NCOA4 expression was examined by immunoblotting with its specific antibody. (I) sgCtrl and sgNCOA4#1 MDFs were starved without glucose at the indicated time points. The cells were stained with Fe^2+^ indicator FerroOrange. Left, representative flow cytometry histograms are shown. Right, MFI of the cells was analyzed. (J) The cells in (I) were stained with ROS indicator H2DCFDA. Left, representative flow cytometry histograms are shown. Right, MFI of the cells was analyzed. (K) sgCtrl and sgNCOA4#1 MDFs were transfected with GFP-Gal3 and then starved without glucose at the indicated time points. The cells were stained with LysoTracker Red and the representative confocal images are shown. Scale bar, 20 μm. (L) sgCtrl, sgNCOA4#1, and sgNCOA4#2 MDFs were starved without glucose for 24 h and the representative images are shown. Scale bar, 100 μm. (M) Cell death of the MDFs cells in (L) was determined by PI staining. Bar graphs represent the mean ± SD from three independent experiments. Statistical analysis was performed using a two-sided student’s t test. The levels of significance are indicated by ***p < 0.001; n.s., not significant. (two-way ANOVA).

We showed that TCF25 was critical for cells to preserve energy balance during short-term glucose starvation; however, it seemed odds that TCF25 also mediated glucose-starvation-induced cell death (Figures 1E, 1G, and 1L). Our CRISPR-Cas9 screening identified that the sgRNA-targeting gene encoding lysosomal cathepsin protease CTSB was significantly enriched after being starved without glucose (Figure 2E). CTSB has been shown to play essential roles in the execution of LDCD,^35^ which is a type of PCD that is triggered by increased LMP, resulting in the release of cathepsins and other hydrolases from the lysosomal lumen to the cytosol.^36,37^ We therefore speculated that LDCD might occur under sustained glucose starvation. We first assessed the involvement of LMP in glucose-starvation-induced cell death by performing a galectin-3 (Gal3) punctum assay. Because cytosolic galectins can translocate to the membrane of leaky lysosomes, the co-localization of lysosomes with galectin puncta is used as an indicator of LMP.^38^ We transfected cells with GFP-Gal3 construct and monitored the GFP-Gal3 punctum formation during glucose starvation. We found that GFP-Gal3 puncta were formed and co-localized with lysosomes in WT MDFs after being starved without glucose for 16 h; however, we could not detect the formation of GFP-Gal3 puncta in TCF25^−/−^ MDFs (Figure 5A). Similar results were also observed in HeLa cells (Figure S6A). Thus, these data reveal that sustained glucose starvation increases LMP, which is dependent on TCF25.

We then examined the contribution of CTSB in glucose-starvation-induced cell death by knockdown of CTSB in both MDF and HeLa cells (Figures S6B and S6D). We found knockdown of CTSB protected cells from glucose-starvation-induced cell death (Figures 5B, S6C, S6E, and S6F). Additionally, CTSB inhibitor CA074Me and nitroxoline attenuated glucose-starvation-induced cell death (Figures S6G–S6J). By examining the cleavage of Casp-3 and phosphorylation of MLKL, we showed that both glucose-starvation-induced apoptosis and necroptosis were inhibited by CTSB knockout (Figure S6K), suggesting CTSB acts as an upstream regulator of apoptosis and necroptosis under glucose starvation. Thus, these data indicate that LDCD is the main form of PCD in these cells under glucose starvation.

We then planned to investigate how LMP was induced by glucose starvation. LMP can be induced by intralysosomal redox-active iron,^39^ reactive oxygen species (ROSs),^40^ or other cellular stresses.^41^ We found glucose starvation time-dependently increased ROS levels in MDFs (Figure 5C). However, the production of ROSs was significantly inhibited by TCF25 knockout (Figure 5C). We then investigated how TCF25 regulated ROS production during glucose starvation. Although it has been reported that glucose starvation led to an increase in mitochondrial ROSs,^42^ we found mitochondrial superoxide production did not significantly increase in MDFs during glucose starvation (Figure S7A), suggesting TCF25 must regulate ROS generation from other sources. Intracellular ROS can be generated from a type of selective autophagy named ferritinophagy, which uses a cargo receptor nuclear receptor coactivator 4 (NCOA4) to deliver iron storage protein ferritin for lysosomal degradation.^43,44^ Ferritinophagy results in the release of labile iron (Fe^2+^) in cells and generates massive ROS via the Fenton reaction.^45,46^ As glucose starvation induces autophagy, we then investigated whether ferritinophagy was simultaneously activated by glucose starvation through examining the expression of ferritin heavy chain 1 (FTH1), a substrate of ferritinophagy, in glucose starved cells. Given FTH1 is transcriptionally upregulated by Fe^2+^,^47,48^ we firstly measured the mRNA level of FTH1 by RT-PCR. We found FTH1 mRNA increased in WT but not TCF25^−/−^ MDFs under prolonged glucose starvation for 16 h (Figure 5D). Remarkably, FTH1 protein expression did not change significantly in WT MDFs during glucose starvation; however, its protein level was dramatically increased in TCF25^−/−^ MDFs after being starved without glucose for 16 h (Figure 5E). Thus, these data suggest that FTH1 is degraded by ferritinophagy during glucose starvation, while TCF25 knockout prevents FTH1 degradation by inhibiting ferritinophagy. We then measured the level of intracellular ferrous iron by FerroOrange staining and found glucose starvation led to the accumulation of Fe^2+^ iron in WT but not TCF25^−/−^ MDFs (Figure 5F), indicating TCF25 is essential for intracellular ferrous iron accumulation under glucose starvation. Notably, FerroOrange imaging demonstrated that Fe^2+^ iron was significantly accumulated in lysosome after being starved without glucose in WT but not TCF25^−/−^ MDFs (Figure 5G). To finally pinpoint whether TCF25-regulated ferritinophagy contributes to glucose-induced cell death, we knocked out NCOA4 by CRISPR-Cas9 to block ferritinophagy in MDFs (Figure 5H). Under prolonged glucose starvation, we found that knockout of NCOA4 significantly inhibited ferrous iron accumulation (Figure 5I), ROS production (Figure 5J), LMP (Figure 5K), and cell death (Figures 5L and 5M). Additionally, knockdown of NCOA4 also prevented glucose-starvation-induced cell death in HeLa cells (Figures S7B–S7D). This demonstrates that NCOA4-mediated ferritinophagy is crucial for glucose-induced cell death. Furthermore, glucose-starvation-induced cell death could be attenuated by treating MDFs or HeLa cells with iron chelators, such as deferoxamine (DFO) and deferiprone (DFP), or ROS scavenger Trolox (Figures S7E–S7J), suggesting a critical role of redox-active iron in mediating glucose-starvation-induced cell death in these cells. Taken together, these findings reveal that TCF25 is critical for energy maintenance by promoting lysosomal catabolic activity in low-glucose conditions; however, if glucose starvation is sustained, TCF25-regulated lysosomal-autophagic activity is essential for ferritinophagy and its constitutive activation will increase LMP to eventually kill the cells by LDCD.

Recent studies identified that cells with high expression of cystine transporter cystine transporter solute carrier family 7 member 11 (SLC7A11) were highly sensitive to glucose-starvation-induced cell death.^49,50^ These cells died from a distinct form of PCD named disulfidptosis, which results from the accumulation of intracellular cystine and formation of aberrant disulfide bonds in actin cytoskeleton proteins.^50^ We found SLC7A11 expression is very low in HeLa, HT-29, and MDF cells (Figure S8A), suggesting these cells cannot be die from disulfidptosis under glucose starvation. Furthermore, we found TCF25 knockout prevented glucose-starvation-induced cell death in SLC7A11 low-expressed 786-O cells (Figures S8B and S8C) but not SLC7A11 high-expressed Skov3 cells (Figures S8D and S8E), indicating TCF25 is not involved in glucose-starvation-induced cell death in SLC7A11 high-expressed cells. Additionally, we showed that CTSB inhibitors such as CA074Me and nitroxoline attenuated glucose-starvation-induced cell death in 786-O, HT-29, and HEK293T cells, all of which are SLC7A11 low-expressed cells (Figures S8F–S8H). Therefore, these data indicate that TCF25 is not involved in disulfidptosis and it might specifically mediate glucose-starvation-induced cell death in SLC7A11 low-expressed cells.

### TCF25-V-ATPase signaling axis is essential for glucose-starvation-induced cell death

**Figure 6.**
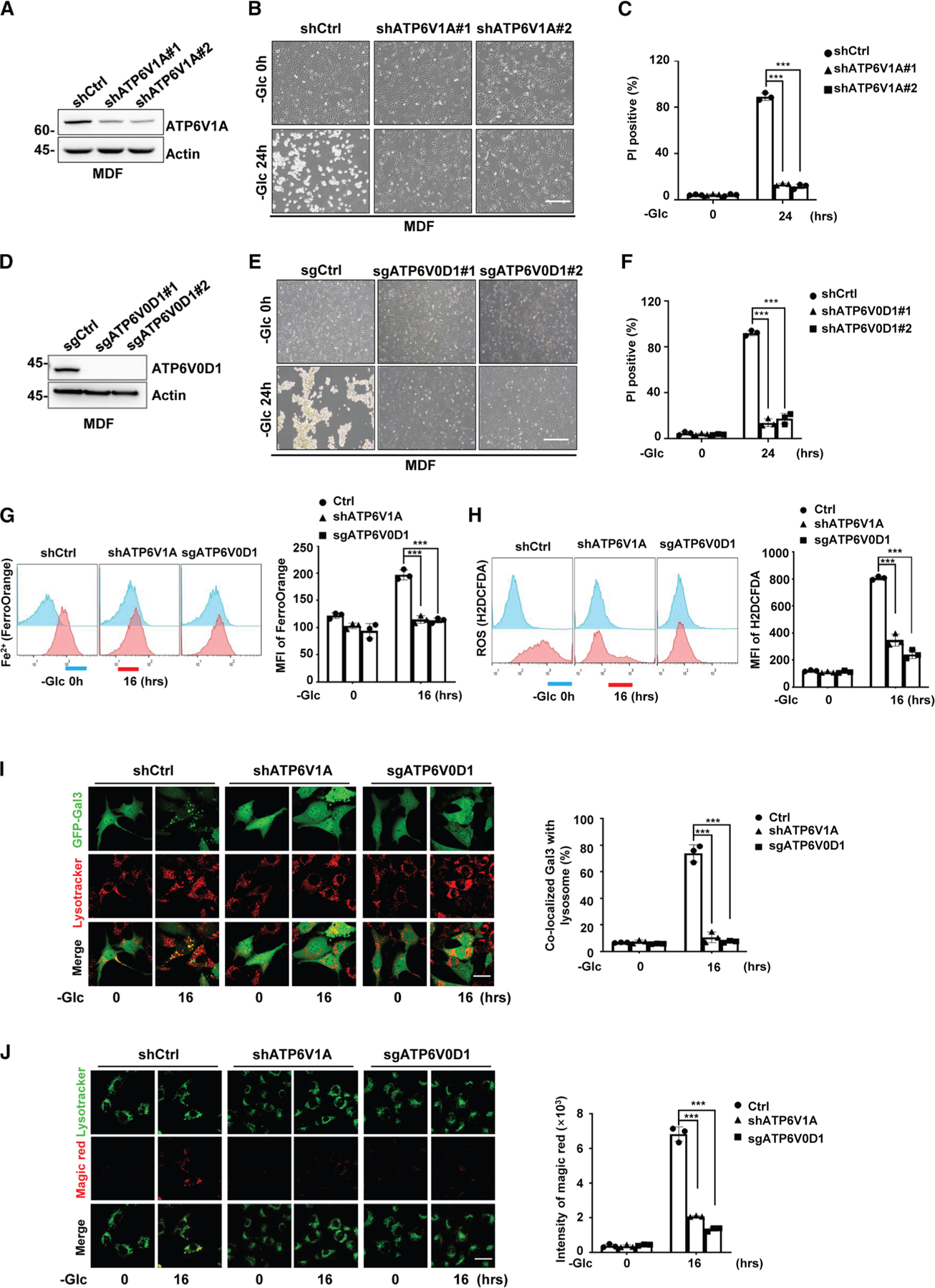
TCF25-V-ATPase signaling axis is essential for glucose-starvation-induced cell death (A) MDFs were stably transfected with shRNA-Control (shCtrl) and two individual shRNAs targeting ATP6V1A (shATP6V1A #1 and shATP6V1A #2), respectively. ATP6V1A expression was examined by immunoblotting with its specific antibody. (B) The cells in (A) were starved without glucose at the indicated time points and the representative images are shown. Scale bar, 100 μm. (C) Cell death of the MDFs in (A) was determined by PI staining. (D) MDFs were stably transfected with sgRNA-Control (sgCtrl) and two individual sgRNAs targeting ATP6V0D1 (sgATP6V0D1#1 and sgATP6V0D1#2), respectively. ATP6V0D1 expression was examined by immunoblotting with its specific antibody. (E) The cells in (D) were starved without glucose at the indicated time points and the representative images are shown. Scale bar, 100 μm. (F) Cell death of the MDFs in (D) was determined by PI staining. (G) shCtrl, shATP6V1A, and sgATP6V0D1 MDFs were starved without glucose for 16 h. The cells were stained with Fe^2+^ indicator FerroOrange. Left, representative flow cytometry histograms are shown. Right, mean fluorescence intensity (MFI) of the cells was analyzed. (H) shCtrl, shATP6V1A, and sgATP6V0D1 MDFs were starved without glucose for 16 h. The cells were stained with ROS indicator H2DCFDA. Left, representative flow cytometry histograms are shown. Right, MFI of the cells was analyzed. (I) shCtrl, shATP6V1A, and sgATP6V0D1 MDFs were transfected with GFP-Gal3 and then starved without glucose for 16 h. The cells were stained with LysoTracker Red. Left, representative confocal images of the cells are shown. Right, statistical analysis of the co-localized GFP-Gal3 with LysoTracker Red is shown. Scale bar, 20 μm. (J) shCtrl, shATP6V1A, and sgATP6V0D1 MDFs were starved without glucose for 16 h and then stained with Magic Red and LysoTracker Green. Left, representative confocal images of the cells are shown. Right, statistical analysis of the Magic Red fluorescence intensity is shown. Scale bar, 20 μm. Bar graphs represent the mean ± SD from three independent experiments. Statistical analysis was performed using a two-sided student’s t test. The levels of significance are indicated by ***p < 0.001; n.s., not significant (two-way ANOVA).

We showed that TCF25 localized to lysosome and enhanced lysosomal acidification by targeting V-ATPase complex in response to glucose starvation. As our CRISPR-Cas9 screen identified several components of V-ATPase complex that might contribute to glucose-starvation-induced cell death (Figure 2E), we then planned to confirm whether V-ATPase functioned as a TCF25 downstream component to mediate glucose-starvation-induced cell death. We found either short hairpin RNA (shRNA)-mediated knockdown of ATP6V1A or CRISPR-Cas9-mediated knockout of ATP6V0D1, the two essential components of V-ATPase complex, protected cells from glucose-starvation-induced cell death (Figures 6A–6F, S9A, and S9B). Additionally, we reconstituted ATP6V0D1 expression in ATP6V0D1 knockout cells and we found glucose-starvation-induced cell death was re-established (Figures S9C and S9D). By examining the cleavage of Casp-3 and phosphorylation of MLKL, we found that both glucose-starvation-induced apoptosis and necroptosis were inhibited by ATP6V1A knockdown or ATP6V0D1 knockout, respectively (Figures S9E and S9F). Additionally, ATP6V1A knockdown or ATP6V0D1 knockout also prevented glucose transporter 1 inhibitor BAY-876-induced cell death (Figures S9G–S9J). Finally, we showed that glucose-starvation-induced ferrous iron accumulation (Figure 6G), ROS production (Figure 6H), LMP (Figures 6I and S9K), and CTSB activity (Figure 6J) were significantly attenuated by knocking down ATP6V1A or knocking out ATP6V0D1. Together, these results indicate that glucose-starvation-induced cell death is regulated by TCF25-V-ATPase signaling axis.

### TCF25 deficiency protects mice from hepatic IRI

**Figure 7.**
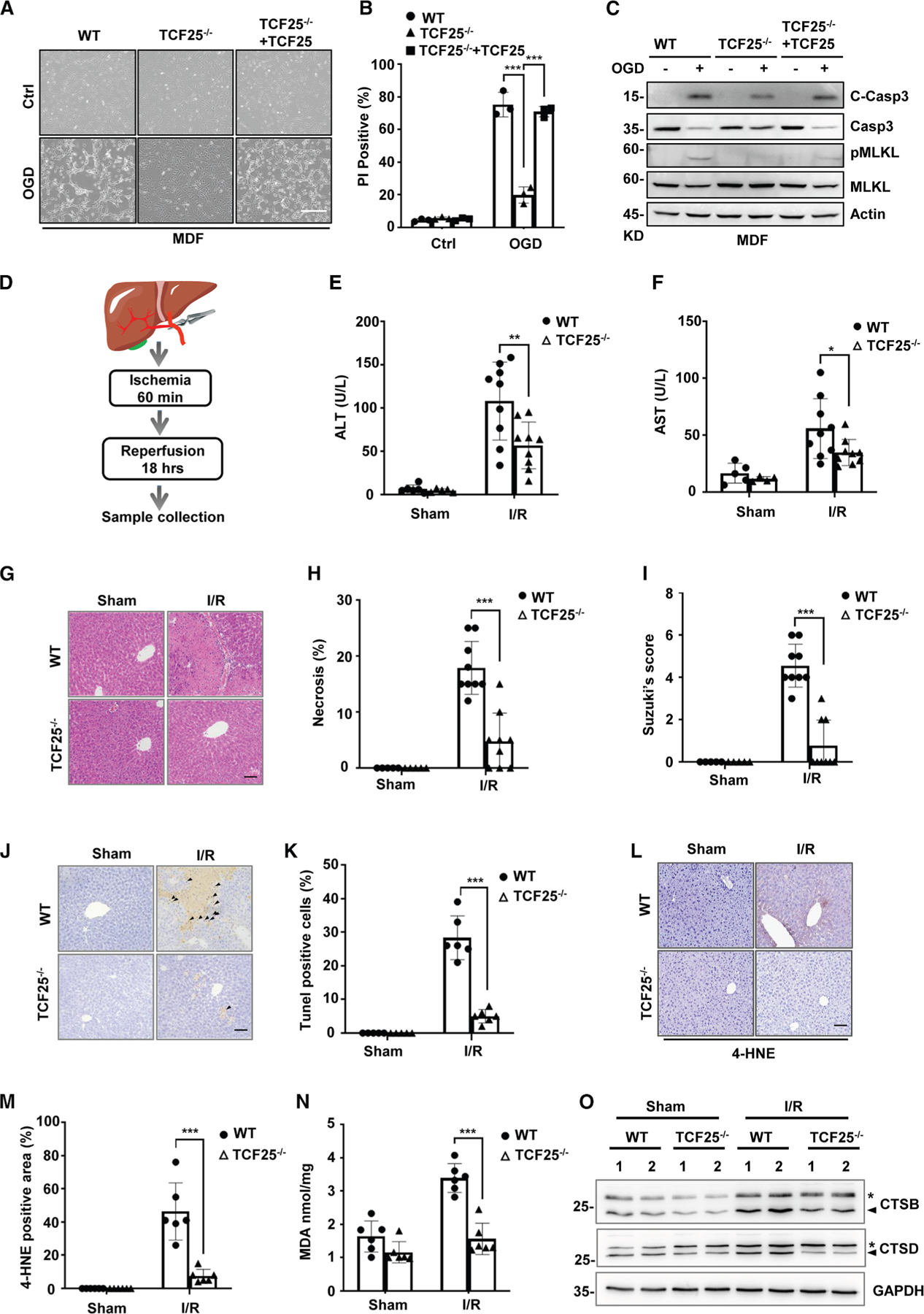
TCF25 deficiency protects mice from hepatic IRI (A) WT, TCF25^−/−^, and TCF25 reconstituted MDFs were treated with oxygen-glucose deprivation (OGD) for 12 h and the representative images are shown. Scale bar, 100 μm. (B) Cell death of the MDFs in (A) was determined by PI staining. (C) The MDFs in (A) were lysed and immunoblotted with the indicated antibodies. (D) Schematic of liver IR injury involving 60-min global ischemia followed by an 18-h reperfusion period. (E and F) Serum ALT (E) and AST levels (F) were measured from WT and TCF25^−/−^ mice subjected to liver IR injury. (G) Representative liver H&E images from the mice in (E). Scale bar, 50 μm. (H) Quantification of liver necrosis from the liver H&E images in (G). (I) Suzuki’s histological grading of the liver from the mice in (E). (J) Representative images of TUNEL staining in livers from the mice in (E). Scale bar, 50 μm. (K) Quantification of TUNEL-positive cells in (J). (L) Representative images of 4-HNE staining in livers from the mice subjected to IR injury. Scale bar, 50 μm. (M) Quantification of 4-HNE-positive cells in (L). (N) The levels of MDA in liver homogenate were measured from WT and TCF25^−/−^ mice subjected to liver IR injury. (O) Immunoblotting analysis of CTSB and CTSD in freshly isolated livers from the mice in (E). Asterisk indicates pro-form; black arrowhead indicates active form. All western data are representative of three independent experiments. Statistical analysis was performed using a two-sided student’s t test. The levels of significance are indicated by ***p < 0.001; n.s., not significant (two-way ANOVA).

Oxygen-glucose deprivation (OGD)-induced cell death is widely used as an *in vitro* model for ischemia. We then tested whether TCF25 contributed to OGD-induced cell death. We found OGD treatment led to a remarkable cell death in WT MDFs, which was significantly attenuated in TCF25^−/−^ MDFs and re-activated by reconstituted expression of TCF25 in TCF25^−/−^ MDFs (Figures 7A and 7B). Furthermore, TCF25 knockout potently attenuated OGD-induced apoptosis and necroptosis in MDFs, as illustrated by the cleavage of Casp-3 for apoptosis and phosphorylation of MLKL for necroptosis (Figure 7C). Thus, these data indicate that loss of TCF25 protects cells from OGD-induced cell death *in vitro*.

Since TCF25 is highly expressed in mouse liver (Figure S2C), we then investigated the functional role of TCF25 in mice model of hepatic IRI (Figure 7D). Notably, we found TCF25^−/−^ mice showed decreased alanine transaminase (ALT) and glutamic oxaloacetic transaminase (AST) levels in serum as compared to WT mice after hepatic IR (Figures 7E and 7F). H&E staining for livers showed that congestion, vacuolation, and necrosis all decreased significantly in TCF25^−/−^ mice in comparison to those in the liver of WT mice (Figures 7G and 7H). These data were further confirmed by decreased Suzuki scores in TCF25^−/−^ mice as compared with WT mice (Figure 7I). Additionally, as illustrated by TUNEL staining, the hepatocyte apoptosis was also decreased by TCF25 knockout after hepatic IR (Figures 7J and 7K). We then analyzed two *in vivo* lipid peroxidation markers of ferroptosis, 4-hydroxynonenal (4-HNE) and malondialdehyde (MDA), in liver tissues. Our results showed that hepatic I/R injury in WT mice elevated both 4-HNE and MDA levels, whereas TCF25 knockout markedly suppressed these increases (Figures 7L–7N), suggesting TCF25 contributes to ferroptosis in I/R-induced liver injury. Furthermore, we found the active forms of cathepsins, including CTSB and CTSD, were increased by hepatic IR in the liver of WT mice, while the increase of CTSB and CTSD was inhibited by TCF25 knockout (Figure 7O). Taken together, these data suggest that TCF25 contributes to hepatic IRI in mice.

## DISCUSSION

Our data presented in this study suggest a cellular model in response to glucose starvation in which cells need to enhance V-ATPase-dependent lysosomal acidification to improve catabolism, and, if the starvation is sustained, the redox-active iron is accumulated through ferritinophagy, which results in LMP to eventually kill the cells. In this scenario, TCF25 serves as a nutrient sensor to enhance lysosomal acidification by targeting V-ATPase in response to glucose starvation. On the one hand, it is crucial for catabolism by increasing lysosomal activity under glucose starvation. On the other hand, TCF25-regulated lysosomal-autophagic activity is essential for ferritinophagy and its constitutive activation may cause LDCD during prolonged glucose starvation.

In the literature, glucose deprivation has been shown to activate different forms of cell death pathways, including apoptosis,^24^ necroptosis,^25^ and disulfidptosis,^50^ which may depend on genetic backgrounds of cells. Our data suggest that glucose-deprivation-induced cell death is essentially a form of LDCD for some of the cells. It is known that LMP contributes to apoptosis and various types of PCD, including necroptosis, pyroptosis, and ferroptosis.^36,37^ We therefore propose that disturbances in lysosome membrane integrity and function are critical for the initiation of downstream programmed cell death pathways under glucose starvation, as knockout of TCF25, V-ATPase components, or lysosomal hydrolase CTSB blocks glucose-starvation-induced apoptosis and necroptosis.

Although different cells can respond in different ways to glucose starvation, the essential effect of glucose starvation for each cell is to execute a coordinated metabolic reprogramming process to ensure cell survival in unfavorable conditions. However, metabolic reprogramming may generate toxic metabolic byproducts that contribute to cellular dysfunction when glucose starvation is sustained. The results presented here, together with other reports,^49,50^ suggest that toxic metabolic byproducts produced in glucose starvation, such as redox-active iron, ROSs, or cystine,^49^ contribute to cellular dysfunction and will ultimately kill the cells under sustained glucose starvation. Therefore, the threshold for cell tolerance to toxic metabolic byproducts and energy depletion under glucose starvation might determine whether and how cells die from glucose starvation. From this perspective, targeting or removing the toxic byproducts produced in glucose starvation might be a more effective way to prevent glucose-starvation-induced cell death and related disorders than targeting the signaling mechanism of programmed cell death itself.

TCF25 was originally identified as a nuclear-localized transcriptional factor containing a predicted DNA-binding bHLH domain and a conserved domain of unknown function named DUF654.^21,22^ As a nuclear-localized protein, it has been shown to alleviate cardiac hypertrophy by suppressing Nuclear factor of activated T cells 3 (NFAT3) transcriptional activity.^23^ Intriguingly, TCF25 is also involved in ribosome-associated quality control by association with ribosomal complexes,^51,52^ which indicates TCF25 can be functional as a cytoplasmic protein. Our study identifies TCF25 as a nutrient sensor that specifically respond to glucose starvation by translocation from cytoplasm to lysosome. TCF25 has no predicted lysosome-targeting or transmembrane sequences. It is unknown how TCF25 localizes to lysosomes in response to glucose starvation. The translocation of TCF25 could be regulated by post-translational modifications or other unidentified proteins that may interact with TCF25. Further investigations are required to elucidate how TCF25 localizes to lysosomal during glucose starvation and this might help us to uncover uncharacterized molecule(s) involved in the metabolic reprogramming process under nutrient stress.

In summary, our study identifies TCF25 as a nutrient sensor to promote catabolism by enhancing V-ATPase-dependent lysosomal acidification in response to glucose starvation. However, this process may contribute to cellular dysfunction and disease pathology when glucose starvation is sustained, as the constitutive activation of TCF25-mediated ferritinophagy will cause LMP to eventually kill the cells. This work highlights the interplay between metabolic adaptation and cell-death regulation under glucose starvation, which will be helpful in rational design of therapeutic treatments for metabolic and ischemic disorders.

### Limitations of the study

This study has several limitations. First, the mechanism of TCF25 lysosomal localization under glucose starvation remains unclear. Co-immunoprecipitation coupled with mass spectrometry (coIP/MS) to identify TCF25 post-translational modifications (PTMs) and binding partners would provide critical insights. Second, although TCF25 and ATP6V1A form a complex specifically during glucose deprivation, direct interaction between these proteins remains unconfirmed. This context-dependent association suggests PTM-mediated regulation, a hypothesis currently under investigation. Third, while our data indicate TCF25 promotes ATP6V1A lysosomal delivery, the precise mechanism, including its role in V-ATPase assembly, remains elusive. We propose that TCF25 modulates specific ATP6V1A PTMs to facilitate trafficking. Future studies will elucidate these mechanisms.

## RESOURCE AVAILABILITY

### Lead contact

Further information and requests for reagents may be directed to and will be fulfilled by the lead contact, Zhenyu Cai (drcaizhenyu@tongji.edu.cn).

### Materials availability

This study did not generate new unique reagents.

### Data and code availability

- All data reported in this paper will be shared by the lead contact upon request.
- This paper does not report original code.
- Any additional information required to reanalyze the data reported in this paper is available from the lead contact upon request.

## STAR★METHODS

### EXPERIMENTAL MODEL AND STUDY PARTICIPANT DETAILS

#### Animals

Wild-type C57BL/6J mice were purchased from the Gempharmatech. TCF25^−/−^ mice were from BRL Medicine Inc. Mice were housed using 12 h light/dark cycles, with *ad libitum* access to food and water. They were routinely maintained on a standard chow diet (10.88 kJ/g; 8% fat, 21% protein, and 71% carbohydrate). Male mice at 8–12 weeks were used in all experiments. All animal care and experimental procedures complied with the National Institutes of Health guidelines and were approved by the animal care and use committee of Tongji University.

#### Cells

Wild-type and TCF25^−/−^ mouse dermal fibroblast (MDF), mouse lung fibroblasts (MIF), adipocytes and bone marrow-derived macrophages (BMDM) cells were isolated from C57BL/6J background mouse. Commercial cells were obtained from the American Type Culture Collection (ATCC, Manassas, VA). Primary mouse cells, HEK293T, HeLa, HT-29, A549 and SKOV3 cells were routinely maintained in DMEM with 10% FBS (v/v), 2mM of L-glutamine and 100U/mL of penicillin/streptomycin. 786-O, HCT8 and DLD1 cells were cultured in RPMI-1640 medium containing 10% FBS (v/v), 2mM of L-glutamine and 100U/mL of penicillin/streptomycin. Cells were grown at 37°C in a humidified atmosphere with 5% CO_2_ and were harvested in all experiments from exponentially growing cultures.

### METHOD DETAILS

#### Plasmids and lentiviral particles

The mammalian cell expression plasmids of FLAG-tagged mouse TCF25, FLAG-tagged human TCF25, mCherry-tagged human TCF25, mCherry-tagged mouse TCF25, HA-tagged human ATP6V1A and MYC-tagged human ATP6V0D1 were purchased from Synbio Technologies (Suzhou, China). PCDH-EF1-GAL3-GFP was purchased from TSINGKE. RFP-GFP-LC3 (Cat#LVCON360) was purchased from Genechem. pLJM1-Tmem192-mRFP-3xHA was a gift from Roberto Zoncu (Addgene plasmid # 134631).

To generate knockout cell lines with CRISPR-Cas9 approach, gRNA oligos were firstly cloned into pLenti-CRISPRv2 vector. To generate knockdown cell lines, shRNA oligos were firstly cloned into pLKO.1 vector. The gRNA/shRNA oligo sequences for individual gene are provided in Table S2. To generate lentivirus, HEK293T cells were co-transfected with pSPAX2 and pMD2G with/out sgRNA/shRNA targeted plasmids. After 24 h, supernatant was collected and used to infect cells. After 24 h of infection, cells were selected with puromycin for a further 48 h.

#### Cell culture and transfection

All the cell lines used in this study were obtained from the American Type Culture Collection (ATCC, Manassasa, VA). Wild-type (WT) and TCF25-deficient (TCF25^−/−^) mouse dermal fibroblast (MDF) cells were isolated from postnatal day 2 of C57BL/6 mouse skin. All cells were maintained in DMEM culture media supplemented with 10% FBS (v/v), 2 mM of L-glutamine and 100 U/mL of penicillin/streptomycin. Cells were grown at 37°C in a humidified atmosphere with 5% CO_2_ and were harvested in all experiments from exponentially growing cultures.

The plasmids were transfected with Lipo293F transfection reagent (Beyotime, China, Cat#C0518) in HEK293T cells according to the manufacturer’s protocol. MDF and HeLa cells were transfected with Lipo8000 transfection reagent (Beyotime, China, Cat#C0533) according to the manufacturer’s protocol. After 24 h, the cell lysates were analyzed by immunofluorescence or immunoprecipitation.

#### Genome-wide CRISPR screen and data analysis

The screen was performed by using murine genome-wide lentiviral sgRNA library (Addgene, Cat#67988), which comprising 90,230 sgRNAs targeting 18,424 genes (about 5 sgRNAs per gene). The genome-wide gRNA lentiviral particle was used to infect MDF cells stably expressing Cas9 at a multiplicity of infection (MOI) of 0.3. Cells were then selected with 1 μg/mL puromycin for 7 days. Then, a total of 3.2×10^7^ cells (about 300-fold library coverage) was treated with either normal medium or glucose-free medium for 24 h. After two cycles of starvation, cells were replaced with normal medium to recover and then approximately 1×10^8^ cells were harvested. Genomic DNA was extracted using the QIAamp DNA Blood Maxi Kit (QIAGEN, Cat#51194) according to the manufacturer’s instructions. sgRNA sequences were amplified by PCR followed by Next Generation Sequencing.

MAGeCK-VISPR50 was used for data analysis, including read mapping, sgRNA annotation, and comparison of the conditions with MAGeCK RRA algorithm.^20^ sgRNAs with fewer than 20 reads in normal medium treated group were omitted from the RRA algorithm analyses. Enriched genes with a cut-off P-value <0.05 were selected for further analysis. In volcano plot, at least 3 out of 5 different sgRNAs were enriched for the candidate gene. The enriched genes were listed in Table S1. Gene ontology (GO) enrichment analysis were performed using Metascape (https://metascape.org/gp/index.html).

#### Cell treatment and cell death assays

Cells were starved without glucose, amino acid or glutamine in DMEM with 10% dialyzed FBS to induced cell death. To induce TNF-dependent apoptosis, cells were treated with TNF-α (20 ng/mL) and Smac mimetic (10 nM) (TS). To induce TNF-dependent necroptosis, cells were pre-treated with z-VAD-fmk (20 μM) and Smac mimetic (10 nM) for 30 min followed by stimulation with TNF-α (20 ng/mL) (TSZ). To induce BAY-876-dependent cell death, cells were treated with BAY-876 (5 μM). To examined the protective effects of different compounds on glucose starvation-induced cell death, MDFs were starved without glucose for 16 h and then added CA-074me (2 μM), Nitroxoline (5 μM), Trolox (50 μM), DFO (200 μM), DFX (20 μM) for additional 8 h, respectively. If there are any differences, a detailed description will be given in the figure legend.

Cell death was examined by propidium iodide (PI) staining. Briefly, cells were trypsinized, collected by centrifugation, washed once with PBS buffer and then resuspended in PBS containing 5 μg/mL of PI. The stained cells were subjected to flow cytometry using a BD FACS Aria II (BD Biosciences, USA). The proportions of PI-positive cells were quantified with FlowJo Software (BD Biosciences, USA).

#### Cell fractionation and lysosome enrichment

The nuclei and cytoplasm fractionation were performed using Nuclear and Cytoplasmic Protein Extraction Kit (Beyotime, China, Cat#P0027) according to the manufacturer’s instructions. Briefly, 3×10^6^ cells were plated into 10-cm plates. After treatment, cells were placed on ice and washed twice with ice-cold PBS. Cells were scraped with 200 μL cytosol extraction buffer A and Vortex for 5 s, followed by adding 10 μL cytosol extraction buffer B. Next, cell lysates were centrifuged at 14000 g for 5 min and the supernatant was collected as cytosolic fraction. The pellet was resuspended with 50 μL nuclei extraction buffer followed by centrifuging at 14000 g for 10 min. The supernatant was collected as nuclei fraction. Finally, the fractionated proteins were subjected to immunoblotting using the specified antibodies.

Lysosomes were purified from HeLa cells with Optiprep density gradient media using the Lysosome Enrichment Kit (Thermofisher, USA, Cat#89839) according to manufacturer’s instructions. Briefly, 100 mg freshly cells were homogenized in Lysosome Enrichment Regent A and centrifuged for 500 g for 10 min. The supernatant was adjusted to 15% Optiprep and layered in a multistep Optiprep gradient consisting of 30, 27, 23, 20 and 17% Optiprep according to the manufacturer’s protocol and centrifuged for 2 h at 145,000 g in a swing out rotor. The various layers were formed at the junction of each gradient and collected after centrifugation. The enrichment of lysosomes in a specific fraction was then confirmed by immunoblotting with lysosomal marker LAMP1 antibody. The lysosome-enriched fraction was used to detect TCF25 expression by immunoblotting.

#### Membrane and cytoplasmic protein extraction

Membrane and cytoplasmic protein extraction were performed by using a Membrane and Cytoplasmic Protein Extraction Kit (Beyotime, China). Briefly, 1 × 10^7^ cells were resuspended in 500 μL of cytoplasmic protein isolation solution A and homogenized on ice. The cells lysis was first centrifuged at 700 × g for 10 min at 4°C to remove cellular debris. Subsequently, the supernatant was subjected to high-speed centrifugation at 14,000 × g for 30 min at 4°C. The resulting supernatant was the cytoplasmic protein fraction. The pellet was resuspended in 100 μL membrane protein isolation solution, homogenized on ice and centrifuged at 14 000 × g for 5 min. The resulting supernatant was the membrane protein fraction. Finally, the fractionated proteins were subjected to immunoblotting using the specified antibodies.

#### Immunoblotting and immunoprecipitation assays

Cell lysates were prepared for immunoblotting analysis using RIPA buffer supplemented with protease/phosphatase inhibitors and PMSF (MCE, Shanghai, China). The RIPA buffer consisted of 10 mM Tris-HCl (pH 8.0), 1 mM EDTA, 0.5 mM EGTA, 140 mM NaCl, 0.1% SDS, 1% Triton X-100, 50 mM NaF, 40 mM glycerophosphate, and 0.1 mM sodium vanadate. Cell lysates were separated by SDS-PAGE and analyzed by immunoblotting. The dilution of the antibodies used for western blotting is 1:1000. The proteins were visualized by enhanced chemiluminescence according to the manufacturer’s instructions (Tanon, Shanghai, China).

Cells were scraped and lysed in IP buffer (Beyotime, China) with protease/phosphatase inhibitors. After sonication for 10 s, the cell lysates were centrifuged for 10 min at 12,000 rpm. After centrifugation, the cell lysates were precipitated with antibody (1 μg) and protein A/G-magnetic beads (MCE, China, Cat# HY-K0202) by incubation at 4°C overnight. Following extensive washing with lysis buffer, the proteins bound to magnetic beads were eluted by boiling in 1×SDS sample buffer (30 μL). Finally, the precipitated proteins were subjected to immunoblotting using the specified antibodies. ImageJ (NIH, USA) software was used for greyscale analysis of western blotting.

#### Confocal imaging

To examine the localization of TCF25, cells were stably transfected with pLVX-mCherry-TCF25. The cells were seeded in confocal dishes and incubated at 37°C for 24 h. On the next day, cells were starved without glucose. Then, LysoTracker green (Beyotime, China) or Mito-Tracker Green (Beyotime, China) was added with a working concentration of 100 nM followed by capture with Nikon confocal microscopy. Measurement of co-localization was conducted using thresholded images using the image calculator function in ImageJ. Overlapping pixels in the red and green channels were measured and the percentage overlap was calculated from a minimum of ten fields per condition. The results shown are means ± SD from three independent experiments.

To stain the cells with LysoSensor, cells were seeded in confocal dishes and incubated at 37°C for 24 h. On the next day, cells were starved without glucose. LysoSensor (Yeasen, China) fluorescent dye (1 μM) was incubated with cells for 30 min at 37°C, followed by washing three times with FBS-free DMEM media. At least five representative images were captured by laser scanning confocal microscopy. The intensity of fluorescent spots was statistically analyzed by ImageJ software. A minimum of 25 cells were analyzed for each experiment. Results are presented as means ± SD of three independent experiments.

#### Cathepsin B activity assay

Cells were seeded on glass coverslips overnight and then incubated with normal medium or glucose-free medium for indicated time points. Then, the culture medium was replaced with 480 μL of fresh culture medium supplemented with 20 μL of 25×Cathepsin B Magic Red (MR) substrates (Abcam, USA, Cat#ab270772) for 30 min in the dark. Subsequently, cells were washed with medium twice. After wash, lysosomes were labeled with LysoTracker green and imaged immediately on a Nikon confocal microscope. All slides were imaged with the same laser setting. Cathepsin B MR intensity was determined by ImageJ software (NIH, USA). A minimum of 25 cells were analyzed for each experiment. Results are presented as means ± SD of three independent experiments.

#### Dextran degradation assay

Cells were seeded on glass coverslips and incubated in 0.4 mg/mL AlexaFluor 488-dextran (10 kDa) (Thermofisher, USA, Cat#D22910) for 20 min. The cells were then washed three times with dextran-free medium and incubated with glucose-free medium for indicated time points. Images were acquired using a Nikon confocal microscope. Fluorescence intensities were determined by ImageJ software (NIH, USA). A minimum of 25 cells were analyzed for each experiment. Results are presented as means ± SD of three independent experiments.

#### Intracellular ATP assay

Intracellular ATP was measured using the CellTiter-Lumi Assay kit (Beyotime, China, Cat#C0065) according to the manufacturer’s instructions. Briefly, cells were seeded in a 96-well plate (5,000 cells per well) one day before measurement. On the next day, 100 μL of premixed CellTiter-Glo reagent was added to each well after treatment and then the plate was incubated for 10 min at room temperature on an orbital shaker. After incubation, we recorded the luminescence using a Tecan Spark microplate reader (Tecan Instruments, Switzerland). The relative intracellular ATP level per cell was determined by dividing the total amount of luminescence by cell number.

#### Proximity ligation assay

The Proximity Ligation Assay (PLA) was performed using the Duolink *In Situ* Red Starter Kit (Sigma, USA, Cat#DUO92101) according to the manufacturer’s instructions. Briefly, cells were cultured on confocal culture dishes and then fixed in 4% paraformaldehyde (PFA) for 15 min, washed three times, permeabilized with 0.1% Triton X-100 for 5 min, and then blocked with Duolink blocking solution for 1 h at 37°C. Cells then incubated overnight at 4°C with primary antibodies diluted in Duolink antibody diluent. Subsequently, secondary antibodies were applied, followed by ligation and signal amplification steps according to the manufacturer’s instructions. The PLA dots were counted and standardized. A minimum of 25 cells were analyzed for each experiment. Results are presented as means ± SD of three independent experiments.

#### Lysosomal pH measurements

Lysosomal pH was estimated based on the fluorescence intensity ratio of pH-sensitive FITC (Thermofisher, USA, Cat#D-7172) and pH-insensitive TMR (Thermofisher, USA, Cat#D-1819) essentially.^54^ In brief, cells were loaded with 2.5 mg/mL 70kDa dextran coupled to FITC and TMR in complete medium for 18 h, washed with DPBS, and incubated for additional 12 h with complete medium or glucose-free medium without dextran. Images of dextran-loaded cells were acquired by a Nikon confocal microscope. and the FITC/TMR ratio was calculated by ImageJ software. Standard curves used to estimate lysosomal pH were created by similar analysis of cells incubated with a series of HEPESHepes buffers (145 mM KCl, 10 mM glucose, 1 mM MgCl_2_, 10 μM nigericin, 20 mM HEPESHepes) with pH ranging from 4.5 to 7.0. The PH standard curve created by Intracellular pH Calibration Buffer Kit (Invitrogen, USA, P35379). After transient transfections, pH changes were expressed as arbitrary units based on FITC/TMR ratio, the lower ratio indicating higher pH. A minimum of 25 cells were analyzed for each experiment. Results are presented as means ± SD of three independent experiments.

#### Total reactive oxygen species (ROS) assay

A total of 2 × 10^5^ MDF cells were seeded in six-well culture plate and incubated at 37°C for 16 h. On the next day, cells were incubated with glucose-free medium for indicated time points. Cells were then incubated with H2DCFDA (10 μM) at 37°C for 30 min and washed three times with PBS to remove unbound probes. The cells were collected for flow cytometry with FL1 channel and the data was analyzed by FlowJo Software.

#### Intracellular ferrous iron measurement

A total of 2×10^5^ MDF cells were seeded in six-well culture plate and incubated at 37°C for 24 h. On the next day, cells were incubated with glucose-free medium for indicated time points. Cells were washed three times with HBSS, stained with FerroOrange (1 μM) at 37°C for 30 min. The cells were collected for flow cytometry with FL1 channel and the data was analyzed by FlowJo Software.

#### V-ATPase assay

To purify lysosome, WT and TCF25^−/−^ MDF cells were stably transfected with lysosomal localized protein TMEM192-RFP-3×HA to perform Lyso-IP.^53^ Briefly, the cells were seeded in a 15-cm plate at a density appropriate for them to reach confluency after 24 h. The medium was removed, the cell monolayers were rinsed with ice-cold KPBS buffer (136 mM KCl and 10 mM KH_2_PO_4_, pH 7.25, supplemented with Pierce protease inhibitor tablets), scraped into 10 mL KPBS and collected by centrifugation at 1,500 rpm for 5 min. The pelleted cells were resuspended in a total volume of 1 mL KPBS (supplemented with 50 mM sucrose) and fractionated via Dounce homogenization, followed by centrifugation at 2,700 rpm. for 10 min. After determining the protein concentration, the post-nuclear supernatant was harvested. The supernatant containing 150 μg protein was precleared by rotation with 12 μL magnetic bead-conjugated rabbit IgG for 1 h at 4°C. The precleared supernatant aspirated on magnetic stand was then rotated with 20 μL Anti-HA Magnetic Beads (Thermofisher, USA, Cat #88836) for 2 h at 4°C and the supernatant was aspirated on magnetic stand. The lysosome-bound beads were washed twice with KPBS. The beads were suspended in 50 μL of V-ATPase reaction buffer (40 mM HEPES, pH 7.5, 1 mM MgCl_2_, 100 mM KCl, 0.25 mM Sucrose, 5 μg/mL Oligomycin, 2mM Ouabain,1mM DTT) in the presence or absence of 1 μM Concanamycin A supplemented with 1×protease inhibitor cocktail. The reaction was started by adding 1 mM ATP (final concentration) and incubating the samples for 30 min at 30°C. The amount of ATP hydrolyzed was detected using ADP-Glo Kinase Assay (Promega, USA, V6930) according to the manufacturer’s instruction.

#### Transmission electron microscopy (TEM) and lysosomal size measurements

Cells were fixed by adding the fixative solution (1.25% formaldehyde, 2.5% glutaraldehyde, and 0.03% picric acid in 0.1 M sodium cacodylate buffer, pH 7.4) directly to the culture medium at 1:1 ratio and incubate at room temperature for 1 h. After three washes with sodium cacodylate trihydrate 0.1 M of 15 min each, samples were incubated for 2 h with osmium tetroxide 1% in sodium cacodylate trihydrate 0.1 M at room temperature. The cells were washed three times for 15 min with sodium cacodylate trihydrate 0.1 M. Then, the cells were processed through dehydration with ethanol 30%–100% gradually. The cells were embedded into resin epoxy. After sample orientation, polymerization occurred at 60°C for 48 h. Samples were cut using ultramicrotome EM UC6 Leica, first in semithin of 250 nm and then ultrathin of 70 nm. Transmission electron microscopy imaging was done using a JEM-1230 (JEOL) Transmission electron microscopy. The lysosomal diameter was calculated using electron microscopy by drawing a line across the lysosomes and calculating each diameter according to the length of the scale bar, in microns. A total of 30 cells from three independent experiments were included to count the lysosome number and size.

#### Hepatic ischemia and reperfusion injury (IRI) model

6–8 weeks old male mice were anesthetized and the skin was cleaned by swabbing with a 75% ethanol and betadine solution. A ligation was performed around the portal vein and hepatic artery just above branching to the right lateral lobe. The animals were then subjected to a 60 min ischemic period at which point, the ligation was removed, and the occurrence of liver reperfusion was confirmed visually. Eighteen-four hours after reperfusion, mice were sacrificed, and the liver and blood were collected for analysis.

#### ALT, AST measurement

To determine the liver injury at the enzymatic level, the serum in blood samples from mice was separated through centrifugation at 3000 rpm for 20 min. The levels of liver enzymes, including oxaloacetic transaminase (AST) and alanine transaminase (ALT) was measured with Aspartate aminotransferase Assay Kit (Nanjing Jiancheng Bioengineering Institute, Catalog No: C010–2-1) and Alanine aminotransferase Assay Kit (Nanjing Jiancheng Bioengineering Institute, Catalog No: C009–2-1) according to the manufacturer’s instruction.

#### H&E staining assay

H&E staining was performed following established protocols. The livers were fixed in formalin overnight and subsequently embedded in paraffin blocks for sectioning. The liver sections were then subjected to haematoxylin and eosin staining, which facilitated the visualization of the nucleus and cytoplasm, respectively. The pathological images of livers were captured using an upright microscope. Semi quantitative analysis of liver tissue structural damage was performed by Suzuki score.^55^

#### TUNEL assay

TUNEL staining was employed to identify apoptotic cells, involving incubation with TUNEL antibody and subsequent staining of the nucleus with haematoxylin. The number of TUNEL-positive cells in the pathological images was quantified.

### QUANTIFICATION AND STATISTICAL ANALYSIS

The student’s t-test and one-way analysis of variance (ANOVA) were used for comparison among all different groups represented with the mean values ± standard errors. All experiments, except animal experiments, were independently repeated at least five times with similar results. Statistical analyses were performed with GraphPad Prism Software (RRID:SCR_002798). *p* value < 0.05 was considered statistically significant.

## Supplementary Material

1

2

3

Supplemental information can be found online at https://doi.org/10.1016/j.celrep.2025.116186.

## Figures and Tables

**Table:** KEY RESOURCES TABLE

REAGENT or RESOURCE	SOURCE	IDENTIFIER
Antibodies		

anti-TCF25	ABclonal	Cat# A15140; RRID:AB_2762027
anti-ATP6V1A	Abcam	Cat# ab199326; RRID:AB_2802119
anti-ATP6V0D1	Abcam	Cat# ab56441; RRID:AB_940402
anti-CTSB	CST	Cat# 31718; RRID:AB_2687580
anti-CTSD	Proteintech	Cat# 21327–1-AP; RRID:AB_10733646
anti-Cleaved Caspase-3	CST	Cat# 9664; RRID:AB_2070042
anti-Caspase-3	ABclonal	Cat# A2156; RRID:AB_2862975
anti-phospho-MLKL	Abcam	Cat# ab196436; RRID:AB_2687465
anti-MLKL	ABclonal	Cat# A5579; RRID:AB_2766355
anti-FLAG	ABclonal	Cat# AE004; RRID:AB_2771921
anti-HA	ABclonal	Cat# AE008; RRID:AB_2770404
anti-*c*-Myc	SANTA CRUZ	Cat# sc-40; RRID:AB_627268
anti-LAMP1	CST	Cat# 3243; RRID:AB_2134478
anti-COX IV	ZEN-BIOSCIENCE	Cat# 200147; RRID:AB_2722715
anti-Lamin A/C	ZEN-BIOSCIENCE	Cat# 201015–5D12
anti-Tubulin	Abbkine	Cat# A01030
anti-LC3	CST	Cat# 12741; RRID:AB_2617131
anti-NCOA4	Santa Cruz	Cat# sc-373739; RRID:AB_10915585
anti-FTH1	HUABIO	Cat# ET1610–78; AB_2923223
anti-SLC7A11	CST	Cat# 12691; RRID:AB_2687474
anti-SLC3A2	ABclonal	Cat# A5702; RRID:AB_2766461
GAPDH	HUABIO	Cat# ET1601–4; RRID: AB_2923224
Anti-Rab7	CST	Cat# 9367T; RRID:AB_1904103

Chemicals, peptides, and recombinant proteins		

Bafilomycin A1	MedChemExpress	Cat#HY-100558
Smac mimetic	MedChemExpress	Cat#HY-15989
Z-VAD-FMK	MedChemExpress	Cat#HY-16658B
H2DCFDA	MedChemExpress	Cat#HY-D0940
Nitroxoline	MedChemExpress	Cat#HY-B1159
CA-074 methyl ester	MedChemExpress	Cat#HY-100350
BAY-876	MedChemExpress	Cat#HY-100017
Deferoxamine	MedChemExpress	Cat#HY-B1625
Deferasirox	MedChemExpress	Cat#HY-17359
Ouabain	MedChemExpress	Cat#HY-B0542
Concanamycin A	MedChemExpress	Cat#HY-N1724
Recombinant mouse TNF-α	R&D System	Cat#410-MT-025/CF
Lipo293F^™^ transfection reagent	Beyotime	Cat#C0518
Lipo8000^™^ transfection reagent	Beyotime	Cat#C0533
LysoTracker green	Beyotime	Cat#C1047S
Mito-Tracker Green	Beyotime	Cat#C1048
LysoSensor	Yeasen	Cat#40767ES50
FerroOrange	DOJINDO	Cat#F374
pH-sensitive FITC	Thermofisher	Cat#D-7172
pH-insensitive TMR	Thermofisher	Cat#D-1819
Pyruvate	BasalMedia	Cat#S410JV
Glucose-6-phosphate	BBI Life Sciences	Cat#620045
protein A/G-magnetic beads	MedChemExpress	Cat# HY-K0202
Cathepsin B Magic Red (MR) substrates	Abcam	Cat#ab270772
AlexaFluor 488-dextran (10 kDa)	Thermofisher	Cat#D22910

Critical commercial assays		

CellTiter-Lumi^™^ Assay kit	Beyotime	Cat#C0065
Nuclear and Cytoplasmic Protein Extraction Kit	Beyotime	Cat#P0027
Membrane and Cytosol Protein Extraction Kit	Beyotime	Cat#P0033
Duolink^®^ *In Situ* Red Starter Kit	Sigma	Cat#DUO92101
Lysosome Enrichment Kit	Thermofisher	Cat#89839
Intracellular pH Calibration Buffer Kit	Invitrogen	Cat#P35379
ADP-Glo Kinase Assay	Promega	Cat#V6930
Aspartate aminotransferase Assay Kit	Nanjing Jiancheng Bioengineering Institute	Cat#C010–2-1
Alanine aminotransferase Assay Kit	Nanjing Jiancheng Bioengineering Institute	Cat#C009–2-1

Experimental models: cell lines		

Mouse dermal fibroblast (MDF)	This paper	N/A
Mouse lung fibroblasts (MIF)	This paper	N/A
Adipocytes	This paper	N/A
Bone marrow-derived macrophages (BMDM)	This paper	N/A
Hela	ATCC	Cat#CRM-CCL-2
HEK293T	ATCC	Cat#CRL-11268
HT-29	ATCC	Cat#HTB-38
SKOV3	ATCC	Cat#HTB-77
786-O	ATCC	Cat#CRL-1932
HCT8	ATCC	Cat#CCL-244
DLD1	ATCC	Cat#CCL-221
A549	ATCC	Cat#CRM-CCL-185

Experimental models: organisms/strains		

C57BL/6J	Gempharmatech	N/A
TCF25^−/−^	BRL Medicine Inc	N/A

Recombinant DNA		

pLVX-HA-hsATP6V1A	This paper	N/A
pLVX-HA-hsATP6V0d1	This paper	N/A
pLVX-HA-musATP6V0d1	This paper	N/A
pLVX-FLAG-musTCF25	This paper	N/A
pLVX-mcherry-hsTCF25	This paper	N/A
pLVX-mcherry-musTCF25	This paper	N/A
PCMV3-FLAG-hsTCF25	This paper	N/A
PCDH-EF1-GAL3-GFP	This paper	N/A
RFP-GFP-LC3	This paper	N/A
pLJM1-Tmem192-mRFP-3xHA^53^	Addgene	Cat#134631

Software and algorithms		

FlowJo v10.07	FLOWJO	https://www.flowjo.com/learn/flowjouniversity/flowjo/getting-started-in-flowjo/131
GraphPad Prism 6	GraphPad	https://www.graphpad.com/scientificsoftware/prism/
ImageJ v1.8.0	ImageJ	https://imagej.nih.gov/ij

## Highlights

- A CRISPR-Cas9 screen identifies TCF25 as critical for glucose-starvation-induced cell death
- TCF25 enhances lysosomal acidification via V-ATPase during glucose deprivation
- TCF25-mediated ferritinophagy under glucose deprivation triggers lysosomal cell death
- TCF25 deficiency confers protection against hepatic ischemia-reperfusion injury in mice
