# Supplementary material for: TCF25 serves as a nutrient sensor to orchestrate metabolic adaptation and cell death by enhancing lysosomal acidification under glucose starvation
Source: Cell Rep. Author manuscript; Available in PMC 2026 Mar 24. (PMC13010378; doi:10.1016/j.celrep.2025.116186)
Supplement: 1 [file NIHMS2113379-supplement-1.pdf]

Cell Reports, Volume *44*

Supplemental information

TCF25 serves as a nutrient sensor to orchestrate metabolic adaptation and cell death by enhancing lysosomal acidification under glucose starvation

Wenqing Ren (任文青), Hui Jiang (江辉), Qianqian Song (宋倩倩), Yiliang Chen (陈义亮), Chenxiao Tang (唐晨晓), Fang Wang (王芳), Jing Zhu (朱静), Jingming Ren (任靓明), Yaxing Zhao (赵亚星), Yuan He (贺源), Jin Cai (蔡锦), Tianle Zhang (张天乐), Zhuhong Wang (汪竹红), Chenjie Zhu (朱晨杰), Wen Xue (薛闻), Ai Peng (彭艾), Xiaona Feng (冯小娜), Yue Liu (刘悦), Jianqiang Yu (余建强), Zheng-gang Liu (刘正刚), and Zhenyu Cai (蔡振宇)

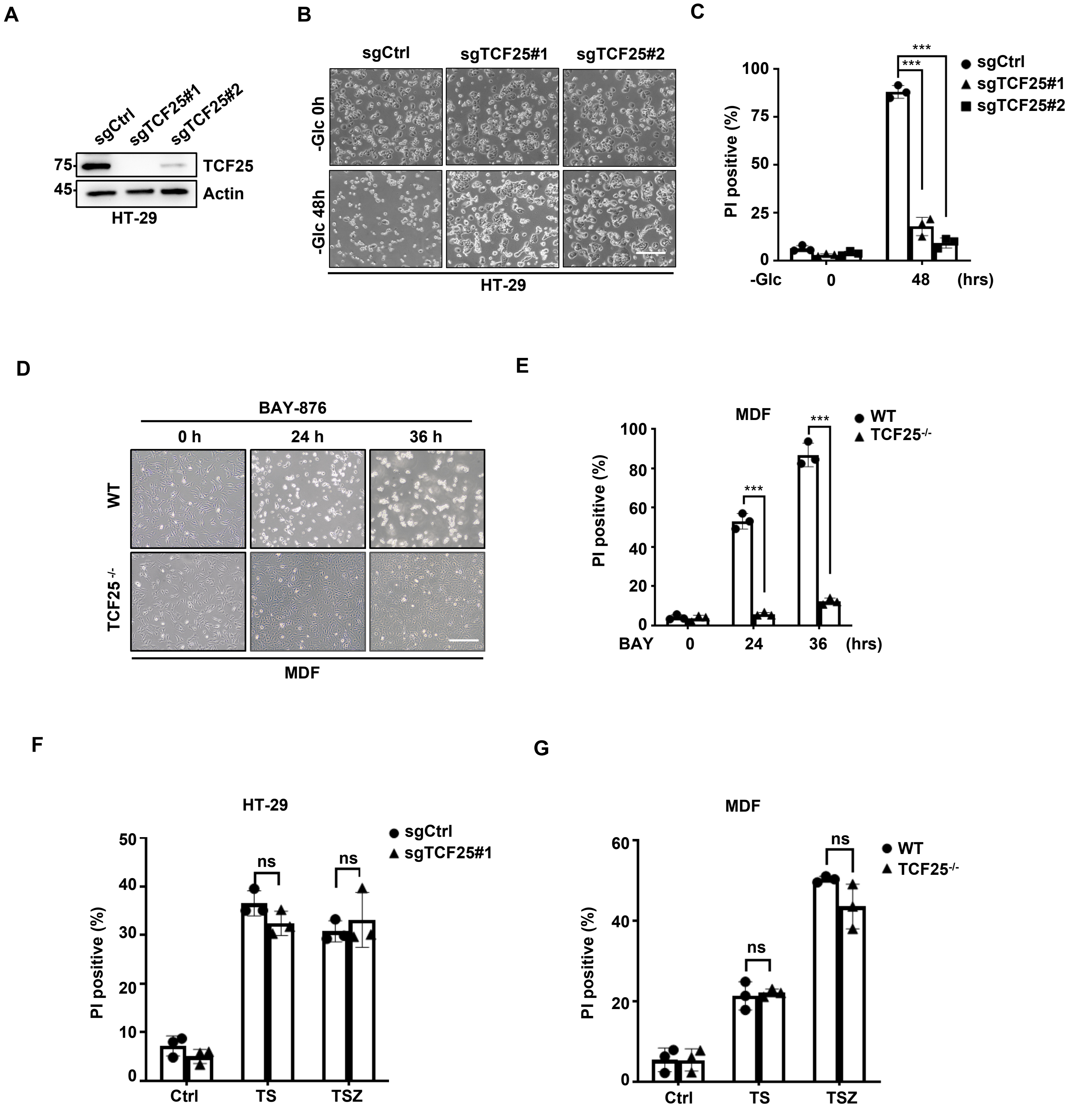
A
sgCtrl
sgTCF25#1
sgTCF25#2
75
45
TCF25
Actin
HT-29
B
sgCtrl
sgTCF25#1
sgTCF25#2
-Glc 0h
-Glc 48h
HT-29
C
PI positive (%)
100
75
50
25
0
-Glc
0
48
(hrs)
***
***
sgCtrl
sgTCF25#1
sgTCF25#2
D
BAY-876
0 h
24 h
36 h
WT
TCF25 -/-
MDF
E
MDF
WT
TCF25-/-
PI positive (%)
100
80
60
40
20
0
BAY
0
24
36
(hrs)
***
***
F
HT-29
sgCtrl
sgTCF25#1
PI positive (%)
50
40
30
20
10
0
ns
ns
Ctrl
TS
TSZ
G
MDF
WT
TCF25-/-
PI positive (%)
60
40
20
0
ns
ns
Ctrl
TS
TSZ

Figure S1

Fig. S1 Loss of TCF25 protects cells from glucose starvation-induced cell death but not TNF-α-induced apoptosis and necroptosis.

(A) HT-29 cells were stably transfected with sgRNA-Control (sgCtrl) and two individual sgRNAs targeting TCF25 (sgTCF25#1 and sgTCF25#2), respectively. TCF25 expression was examined by immunoblotting with its specific antibody. (B) sgCtrl, sgTCF25#1 and sgTCF25#2 HT-29 cells were starved without glucose for 48 hrs and the representative images were shown. Scale bar, 100 μm. (C) Cell death of the HT-29 cells in (B) was determined by PI staining. (D) WT and $TCF25^{-/-}$ MDFs were treated with BAY-876 (5μm) for indicated time points and the representative images were shown. Scale bar, 100 μm. (E) Cell death of the MDFs in (D) was determined by PI staining. (F) sgCtrl and sgTCF25#1 HT-29 cells were treated with TS (20 ng/ml TNF-α, 100 nM Smac mimetic) for 18 hrs to induce apoptosis or TSZ (20 ng/ml TNF-α, 10 nM Smac mimetic, 20 μM Z-VAD-FMK) for 6 hrs to induce necroptosis. Cell death of the HT-29 cells was determined by PI staining. (G) WT and $TCF25^{-/-}$ MDFs were treated with TS for 12 hrs to induce apoptosis or TSZ for 4 hrs to induce necroptosis. Cell death of the MDFs was determined by PI staining. Western data are representative of three independent experiments. Bar graphs represent the mean ± SD from three independent experiments. Statistical analysis was performed using a two-sided student's t-test. The levels of significance were indicated by ***, $P < 0.001$; ns. not significant. (Two-way ANOVA).

Figure S1

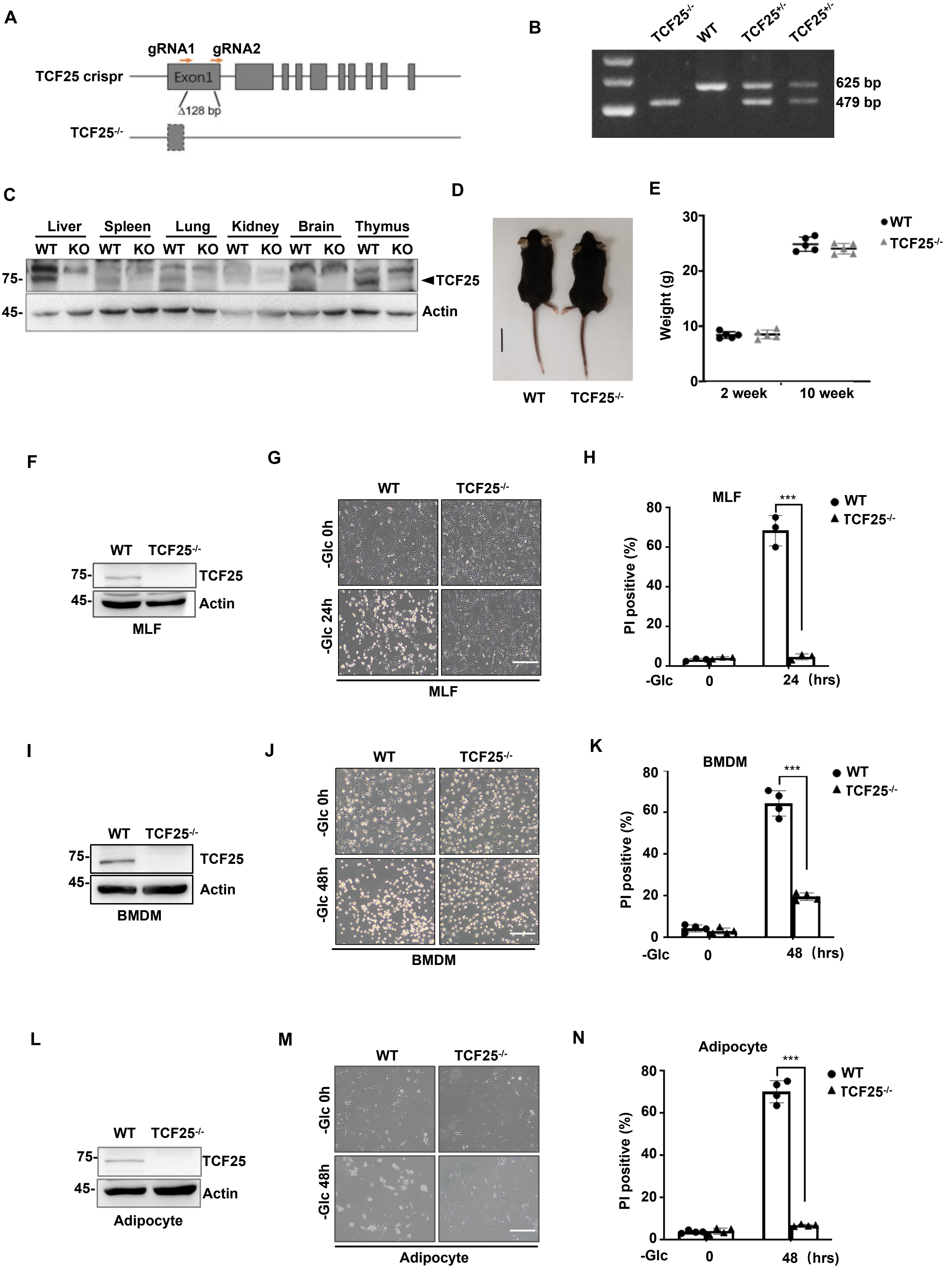
A
gRNA1
gRNA2
TCF25 crispr
Exon1
Δ128 bp
TCF25-/-
B
TCF25-/-
WT
TCF25+/-
TCF25+/-
625 bp
479 bp
C
Liver
Spleen
Lung
Kidney
Brain
Thymus
WT KO WT KO WT KO WT KO WT KO WT KO
75-
45-
◄TCF25
Actin
D
WT
TCF25-/-
E
Weight (g)
30
20
10
0
2 week
10 week
WT
TCF25-/-
F
WT
TCF25-/-
75-
45-
TCF25
Actin
MLF
G
WT
TCF25-/-
-Glc 0h
-Glc 24h
MLF
H
MLF
PI positive (%)
80
60
40
20
0
***
WT
TCF25-/-
-Glc
0
24 (hrs)
I
WT
TCF25-/-
75-
45-
TCF25
Actin
BMDM
J
WT
TCF25-/-
-Glc 0h
-Glc 48h
BMDM
K
BMDM
PI positive (%)
80
60
40
20
0
***
WT
TCF25-/-
-Glc
0
48 (hrs)
L
WT
TCF25-/-
75-
45-
TCF25
Actin
Adipocyte
M
WT
TCF25-/-
-Glc 0h
-Glc 48h
Adipocyte
N
Adipocyte
PI positive (%)
80
60
40
20
0
***
WT
TCF25-/-
-Glc
0
48 (hrs)

Figure S2

Fig. S2 Various types of the cells isolated from TCF25 knockout mouse are resistant to glucose starvation-induced cell death

(A) Schematic depiction of the TCF25 knockout mice generated by CRISPR/Cas9-mediated gene targeting strategy in C57BL/6N zygotes. TCF25 knockout mice were generated by targeting exons 1. An allele harboring a 128 bp deletion in exon 1 was chosen as a knockout allele. (B) Genotyping of WT, heterozygous (TCF25$^{+/-}$) and homozygous (TCF25$^{-/-}$) mice. (C) Immunoblotting analysis of TCF25 expression in different tissues from both WT and TCF25$^{-/-}$ mice. (D) Phenotype of WT and TCF25$^{-/-}$ mice. Scale bar, 2 cm. (E) Weight of WT and TCF25$^{-/-}$ male mice at the indicated ages. (F) Mouse lung fibroblasts (MLFs), (I) Bone marrow-derived macrophages (BMDMs) and (L) adipocytes were isolated from WT and TCF25$^{-/-}$ mice. The expression of TCF25 was determined by immunoblotting with its specific antibody, respectively. (G) WT and TCF25$^{-/-}$ MLFs, (J) BMDMs or (M) adipocytes were starved without glucose and the representative images were shown, respectively. Scale bar, 100 μm. (H) Cell death of the MLFs, (K) BMDMs and (N) adipocytes was determined by PI staining, respectively. All western data are representative of three independent experiments. Bar graphs represent the mean ± SD from three independent experiments. Statistical analysis was performed using a two-sided student's t-test. The levels of significance were indicated by ***, $P < 0.001$. (Two-way ANOVA).

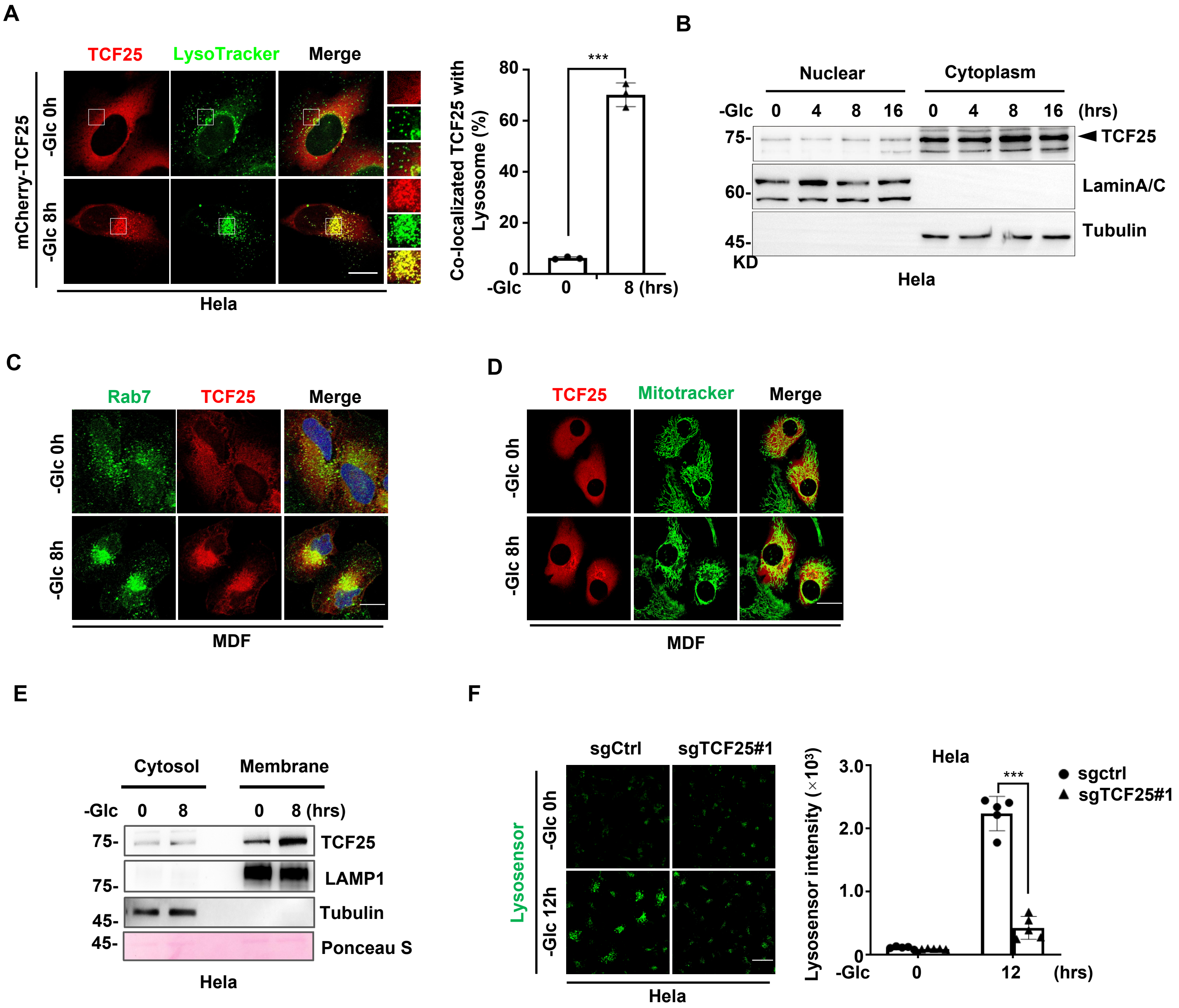
A
TCF25
LysoTracker
Merge
mCherry-TCF25
-Glc 0h
-Glc 8h
Hela
Co-localizated TCF25 with Lysosome (%)
***
-Glc
0
8 (hrs)
B
Nuclear
Cytoplasm
-Glc 0 4 8 16 0 4 8 16 (hrs)
75-
60-
45-
KD
TCF25
LaminA/C
Tubulin
Hela
C
Rab7
TCF25
Merge
-Glc 0h
-Glc 8h
MDF
D
TCF25
Mitotracker
Merge
-Glc 0h
-Glc 8h
MDF
E
Cytosol
Membrane
-Glc 0 8 0 8 (hrs)
TCF25
LAMP1
Tubulin
Ponceau S
Hela
F
sgCtrl
sgTCF25#1
Lysosensor
-Glc 0h
-Glc 12h
Hela
Lysosensor intensity (×10³)
sgctrl
sgTCF25#1
-Glc 0 12 (hrs)

Figure S3

Fig. S3 TCF25 localizes to lysosomes and enhances lysosomal acidification under glucose starvation, related to Fig. 2.

(A) Hela cells stably expressing mCherry-TCF25 were starved without glucose at the indicated time points and then stained with LysoTracker™ Green. Left, representative confocal images of the cells were shown. Right, statistical analysis of the co-localized mcherry-TCF25 with LysoTracker™ Green in Hela cells. Scale bar, 20 μm. (B) Hela cells were starved without glucose at the indicated time points and then fractionated into cytosolic (Cyto) and nuclear (Nuc) fractions. The fractions were then analyzed by immunoblotting with the indicated antibodies. (C) MDFs stably expressing mCherry-TCF25 were starved without glucose at the indicated time points and then stained with Rab7 antibody followed by immunofluorescence. Representative confocal images of the cells were shown. Scale bar, 20 μm. (D) MDFs stably expressing mCherry-TCF25 were starved without glucose at the indicated time points and then stained with MitoTracker™ Green. Representative confocal images of the cells were shown. Scale bar, 20 μm. (E) MDFs were starved without glucose at the indicated time points and then fractionated into cytosol and membrane fractions. The fractions were analyzed by immunoblotting with the indicated antibodies. (F) sgCtrl and sgTCF25#1 Hela cells were starved without glucose at the indicated time points and then stained with LysoSensor™. Left, representative confocal images of the cells were shown. Scale bar, 20 μm. Right, statistical analysis of the LysoSensor™ fluorescence intensity. Western data are representative of three independent experiments. Bar graphs represent the mean ± SD from three independent experiments. Statistical analysis was performed using a two-sided student's t-test. The levels of significance were indicated by ***, $P < 0.001$. (Two-way ANOVA).

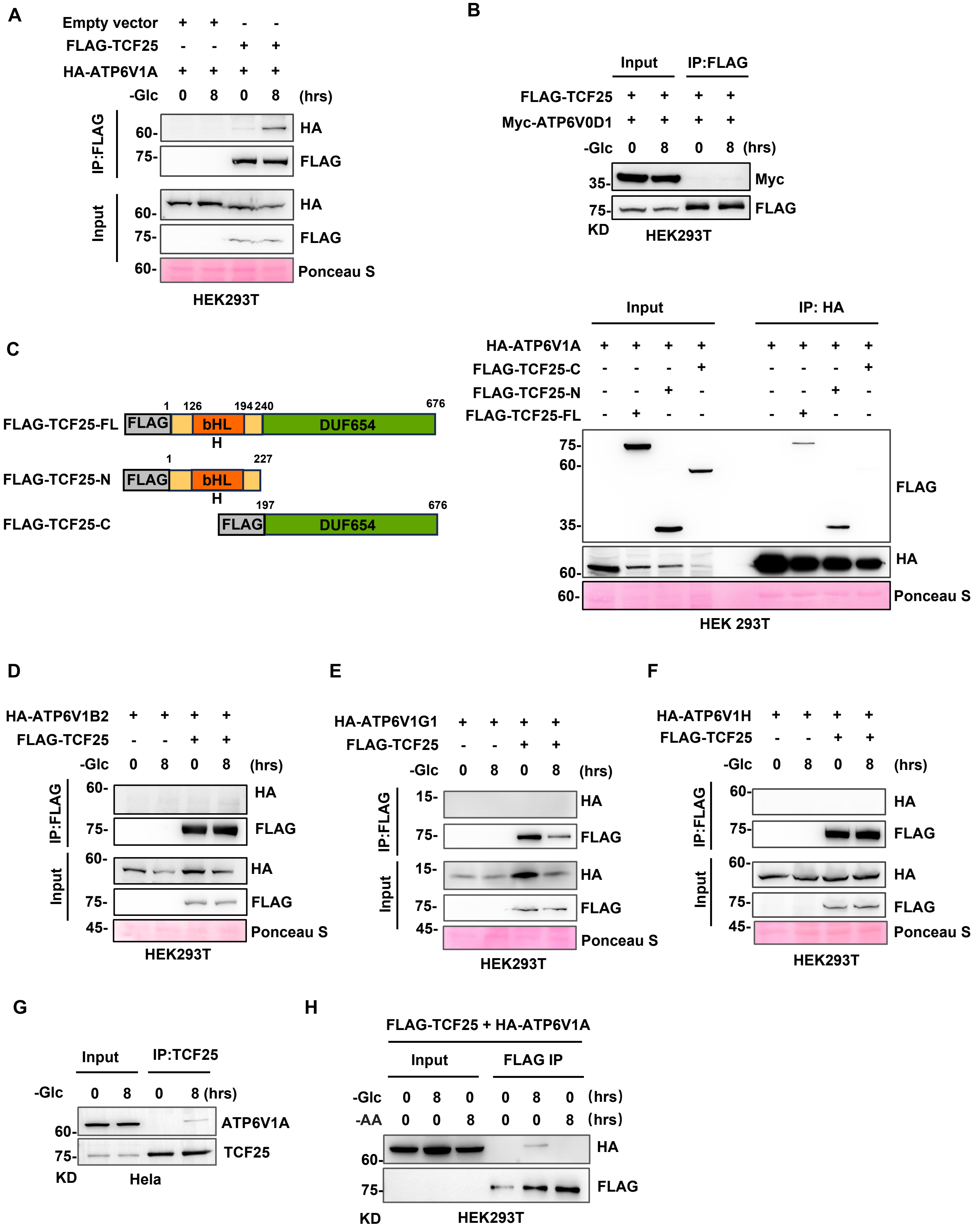
A
Empty vector + + - -
FLAG-TCF25 - - + +
HA-ATP6V1A + + + +
-Glc 0 8 0 8 (hrs)
IP:FLAG
Input
60-
75-
HA
FLAG
Ponceau S
HEK293T
B
Input
IP:FLAG
FLAG-TCF25 + + + +
Myc-ATP6V0D1 + + + +
-Glc 0 8 0 8 (hrs)
35-
75-
KD
Myc
FLAG
HEK293T
C
FLAG-TCF25-FL
FLAG-TCF25-N
FLAG-TCF25-C
FLAG
bHL
H
DUF654
1 126 194 240 676
1 227
197 676
Input
IP: HA
HA-ATP6V1A + + + + + + + +
FLAG-TCF25-C - - - + - - - +
FLAG-TCF25-N - - + - - - + -
FLAG-TCF25-FL - + - - - + - -
75-
60-
35-
FLAG
HA
Ponceau S
HEK 293T
D
HA-ATP6V1B2 + + + +
FLAG-TCF25 - - + +
-Glc 0 8 0 8 (hrs)
IP:FLAG
Input
45-
HEK293T
E
HA-ATP6V1G1 + + + +
FLAG-TCF25 - - + +
-Glc 0 8 0 8 (hrs)
15-
F
HA-ATP6V1H + + + +
FLAG-TCF25 - - + +
-Glc 0 8 0 8 (hrs)
G
Input
IP:TCF25
-Glc 0 8 0 8 (hrs)
ATP6V1A
TCF25
Hela
H
FLAG-TCF25 + HA-ATP6V1A
Input
FLAG IP
-Glc 0 8 0 0 8 0 (hrs)
-AA 0 0 8 0 0 8 (hrs)

Figure S4

Fig. S4 TCF25 interacts with ATP6V1A in response to glucose starvation, related to Fig. 3

(A) HEK293T cells were co-transfected with FLAG-TCF25 and HA-ATP6V1A or empty vector as indicated. The cells were then starved without glucose at the indicated time points. Cell lysates were immunoprecipitated with HA antibody (IP:HA) and analyzed by immunoblotting with the indicated antibodies. Ponceau S staining was used as a loading control. (B) HEK293T cells were co-transfected with FLAG-TCF25 and Myc-ATP6V0D1. The cells were starved without glucose at the indicated time points. Cell lysates were immunoprecipitated with FLAG antibody (IP:FLAG) and analyzed by immunoblotting with indicated antibodies. (C) Left, schematic representation of full-length and truncated TCF25. Right, HEK293T cells were co-transfected with HA-ATP6V1A and FLAG-TCF25-FL, FLAG-TCF25-N or FLAG-TCF25-C as indicated and then starved without glucose for 8 hrs. Cell lysates were immunoprecipitated with HA antibody (IP:HA) and analyzed by immunoblotting with the indicated antibodies. Ponceau S staining was used as a loading control. (D) HEK293T cells were transfected with FLAG-TCF25 and HA-ATP6V1B2 or (E) HA-ATP6V1G1, or (F) HA-ATP6V1H as indicated. The cells then starved without glucose at the indicated time points. Cell lysates were immunoprecipitated with HA antibody (IP:HA) and analyzed by immunoblotting with the indicated antibodies. Ponceau S staining was used as a loading control. (G) Hela cells were starved without glucose at the indicated time points. Cell lysates were immunoprecipitated with TCF25 antibody (IP:TCF25) and analyzed by immunoblotting with indicated antibodies. (H) HEK293T cells were co-transfected with FLAG-TCF25 and HA-ATP6V1A. The cells were then starved without glucose or amino acids (AA) at the indicated time points. Cell lysates were immunoprecipitated with FLAG antibody (IP:FLAG) and analyzed by immunoblotting with indicated antibodies. All western data are representative of three independent experiments.

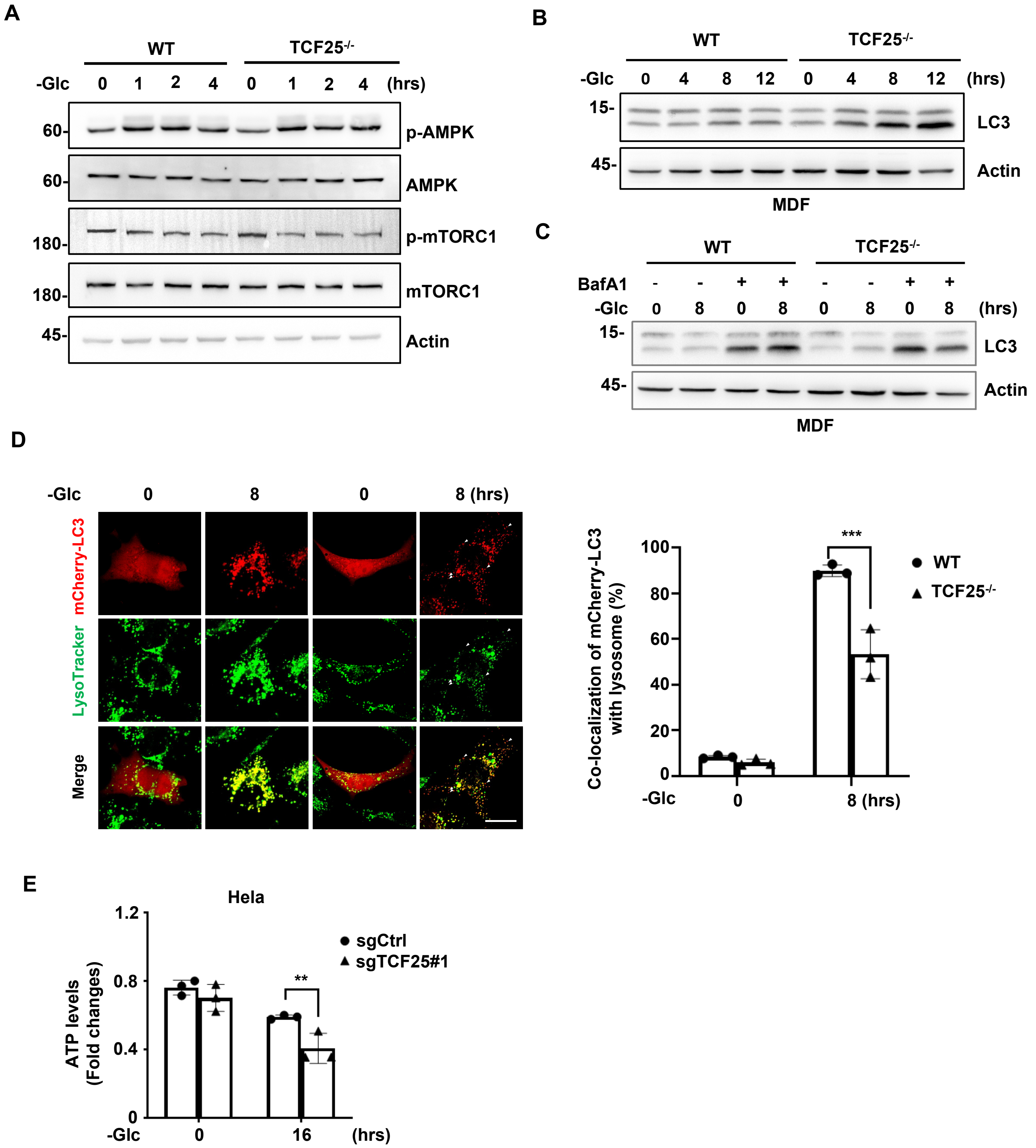
A
WT
TCF25-/-
-Glc 0 1 2 4 0 1 2 4 (hrs)
60-
p-AMPK
60-
AMPK
180-
p-mTORC1
180-
mTORC1
45-
Actin
B
WT
TCF25-/-
-Glc 0 4 8 12 0 4 8 12 (hrs)
15-
LC3
45-
Actin
MDF
C
WT
TCF25-/-
BafA1 - - + + - - + +
-Glc 0 8 0 8 0 8 0 8 (hrs)
15-
LC3
45-
Actin
MDF
D
-Glc 0 8 0 8 (hrs)
mCherry-LC3
LysoTracker
Merge
Co-localization of mCherry-LC3 with lysosome (%)
100
80
60
40
20
0
***
WT
TCF25-/-
-Glc 0 8 (hrs)
E
Hela
1.2
0.8
0.4
0
ATP levels (Fold changes)
**
sgCtrl
sgTCF25#1
-Glc 0 16 (hrs)

Figure S5

Figure S5 TCF25 does not modify the activation status of AMPK signaling but is crucial in maintaining cellular energy balance under glucose starvation.

(A) WT and TCF25$^{-/-}$ MDFs were starved without glucose at the indicated time points. Cell lysates were examined by immunoblotting with the indicated antibodies. Western data are representative of three independent experiments. (B) WT and TCF25$^{-/-}$ MDFs were starved without glucose at the indicated time points. Cells were lysed and immunoblotted with LC3 antibody. (C) WT and TCF25$^{-/-}$ MDFs were pre-treated with/out BafA1 (1 μM) for 30 min and then starved without glucose at the indicated time points. Cells were lysed and immunoblotted with LC3 antibody. (D) WT and TCF25$^{-/-}$ MDFs expressed mCherry-LC3 were starved without glucose at the indicated time points and stained with LysoTracker Green. Left, Representative confocal images of the cells were shown. White arrows indicate mCherry-LC3 puncta not co-localized with LysoTracker. Scale bar, 20 μm. Right, Statistical analysis of co-localized mCherry-LC3 puncta with lysosomes. (E) sgCtrl and sgTCF25#1 Hela cells reconstituted were starved without glucose at the indicated time points. The relative ATP levels were measured by ATP-Glo™ bioluminometric assay and normalized to cell number. Western data are representative of three independent experiments. Bar graphs represent the mean ± SD from three independent experiments. Statistical analysis was performed using a two-sided student's t-test. The levels of significance were indicated by ＊＊, P <0.01; n.s. not significant. (two-way ANOVA).

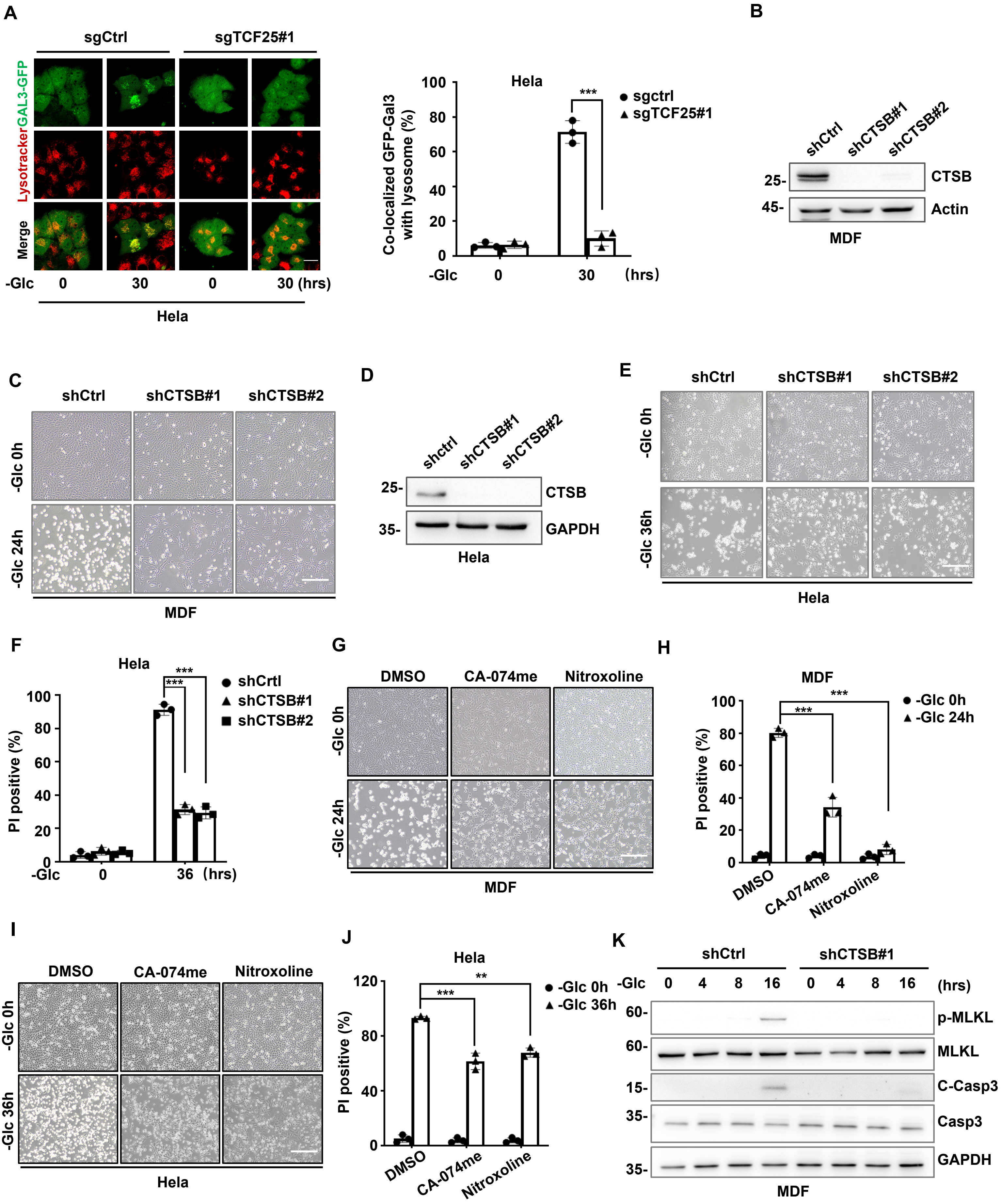
A
sgCtrl
sgTCF25#1
LysotrackerGAL3-GFP
Merge
-Glc 0 30 0 30 (hrs)
Hela
Co-localized GFP-Gal3 with lysosome (%)
Hela
sgctrl
sgTCF25#1
B
shCtrl
shCTSB#1
shCTSB#2
CTSB
Actin
MDF
C
-Glc 0h
-Glc 24h
D
shctrl
GAPDH
E
-Glc 36h
F
shCrtl
PI positive (%)
G
DMSO
CA-074me
Nitroxoline
H
-Glc 0h
-Glc 24h
I
J
K
p-MLKL
MLKL
C-Casp3
Casp3

Figure S6

Fig. S6 Sustained glucose starvation increases LMP to induce LDCD

(A) sgCtrl and sgTCF25#1 Hela cells were transfected with GFP-Gal3 and then starved without glucose at the indicated time points. The cells were stained with LysoTracker™ Red. Left, representative confocal images of the cells were shown. Right, statistical analysis of the co-localized GFP-Gal3 with LysoTracker-Red was shown. Scale bar, 20 μm. (B) MDFs were stably transfected with shRNA-Control (shCtrl) and two individual shRNAs targeting CTSB (shCTSB#1 and shCTSB#2), respectively. CTSB expression was examined by immunoblotting with its specific antibody. (C) shCtrl, shCTSB#1 and shCTSB#2 MDFs were starved without glucose for 24 hrs and the representative images were shown. Scale bar, 100 μm. (D) Hela cells were stably transfected with shRNA-Control (shCtrl) and two individual shRNAs targeting CTSB (shCTSB#1 and shCTSB#2), respectively. CTSB expression was examined by immunoblotting with its specific antibody. (E) shCtrl, shCTSB#1 and shCTSB#2 Hela cells were starved without glucose for 36 hrs and the representative images were shown. Scale bar, 100 μm. (F) Cell death of the Hela cells in (E) was determined by PI staining. (G) MDFs were firstly starved without glucose for 12 hrs and then supplied with CA-074me (1μ M) or Nitroxoline (5 μ M) for additional 12 hrs. The representative images were shown. Scale bar, 100 μm. (H) Cell death of the MDFs in (G) was determined by PI staining. (I) Hela cells were firstly starved without glucose for 24 hrs and then supplied with CA-074me (1μ M) or Nitroxoline (5 μ M) for 12 hrs. The representative images were shown. Scale bar, 50 μm. (J) Cell death of the MDFs in (I) was determined by PI staining. (K) shCtrl and shCTSB#1 MDFs were starved without glucose at the indicated time points. Cells were lysed and immunoblotted with the indicated antibodies. All western data are representative of three independent experiments. Bar graphs represent the mean ± SD from three independent experiments. Statistical analysis was performed using a two-sided student's t-test. The levels of significance were indicated by＊＊＊ P <0.001; n.s. not significant. (Two-way ANOVA).

Figure S6

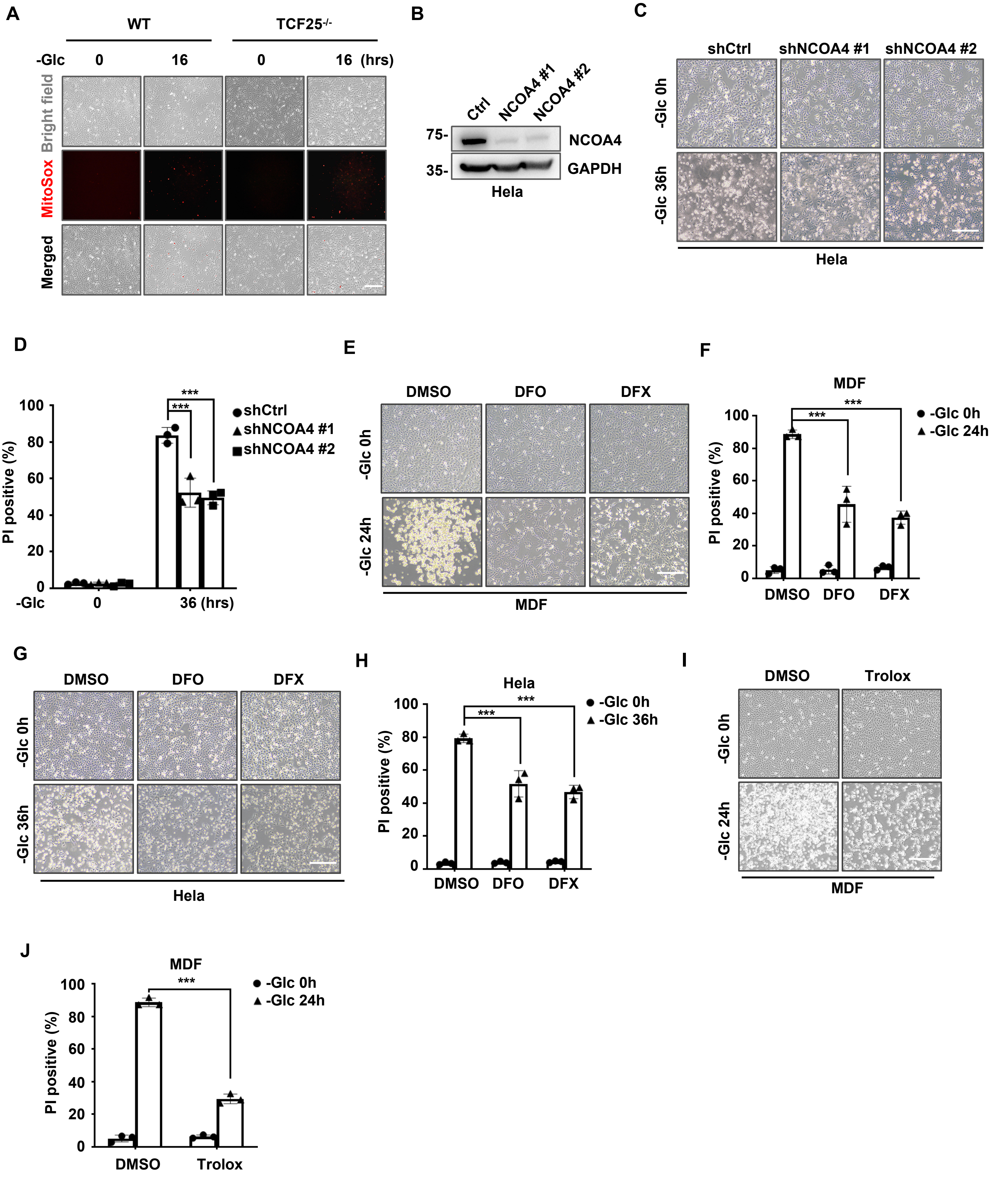
A
WT
TCF25-/-
-Glc 0 16 0 16 (hrs)
Bright field
MitoSox
Merged
B
Ctrl
NCOA4 #1
NCOA4 #2
75-
35-
NCOA4
GAPDH
Hela
C
shCtrl
shNCOA4 #1
shNCOA4 #2
-Glc 0h
-Glc 36h
Hela
D
PI positive (%)
shCtrl
shNCOA4 #1
shNCOA4 #2
***
-Glc 0 36 (hrs)
E
DMSO
DFO
DFX
-Glc 0h
-Glc 24h
MDF
F
MDF
-Glc 0h
-Glc 24h
PI positive (%)
DMSO DFO DFX
G
DMSO
DFO
DFX
-Glc 0h
-Glc 36h
Hela
H
Hela
-Glc 0h
-Glc 36h
PI positive (%)
DMSO DFO DFX
I
DMSO
Trolox
-Glc 0h
-Glc 24h
MDF
J
MDF
-Glc 0h
-Glc 24h
PI positive (%)
DMSO Trolox

Figure S7

Fig. S7 Redox-active iron contributes to cell death under prolonged glucose starvation. (A) WT and TCF25$^{-/-}$ MDFs were starved without glucose at the indicated time points. The cells were stained with mitochondria superoxide indicator MitoSOX Red and the representative images were shown. Scale bar, 100 μm. (B) Hela cells were stably transfected with shRNA-Control (shCtrl) and two individual shRNAs targeting NCOA4 (shNCOA4#1 and shNCOA4#2), respectively. NCOA4 expression was examined by immunoblotting with its specific antibody. (C) shCtrl, shNCOA4#1 and shNCOA4#2 Hela cells were starved without glucose for 36 hrs and the representative images were shown. Scale bar, 100 μm. (D) Cell death of the Hela cells in (C) was determined by PI staining. (E) MDFs were starved without glucose for 16 hours and then added DFO (200 μM) or DFX (20 μM) for additional 8 hours and the representative images were shown. Scale bar, 100 μm. (F) Cell death of the MDFs in (E) was determined by PI staining. (G) Hela cells were starved without glucose for 24 hours and then added DFO (200 μM) or DFX (20 μM) for additional 12 hours and the representative images were shown. Scale bar, 100 μm. (H) Cell death of the Hela cells in (G) was determined by PI staining. (I) MDFs were starved without glucose for 16 hours and then added Trolox (50 μM) for additional 8 hours and the representative images were shown. Scale bar, 100 μm. (J) Cell death of the MDFs in (I) was determined by PI staining. All western data are representative of three independent experiments. Bar graphs represent the mean ± SD from three independent experiments. Statistical analysis was performed using a two-sided student's t-test. The levels of significance were indicated by ＊＊＊ P <0.001. (Two-way ANOVA).

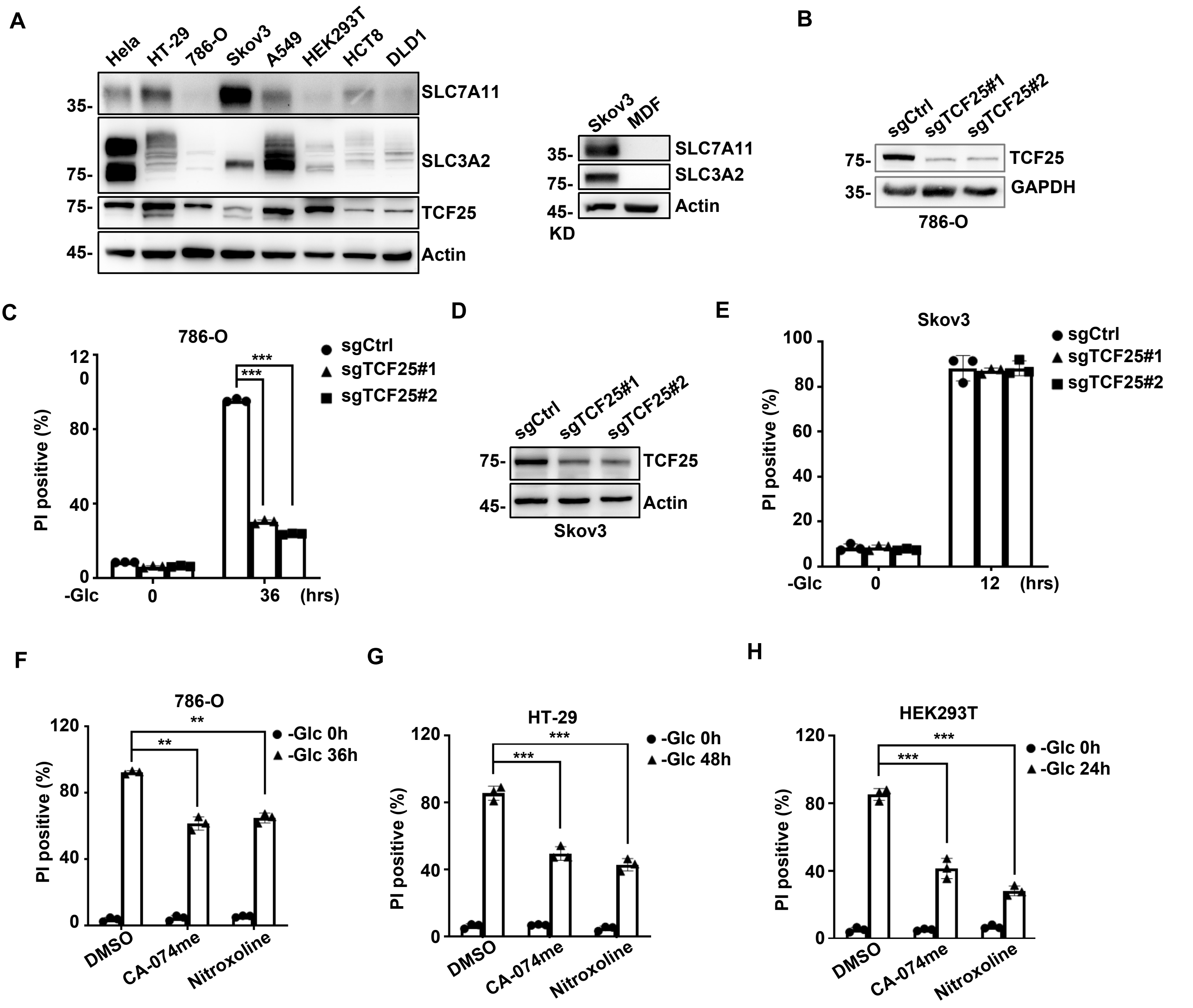

Fig. S8 TCF25 mediates glucose starvation-induced cell death in SLC7A11 low-expressed cells. (A) Various types of cell lines as indicated were lysed and immunoblotted with the indicated antibodies. (B) 786-O or (D) Skov3 cells were stably transfected with sgRNA-Control (sgCtrl) and two individual sgRNAs targeting TCF25 (sgTCF25#1 and sgTCF25#2), respectively. TCF25 expression was examined by immunoblotting with its specific antibody. (C) sgCtrl, sgTCF25#1 and sgTCF25#2 786-O or (E) Skov3 cells were starved without glucose for indicated time points and the cell death was determined by PI staining. (F) 786-O (G) HT-29 or (H) HEK293T cells were firstly starved without glucose for indicated time points and then supplied with CA-074me (1μ M) or Nitroxoline (5 μ M) for additional 12 hrs. Cell death was determined by PI staining. All western data are representative of three independent experiments. Bar graphs represent the mean ± SD from three independent experiments. Statistical analysis was performed using a two-sided student's t-test. The levels of significance were indicated by ＊＊＊ P <0.001. (Two-way ANOVA).

Figure S8

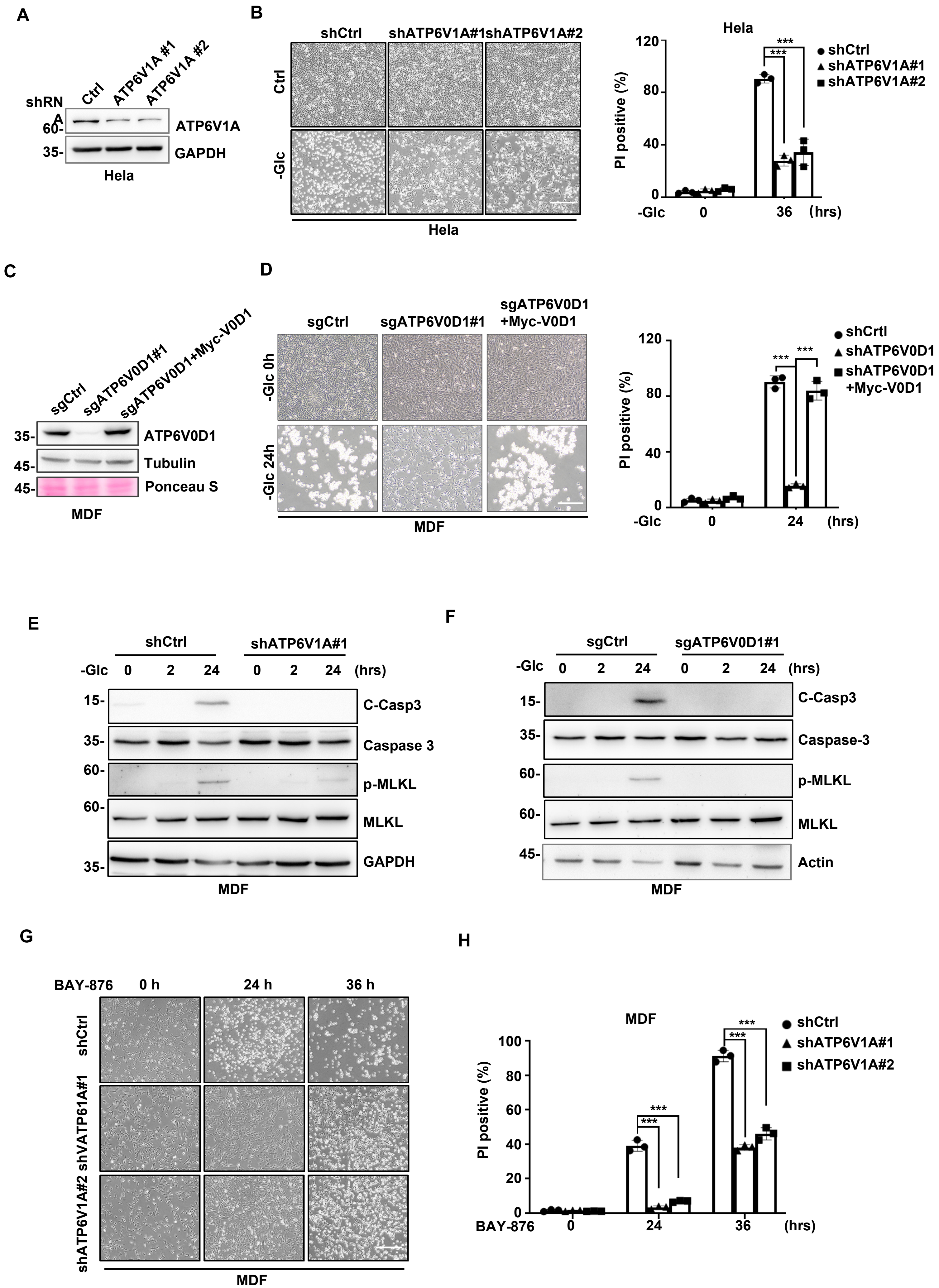
A
shRNA
Ctrl
ATP6V1A #1
ATP6V1A #2
60-
35-
ATP6V1A
GAPDH
Hela
B
shCtrl
shATP6V1A#1
shATP6V1A#2
Ctrl
-Glc
Hela
120
80
40
0
PI positive (%)
-Glc
0
36
(hrs)
***
***
shCtrl
shATP6V1A#1
shATP6V1A#2
C
sgCtrl
sgATP6V0D1#1
sgATP6V0D1+Myc-V0D1
35-
45-
45-
ATP6V0D1
Tubulin
Ponceau S
MDF
D
sgCtrl
sgATP6V0D1#1
sgATP6V0D1
+Myc-V0D1
-Glc 0h
-Glc 24h
MDF
120
80
40
0
PI positive (%)
-Glc
0
24
(hrs)
***
***
shCrtl
shATP6V0D1
shATP6V0D1
+Myc-V0D1
E
shCtrl
shATP6V1A#1
-Glc
0
2
24
0
2
24
(hrs)
15-
35-
60-
60-
35-
C-Casp3
Caspase 3
p-MLKL
MLKL
GAPDH
MDF
F
sgCtrl
sgATP6V0D1#1
-Glc
0
2
24
0
2
24
(hrs)
15-
35-
60-
60-
45-
C-Casp3
Caspase-3
p-MLKL
MLKL
Actin
MDF
G
BAY-876
0 h
24 h
36 h
shCtrl
shVATP61A#1
shATP6V1A#2
MDF
H
MDF
100
80
60
40
20
0
PI positive (%)
BAY-876
0
24
36
(hrs)
***
***
***
***
shCtrl
shATP6V1A#1
shATP6V1A#2

Figure S9

I

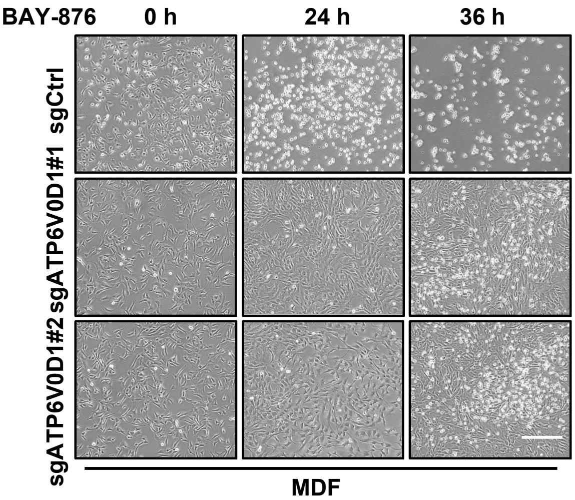

J

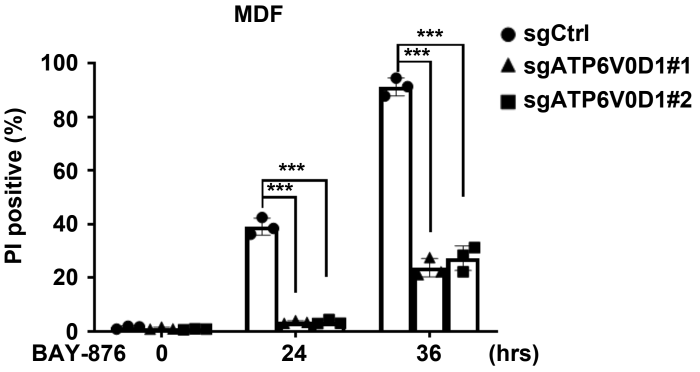

K

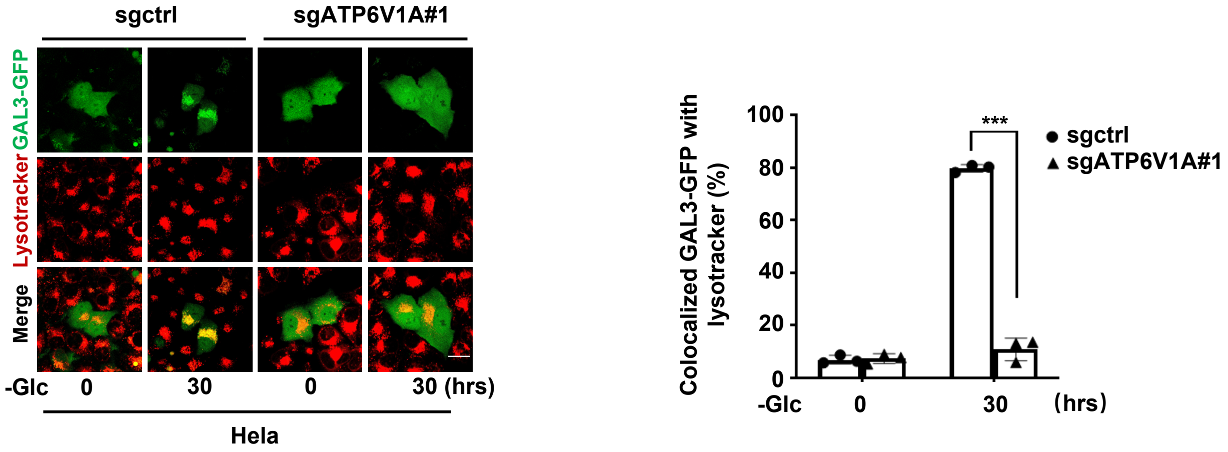

Figure S9

Fig. S9 TCF25-V-ATPase signaling axis is essential for glucose starvation-induced cell death

(A) Hela cells were stably transfected with shRNA-Control (shCtrl) and two individual shRNAs targeting ATP6V1A (shATP6V1A#1 and shATP6V1A#2), respectively. ATP6V1A expression was examined by immunoblotting with its specific antibody. (B) shCtrl, shATP6V1A#1 and shATP6V1A#2 Hela cells were starved without glucose for 36 hrs and the representative images were shown (left). Scale bar, 100 µm. Cell death of the Hela cells was determined by PI staining (right). (C) ATP6V0D1 expression was examined in sgCtrl, sgATP6V0D1#1 and ATP6V0D1 reconstituted MDFs by immunoblotting with its specific antibody. Ponceau S staining was used as a loading control. (D) sgCtrl, sgATP6V0D1#1 and ATP6V0D1 reconstituted MDFs were starved without glucose for 24 hrs and the representative images were shown (left). Scale bar, 100 µm. Cell death of the MDF cells was determined by PI staining (right). (E) shCtrl and shATP6V1A#1 MDFs were starved without glucose at the indicated time points. Cells were lysed and immunoblotted with the indicated antibodies. (F) sgCtrl and sgATP6V0D1#1 MDFs were starved without glucose at the indicated time points. Cells were lysed and immunoblotted with the indicated antibodies. (G) shCtrl, shATP6V1A#1 and shATP6V1A#2 MDFs were treated with BAY-876 for indicated time points and the representative images were shown. Scale bar, 100 µm. (H) Cell death of the MDFs in (G) was determined by PI staining. (I) sgCtrl, sgATP6V0D1#1 and sgATP6V0D1#2 MDFs were treated with BAY-876 for indicated time points and the representative images were shown. Scale bar, 100 µm. (J) Cell death of the MDFs in (I) was determined by PI staining. (K) shCtrl and shATP6V1A Hela cells were transfected with GFP-Gal3 and then starved without glucose at the indicated time points. The cells were stained with LysoTracker™ Red. Left, representative confocal images of the cells were shown. Right, statistical analysis of the co-localized GFP-Gal3 with LysoTracker-Red. Scale bar, 20 µm. All western data are representative of three independent experiments. Bar graphs represent the mean ± SD from three independent experiments. Statistical analysis was performed using a two-sided student's t-test. The levels of significance were indicated by＊＊＊ $P < 0.001$. (Two-way ANOVA).

Data S1: Raw data of Western Blot

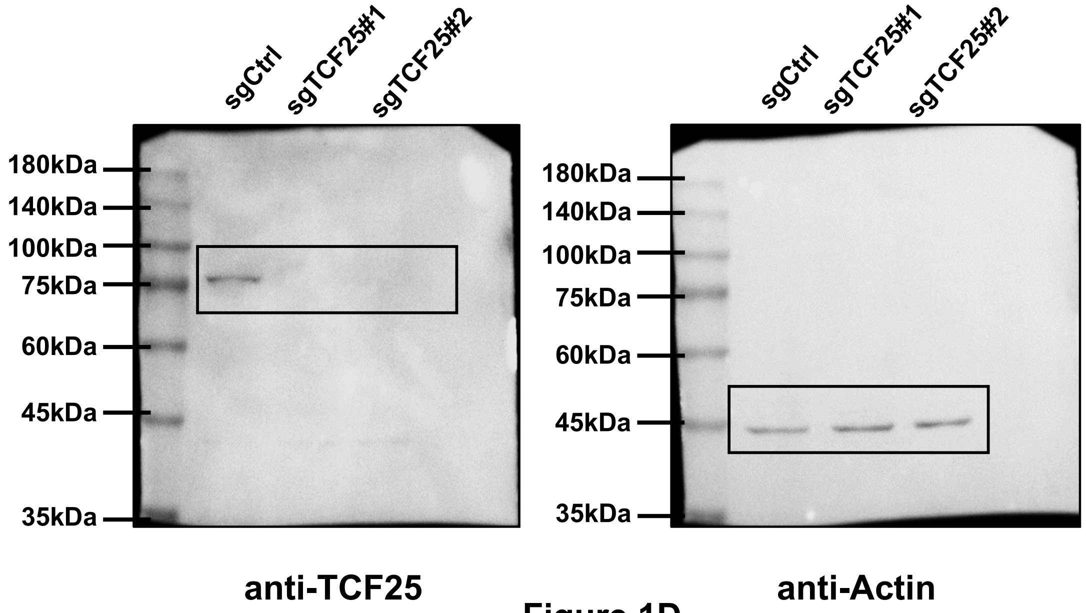

Figure 1D

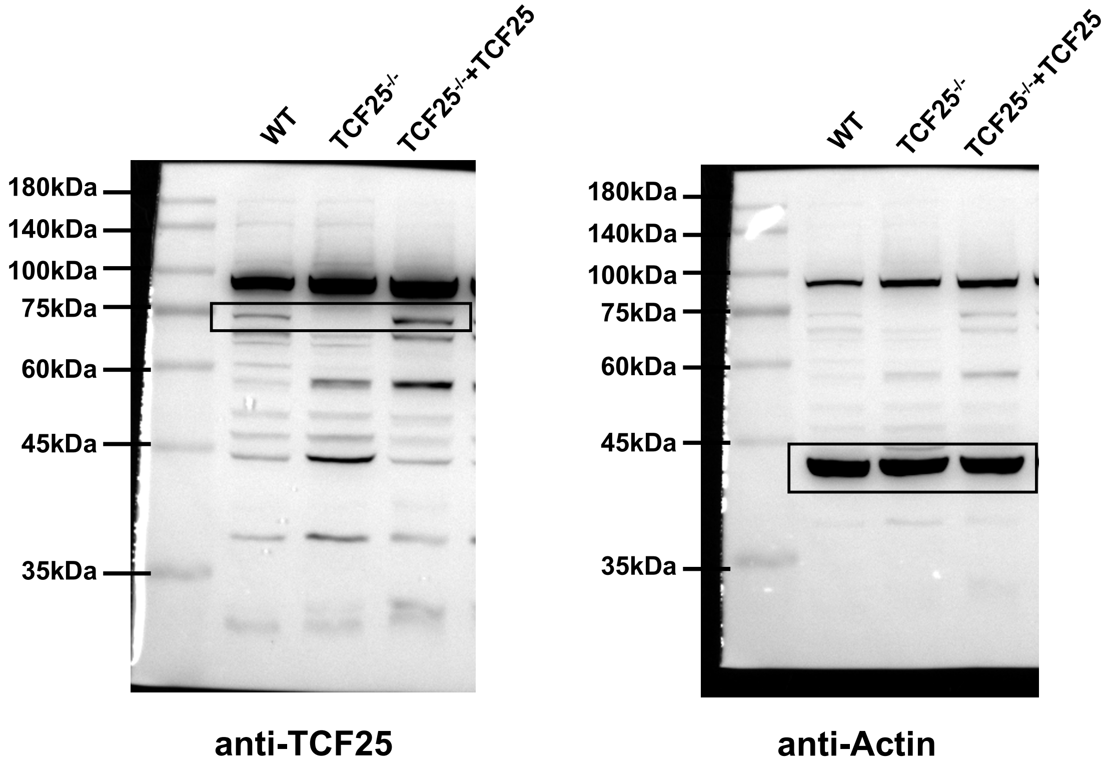

Figure 1K

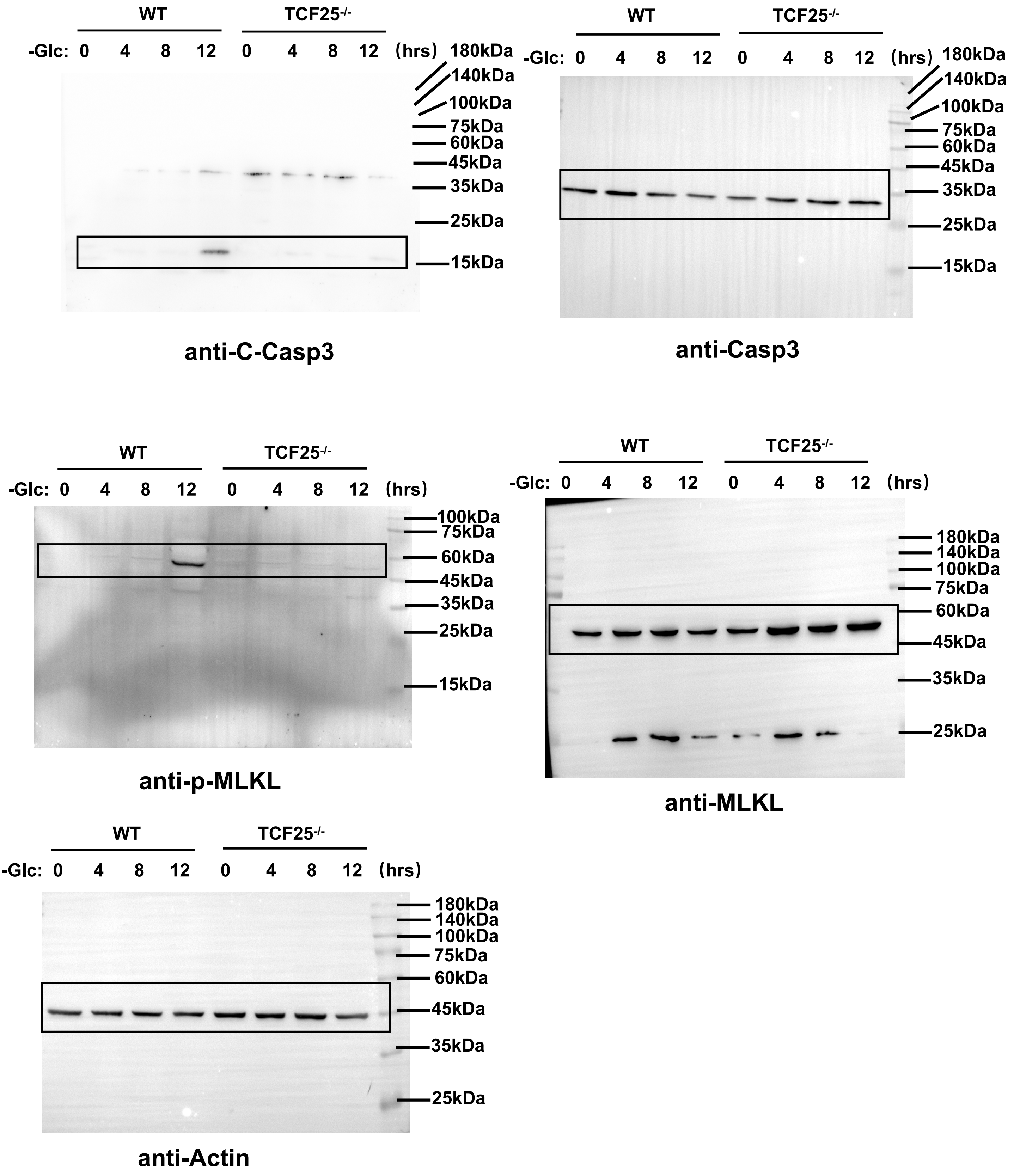
WT
TCF25-/-
-Glc: 0 4 8 12 0 4 8 12 (hrs)
180kDa
140kDa
100kDa
75kDa
60kDa
45kDa
35kDa
25kDa
15kDa
anti-C-Casp3
anti-Casp3
anti-p-MLKL
anti-MLKL
anti-Actin

Figure 1M

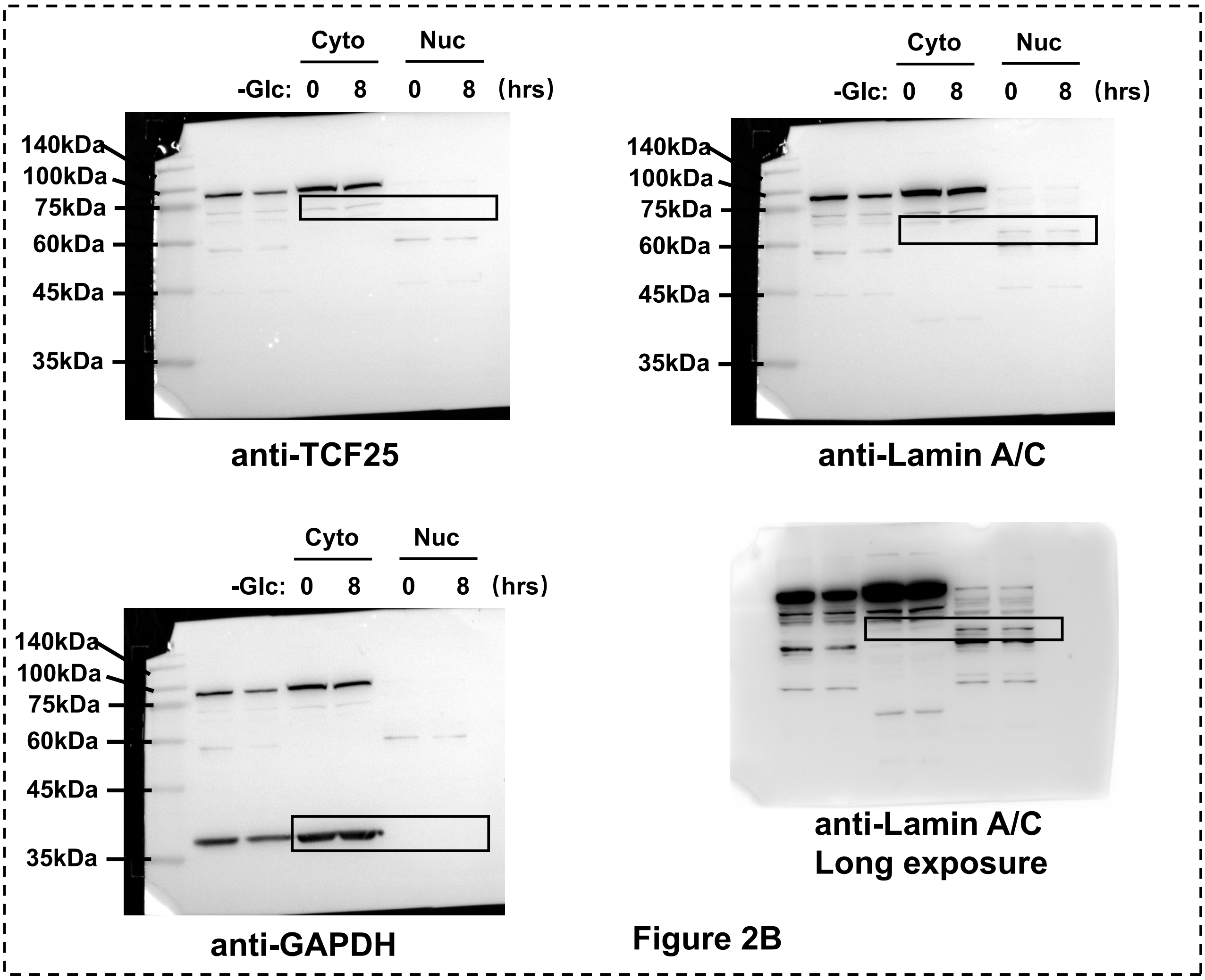

Figure 2B

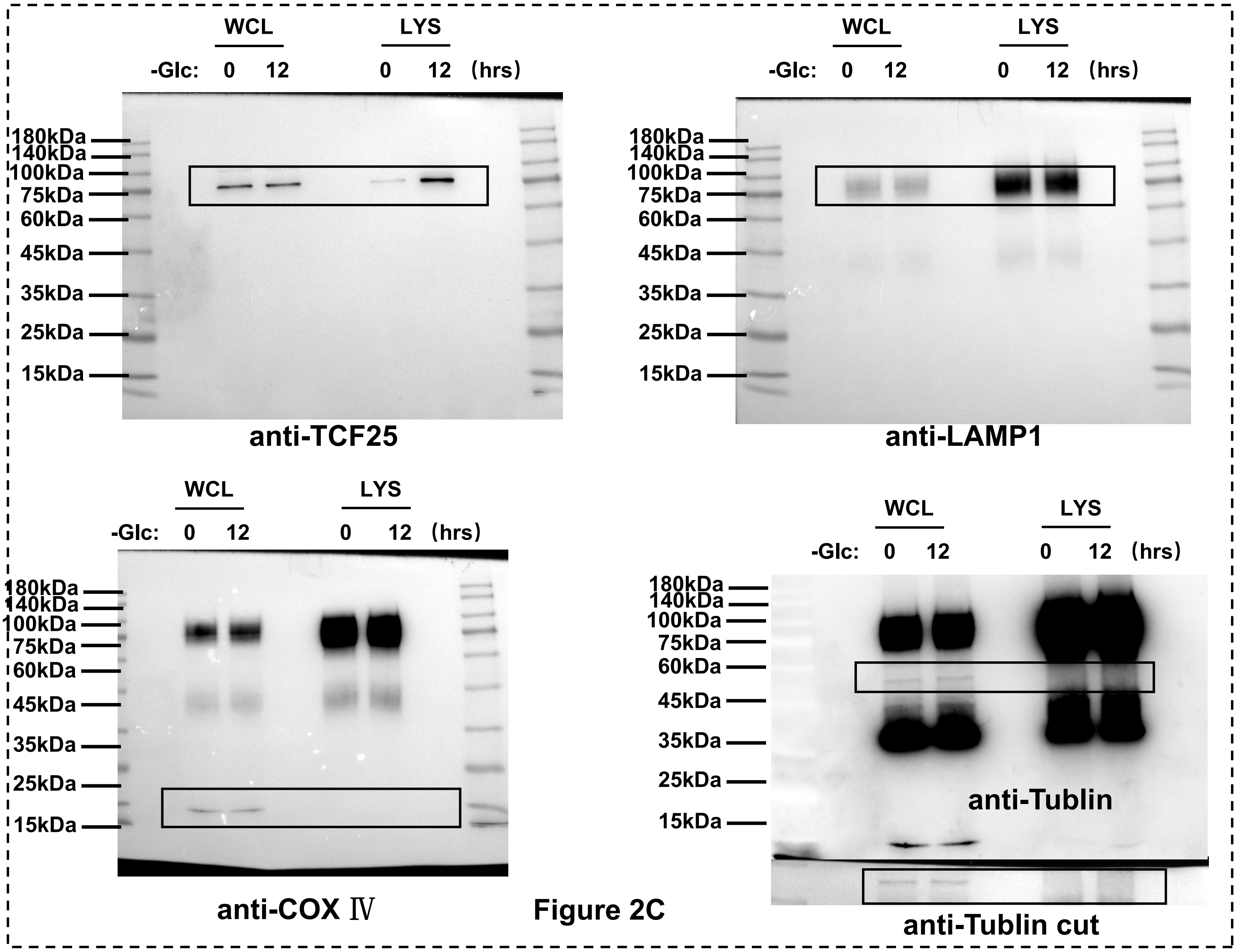

Figure 2C

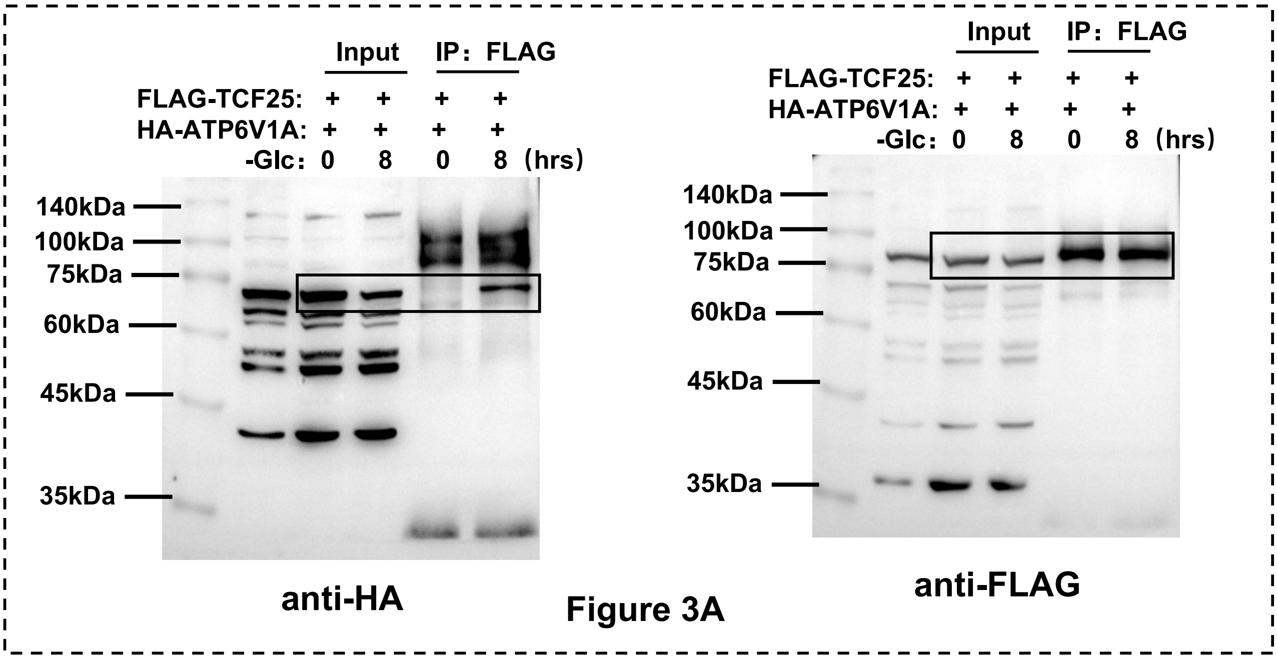

Figure 3A

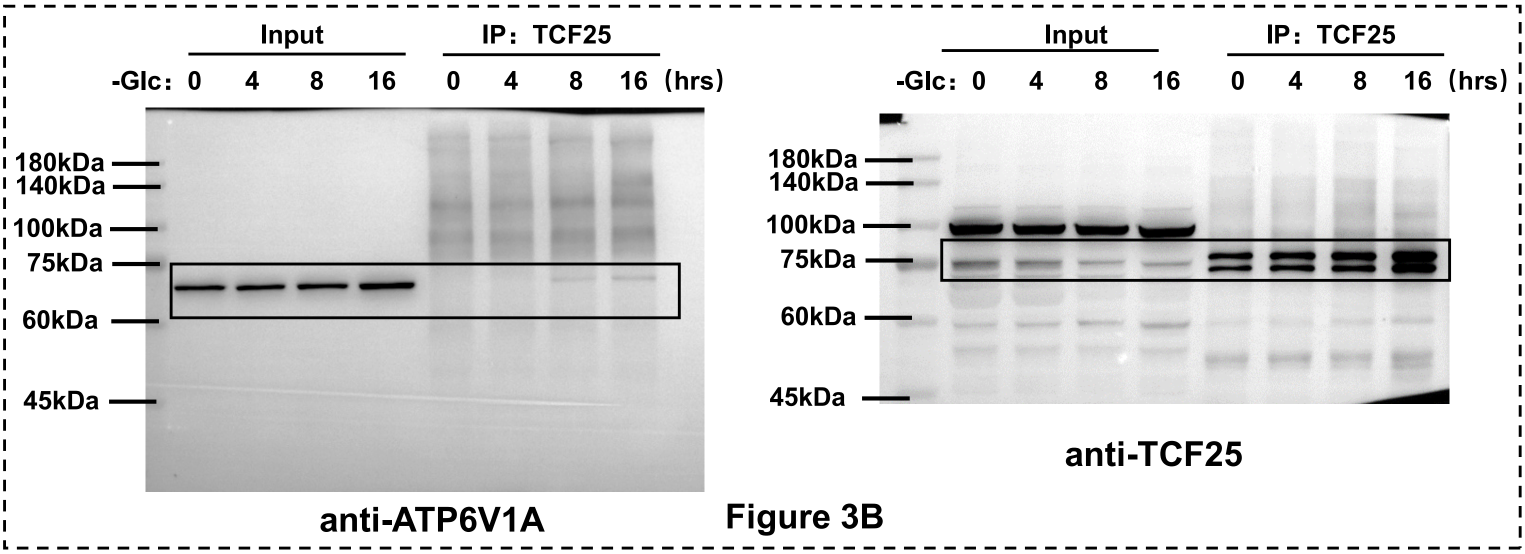

Figure 3B

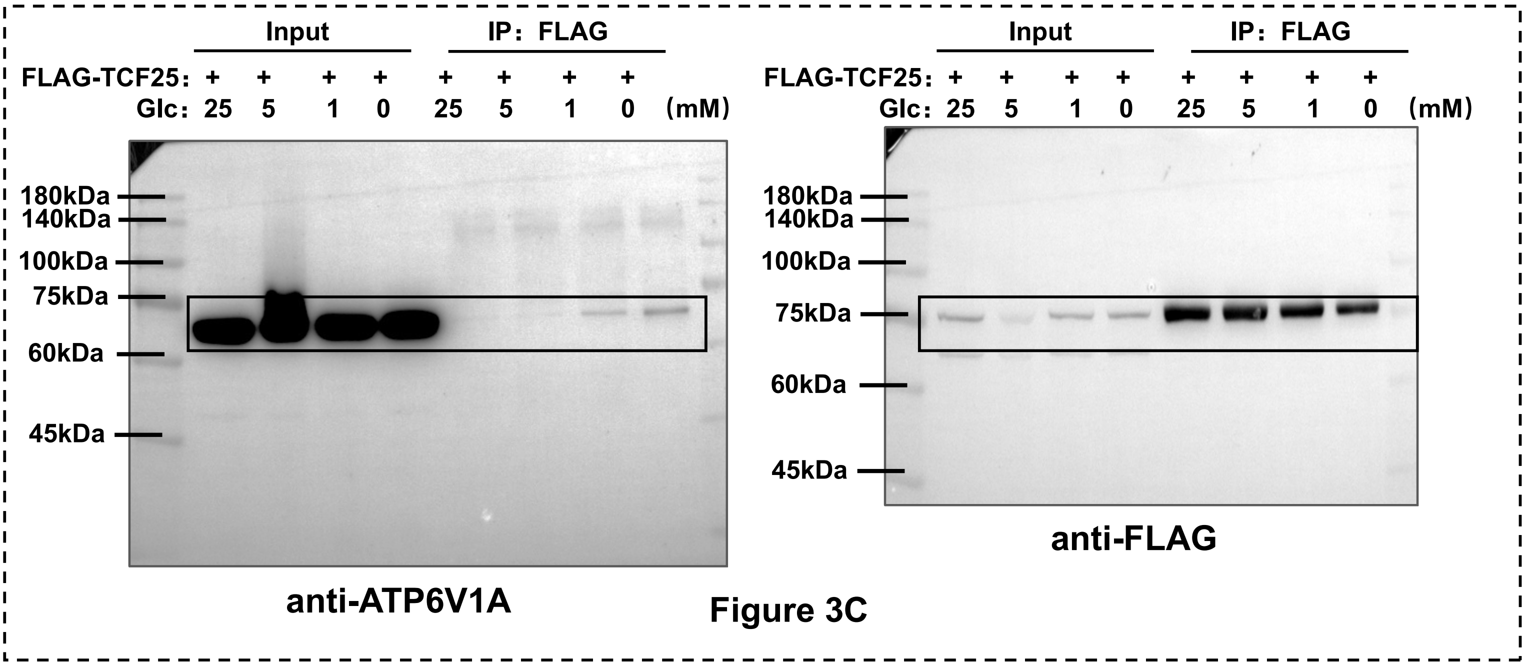

Figure 3C

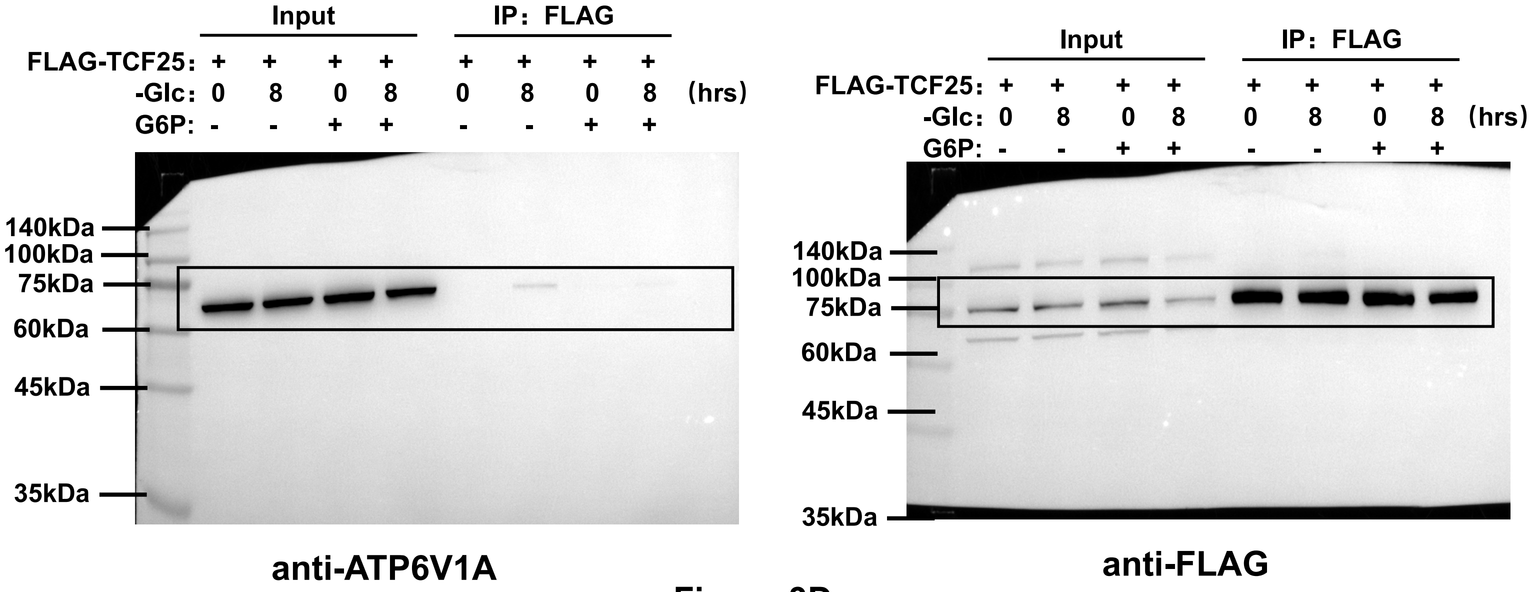

Figure 3D

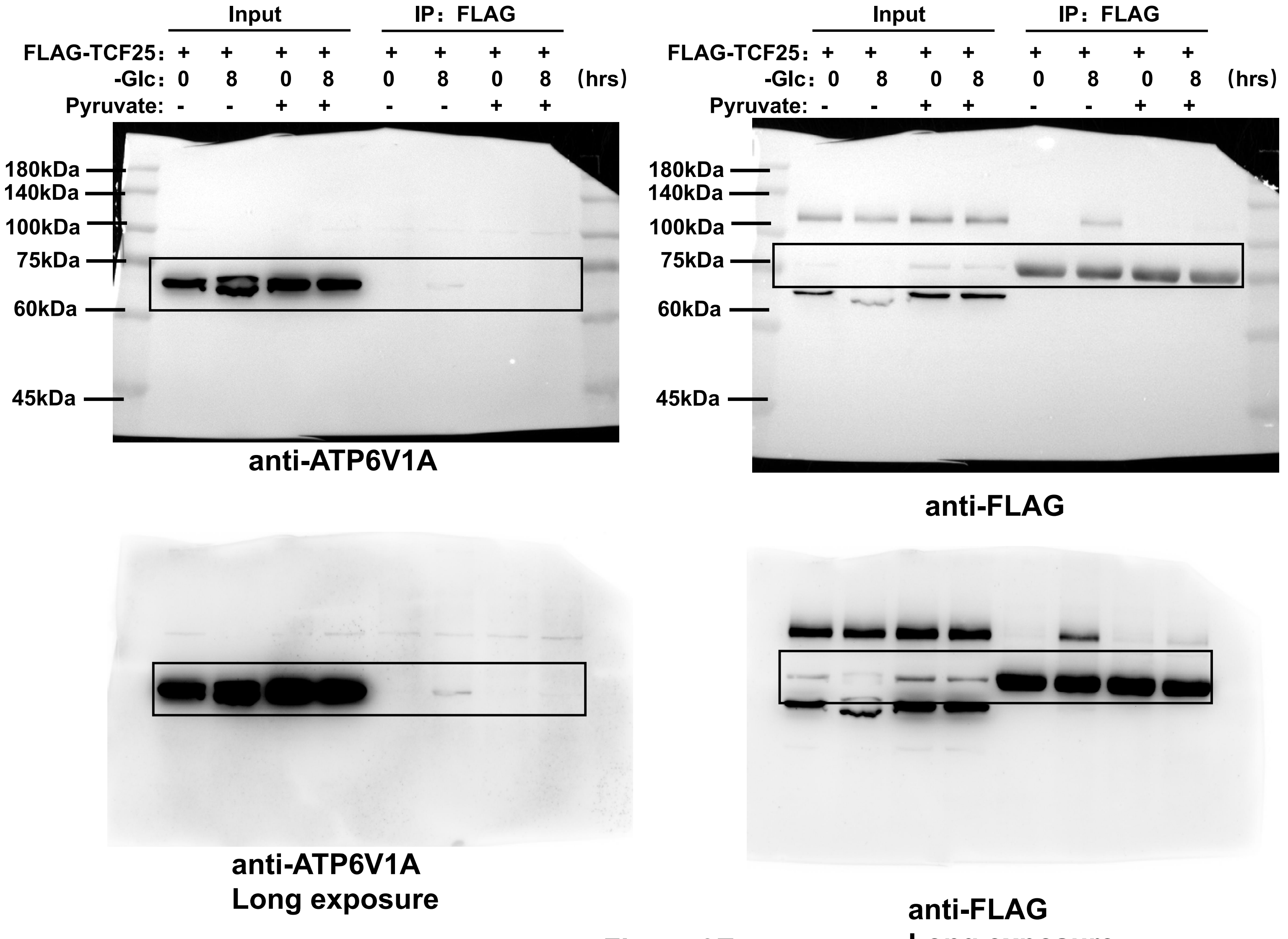

Figure 3E

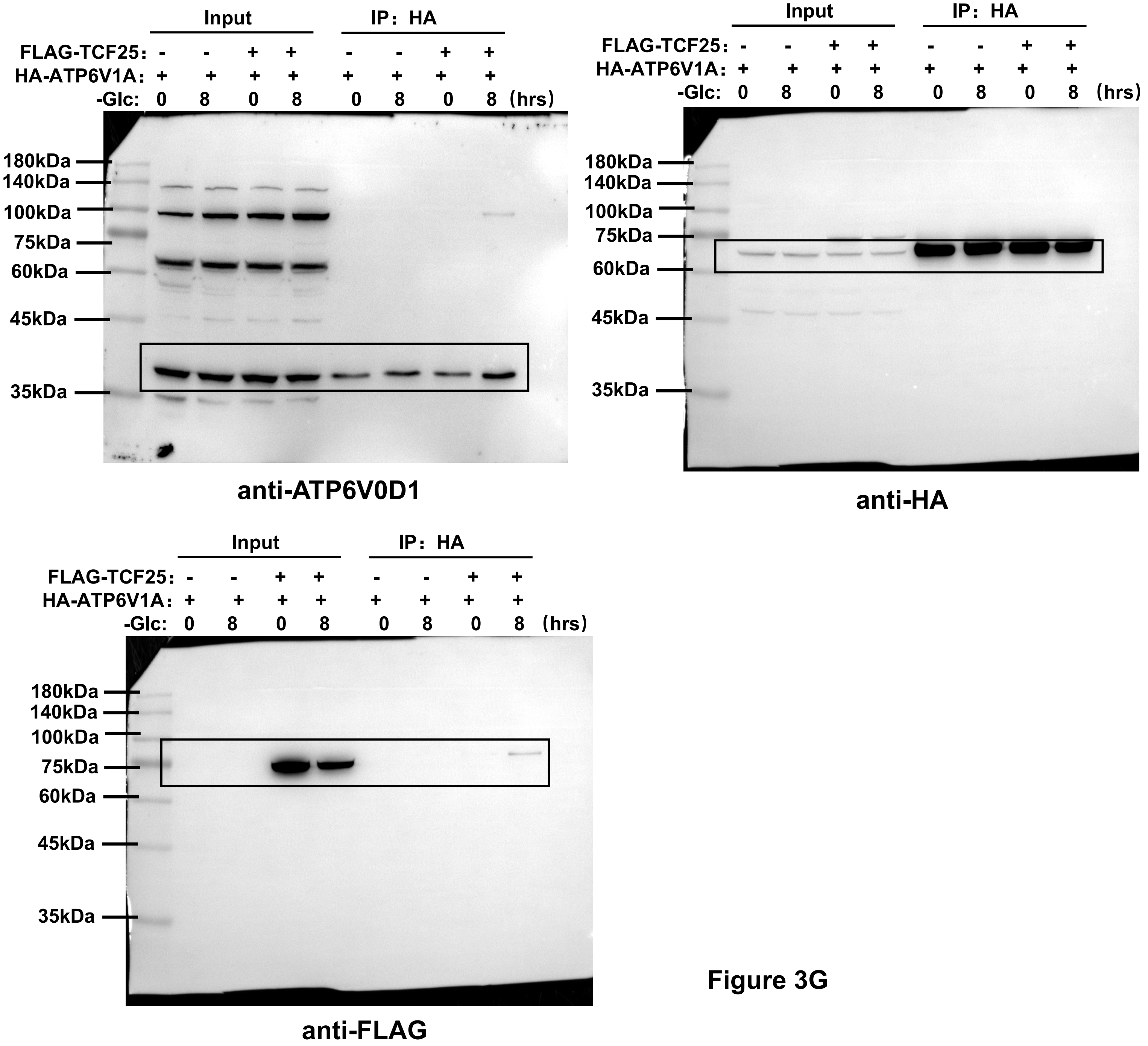

Input
IP：HA
FLAG-TCF25：
HA-ATP6V1A：
-Glc:
(hrs)
180kDa
140kDa
100kDa
75kDa
60kDa
45kDa
35kDa
anti-ATP6V0D1
anti-HA
anti-FLAG

Figure 3G

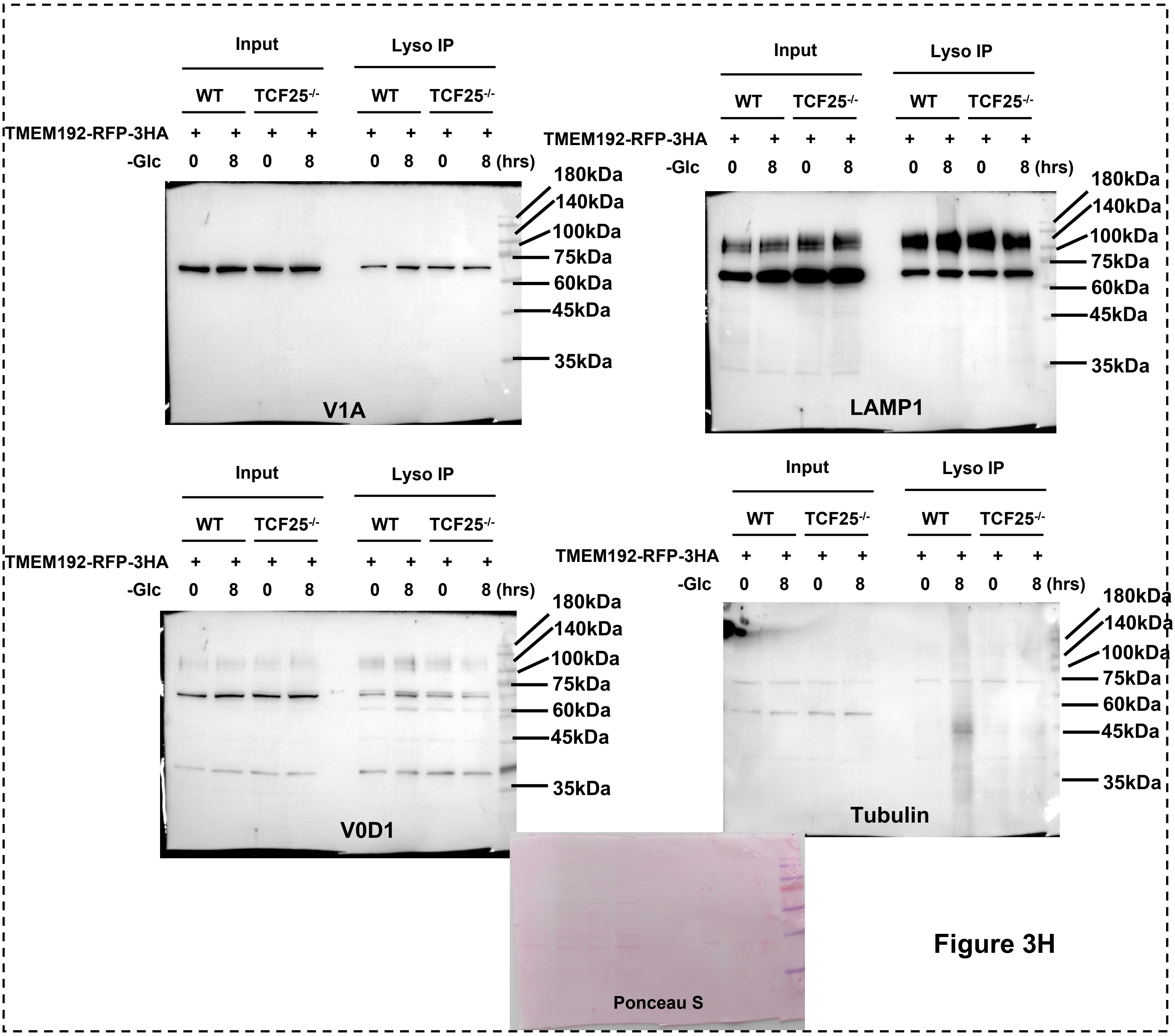

Figure 3H

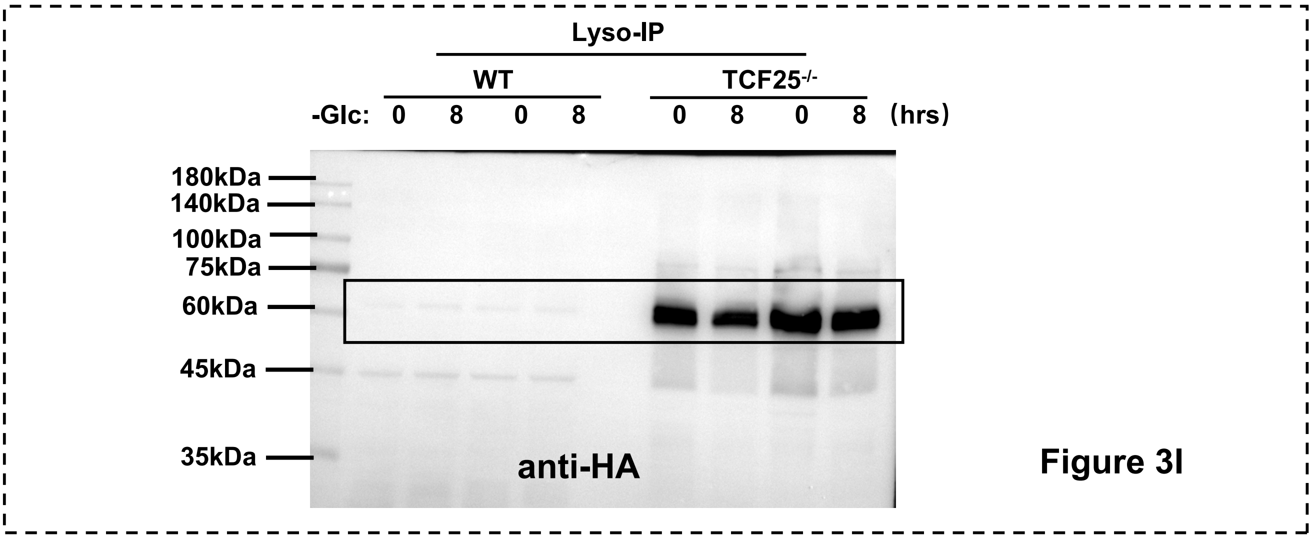

Figure 3I

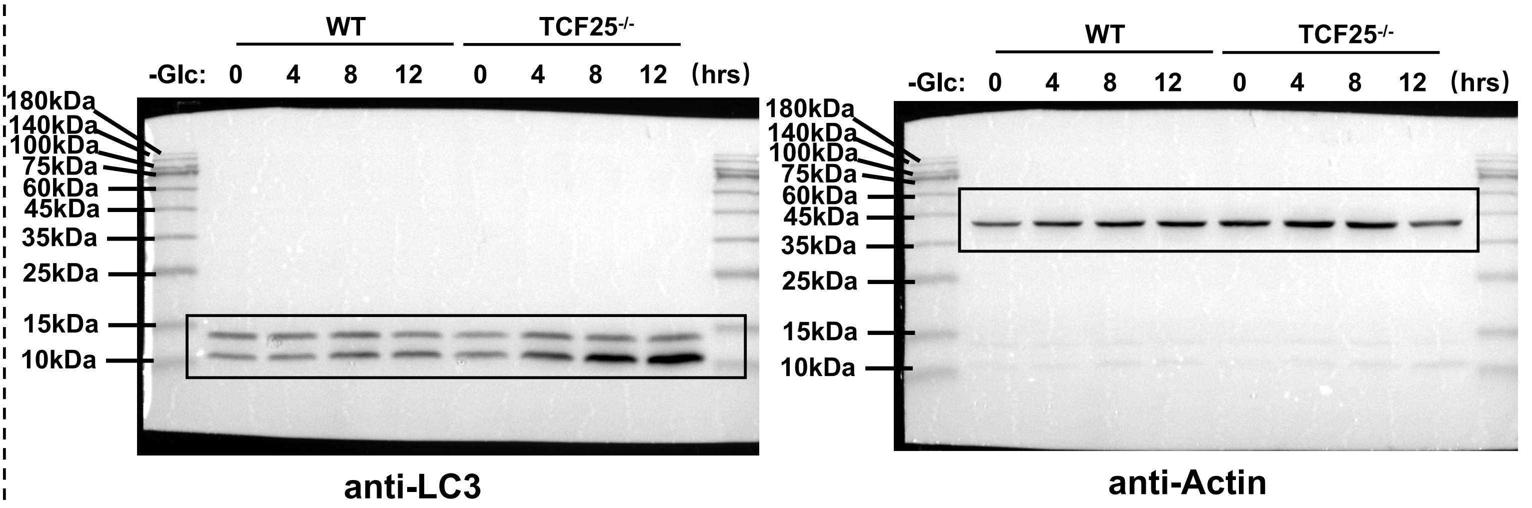

Figure 5A

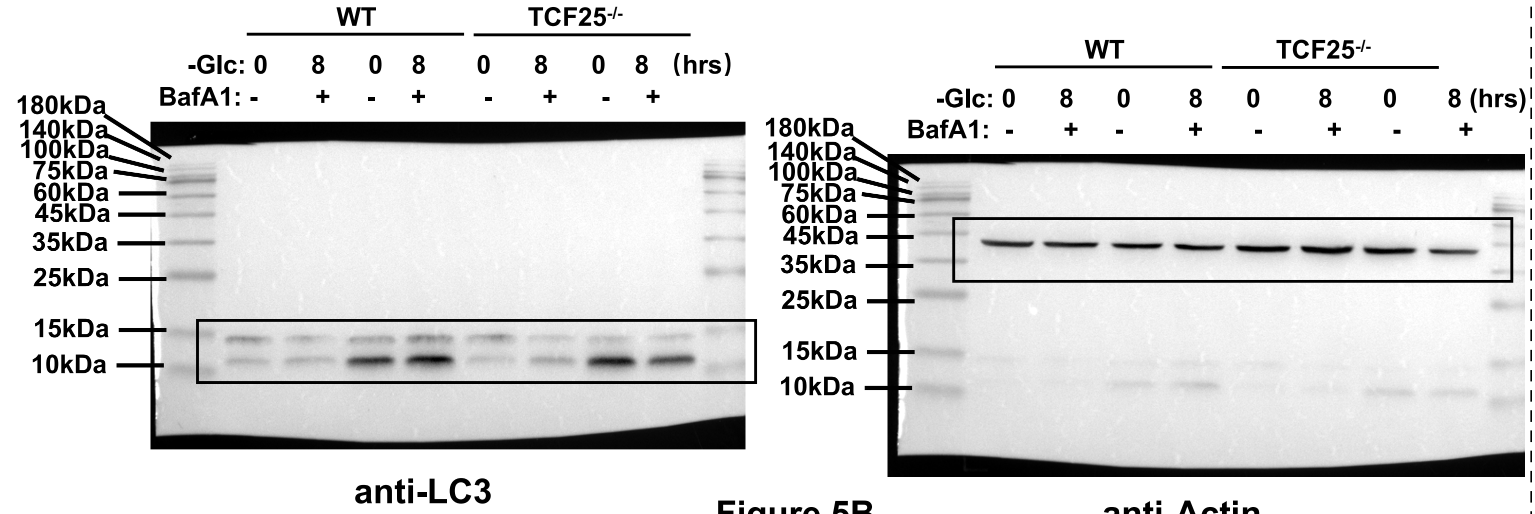

Figure 5B

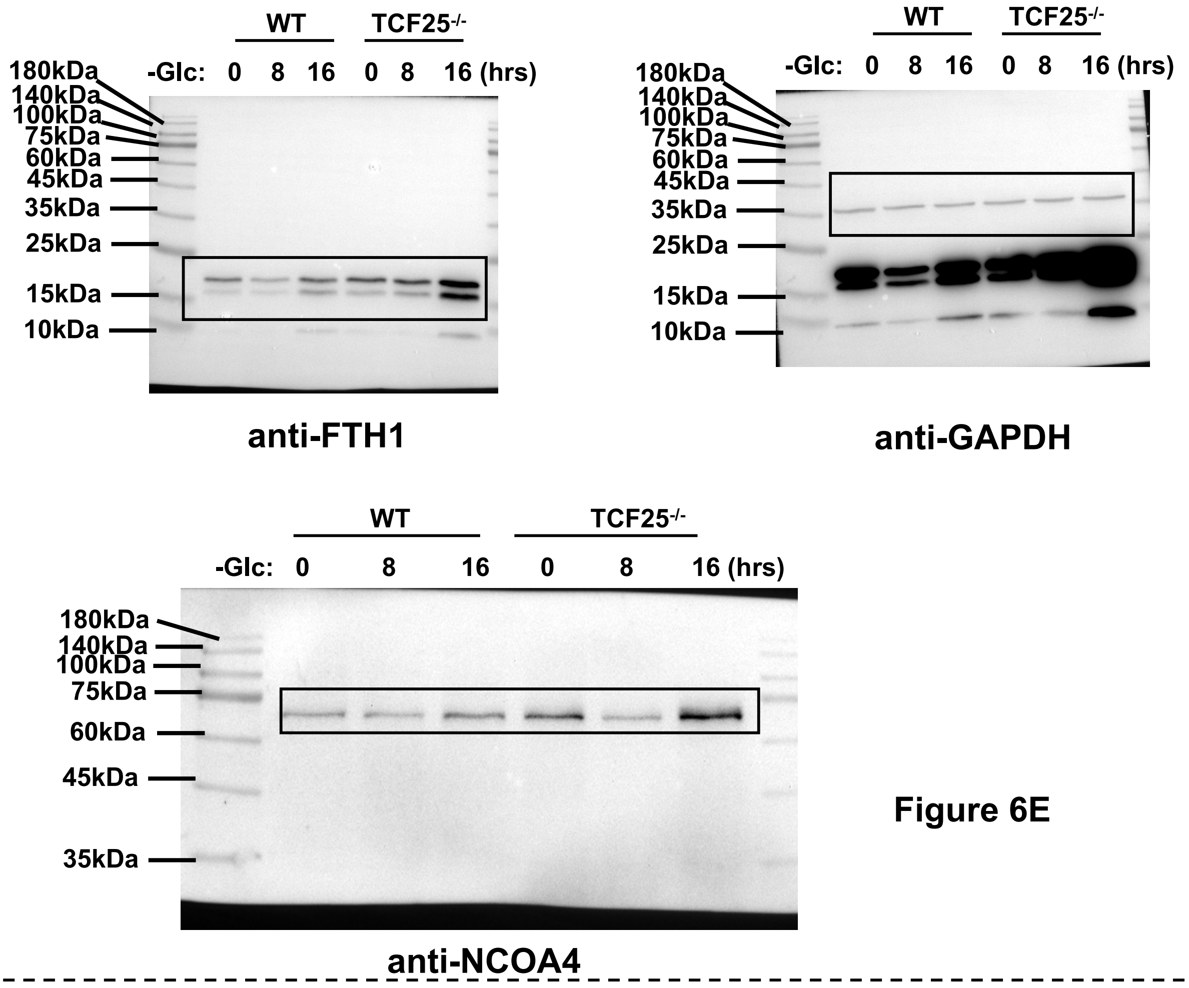

Figure 6E

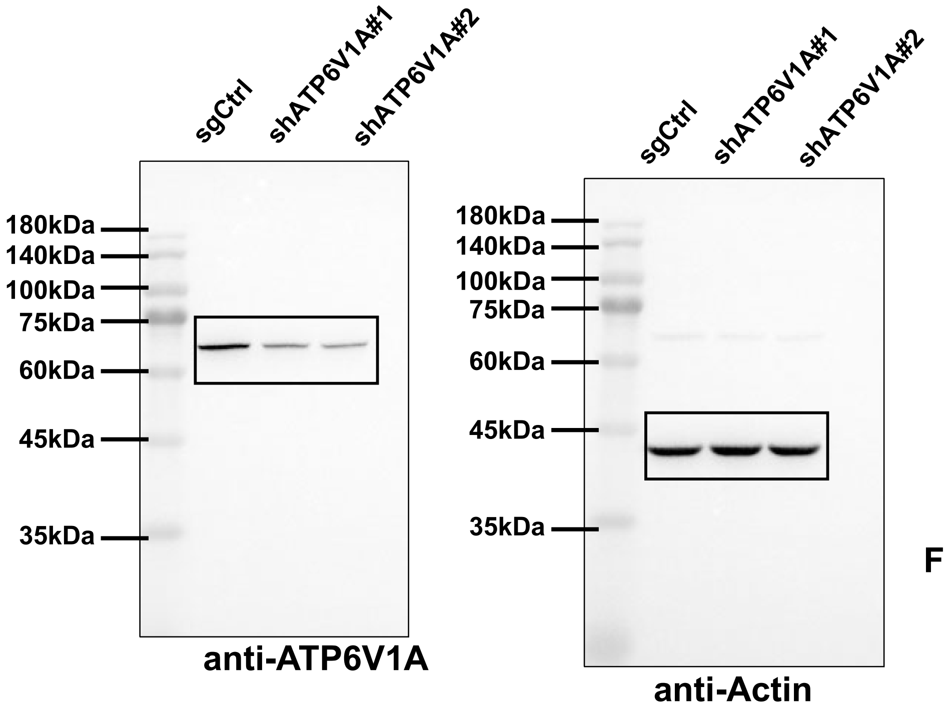

Figure 7A

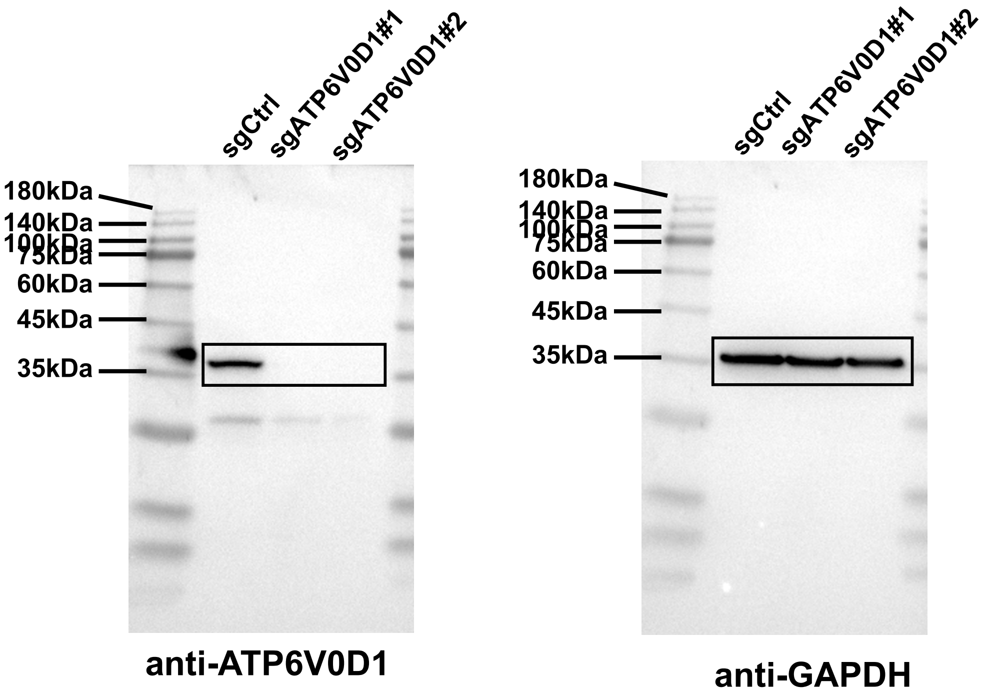

Figure 7D

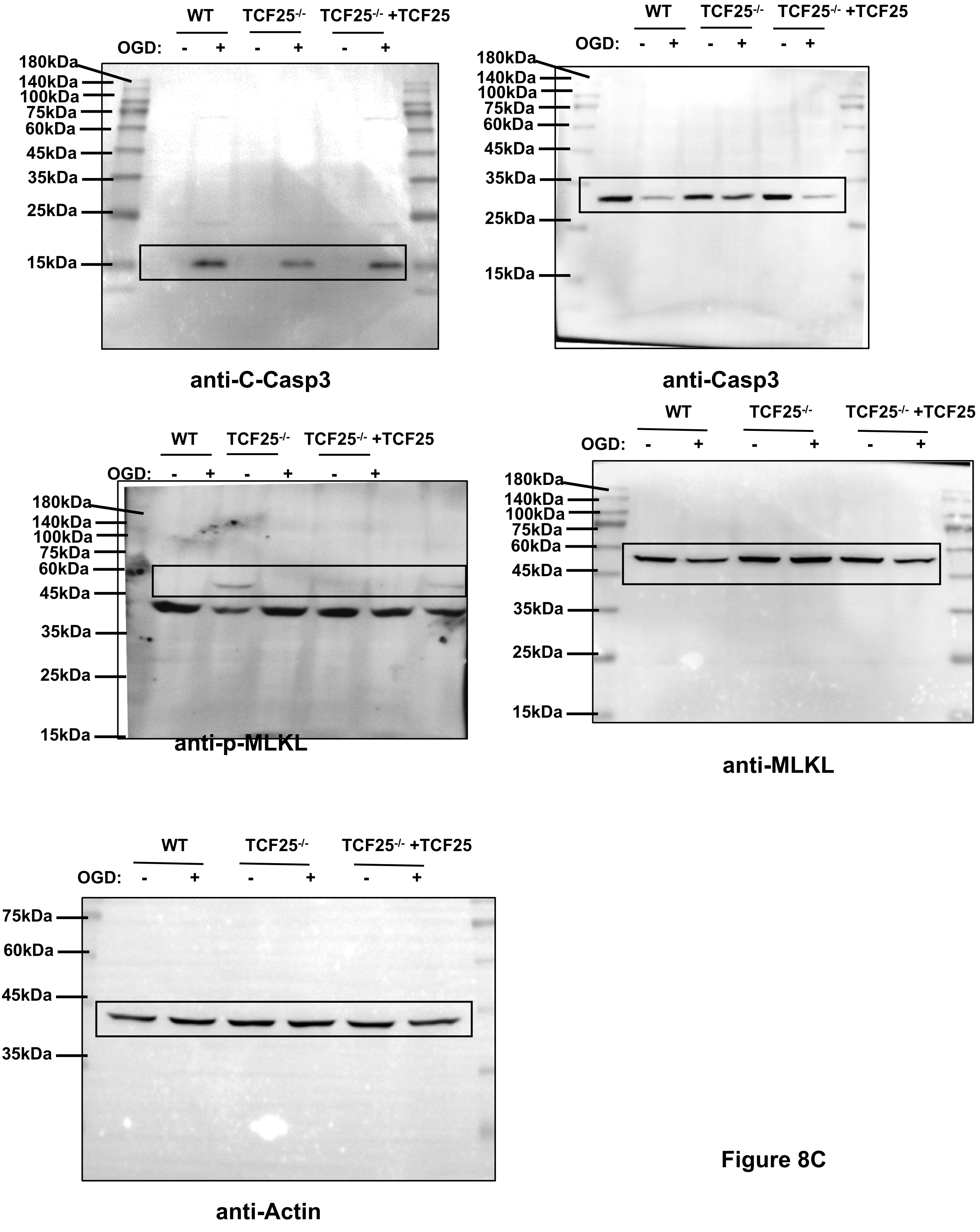
WT
TCF25-/-
TCF25-/- +TCF25
OGD: - + - + - +
180kDa
140kDa
100kDa
75kDa
60kDa
45kDa
35kDa
25kDa
15kDa
anti-C-Casp3
anti-Casp3
anti-p-MLKL
anti-MLKL
anti-Actin

Figure 8C

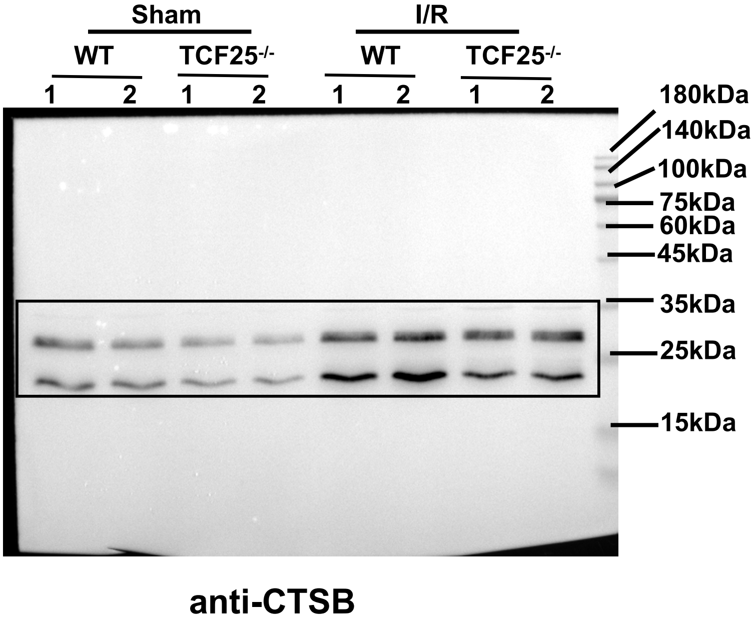
Sham
I/R
WT
TCF25-/-
1
2
180kDa
140kDa
100kDa
75kDa
60kDa
45kDa
35kDa
25kDa
15kDa
anti-CTSB

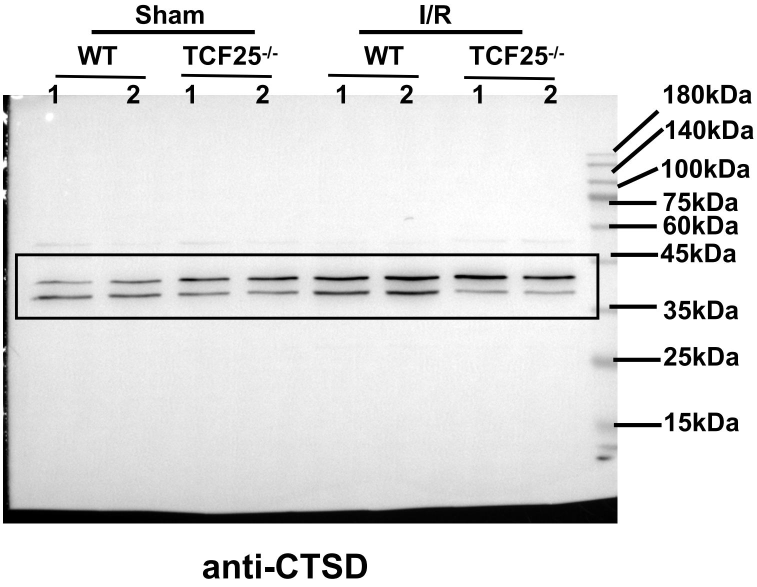
Sham
I/R
WT
TCF25-/-
1
2
180kDa
140kDa
100kDa
75kDa
60kDa
45kDa
35kDa
25kDa
15kDa
anti-CTSD

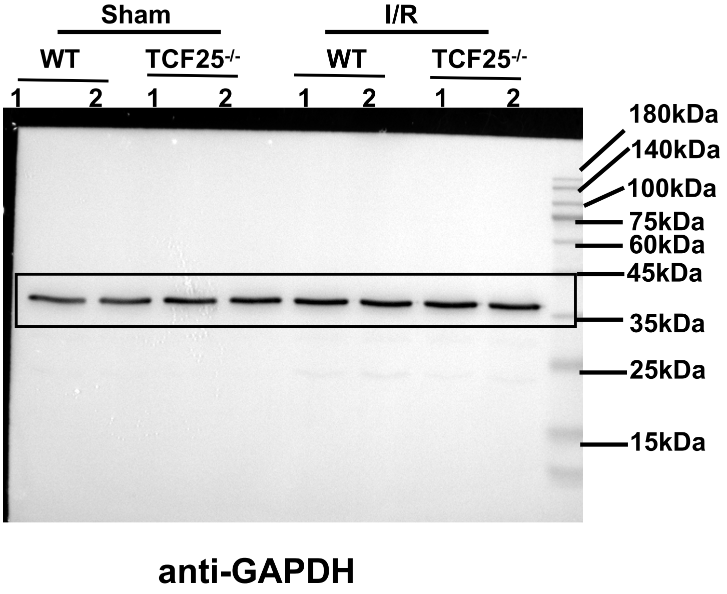
Sham
I/R
WT
TCF25-/-
1
2
180kDa
140kDa
100kDa
75kDa
60kDa
45kDa
35kDa
25kDa
15kDa
anti-GAPDH

Figure 8I

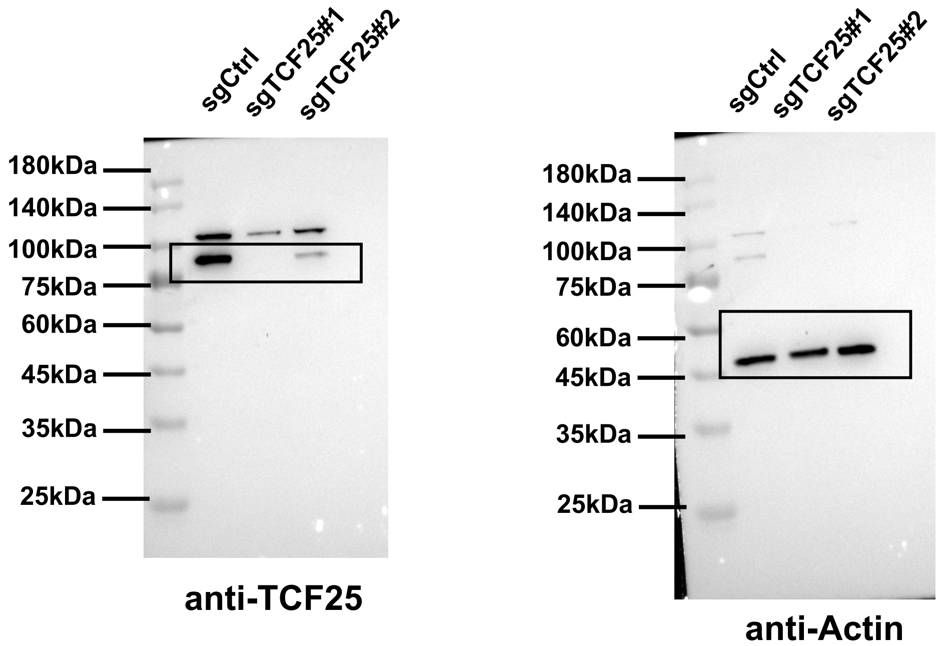

Figure S1A

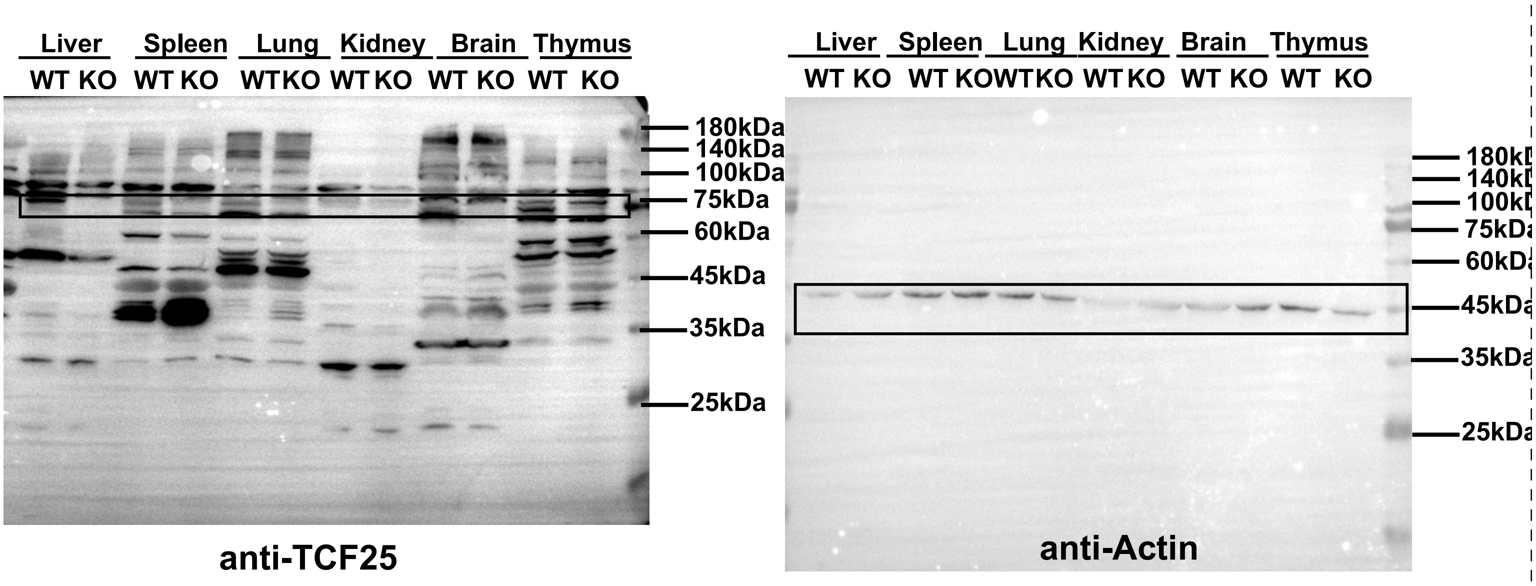

Figure S2C

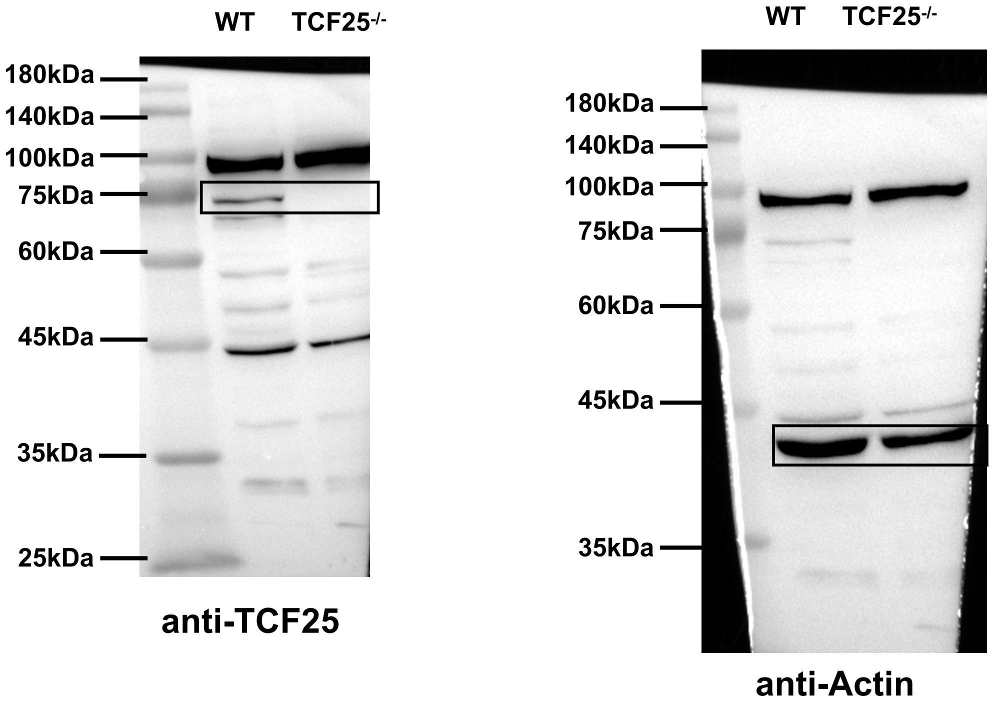

Figure S2F

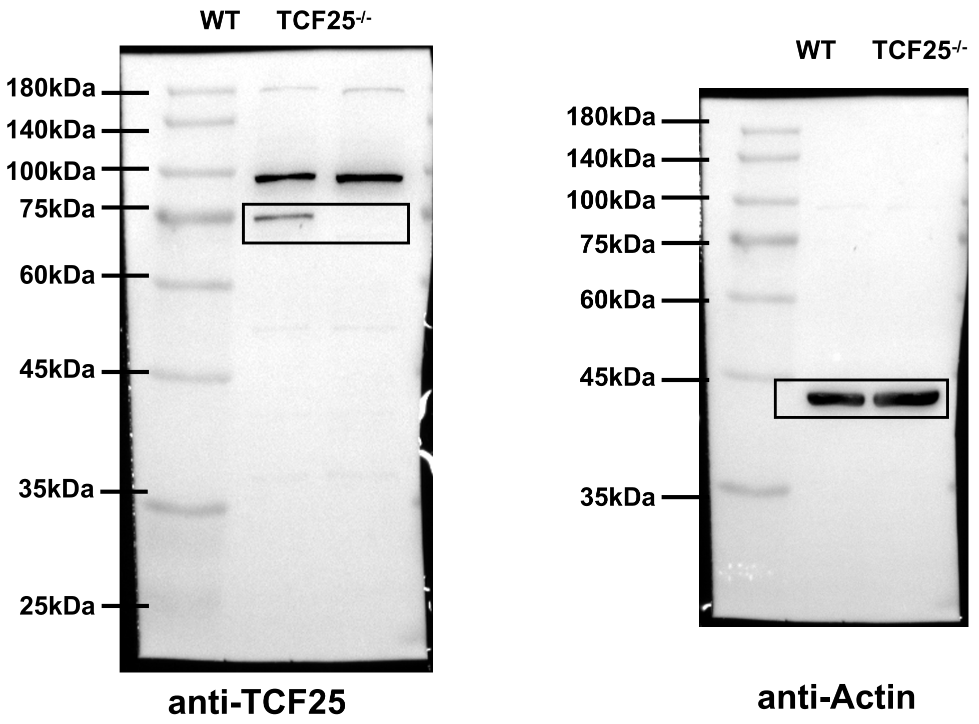

Figure S2I

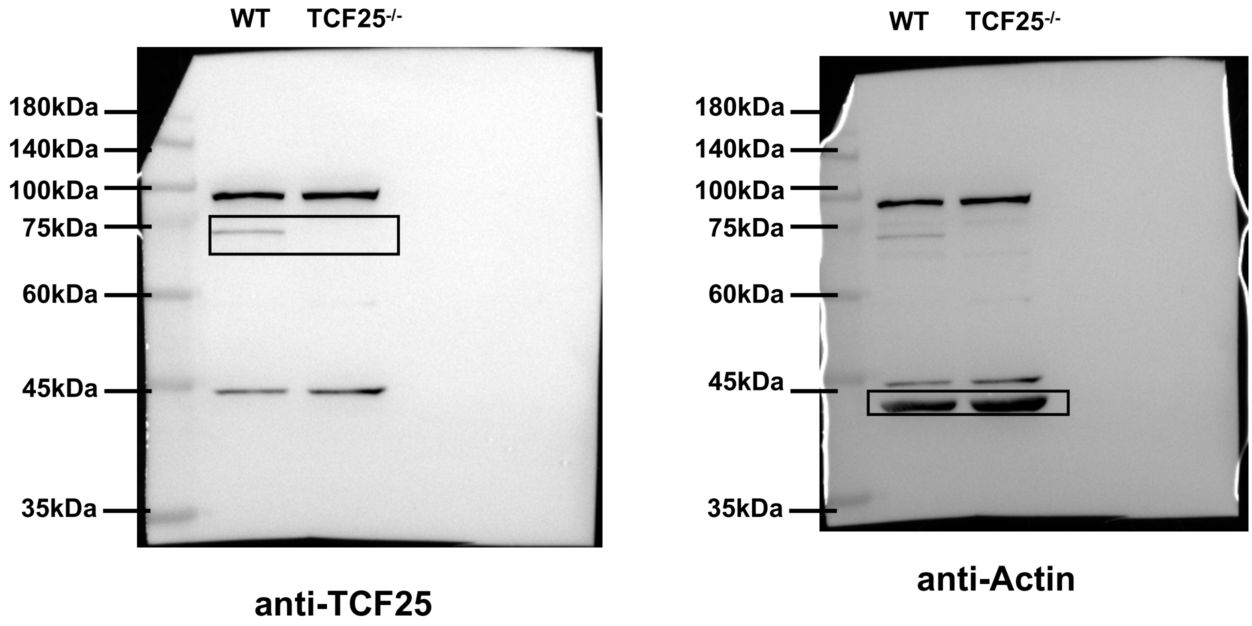

Figure S2L

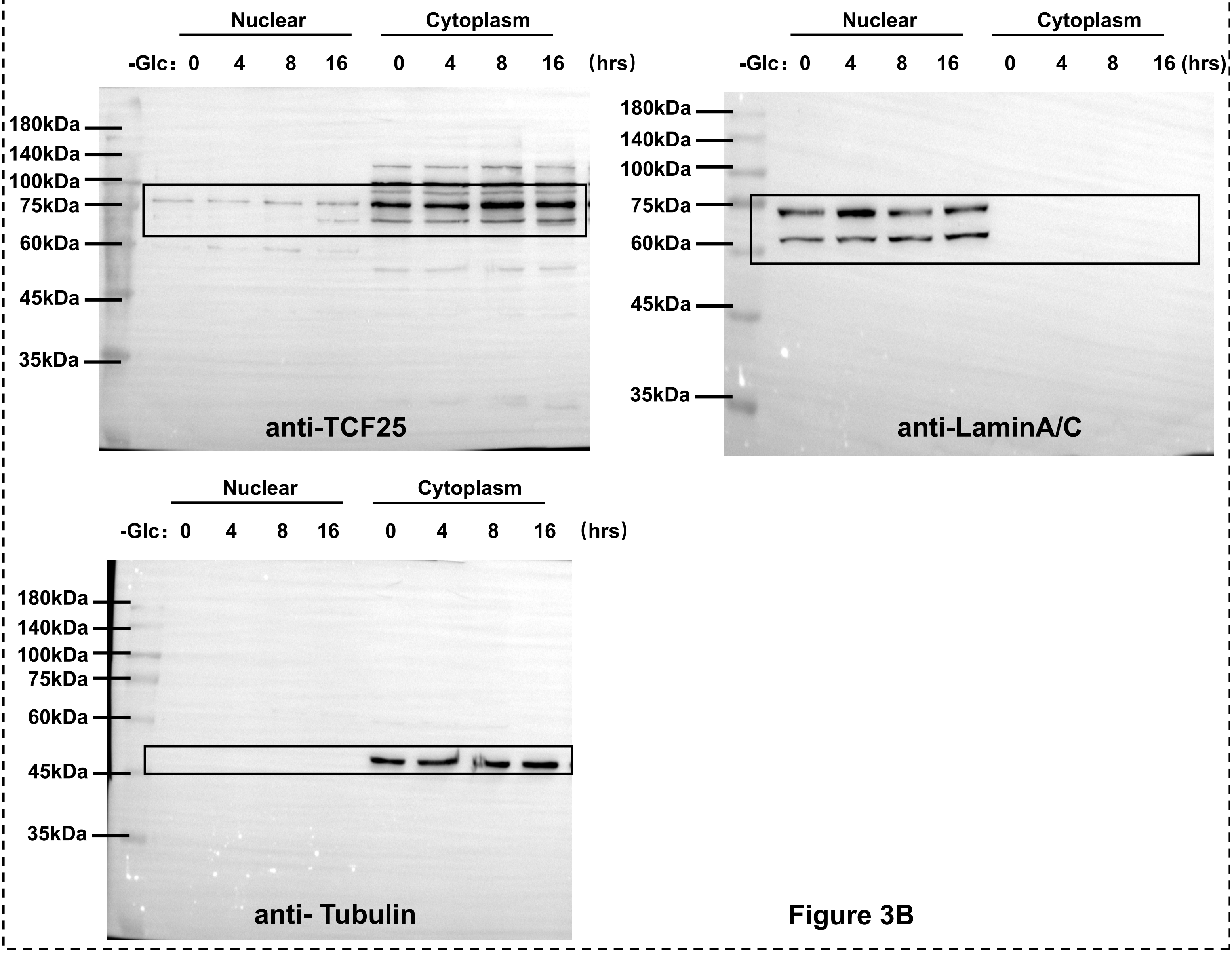

Figure 3B

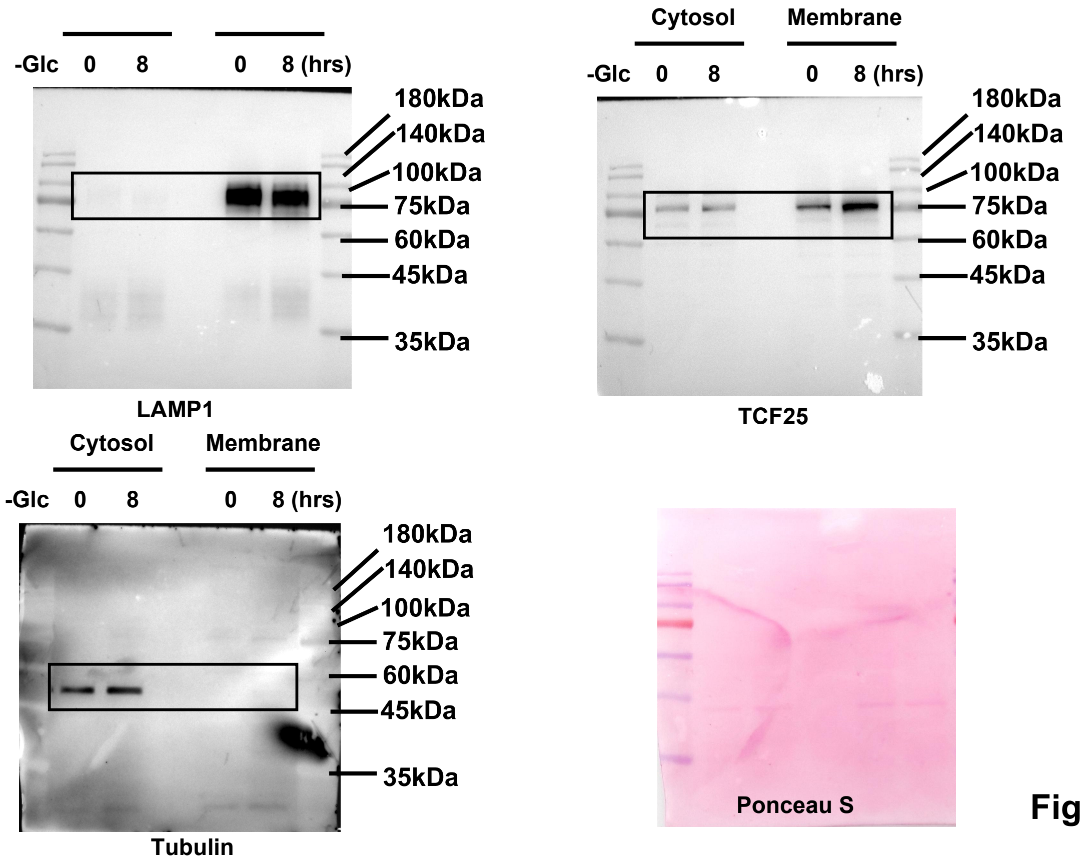

Figure S3E

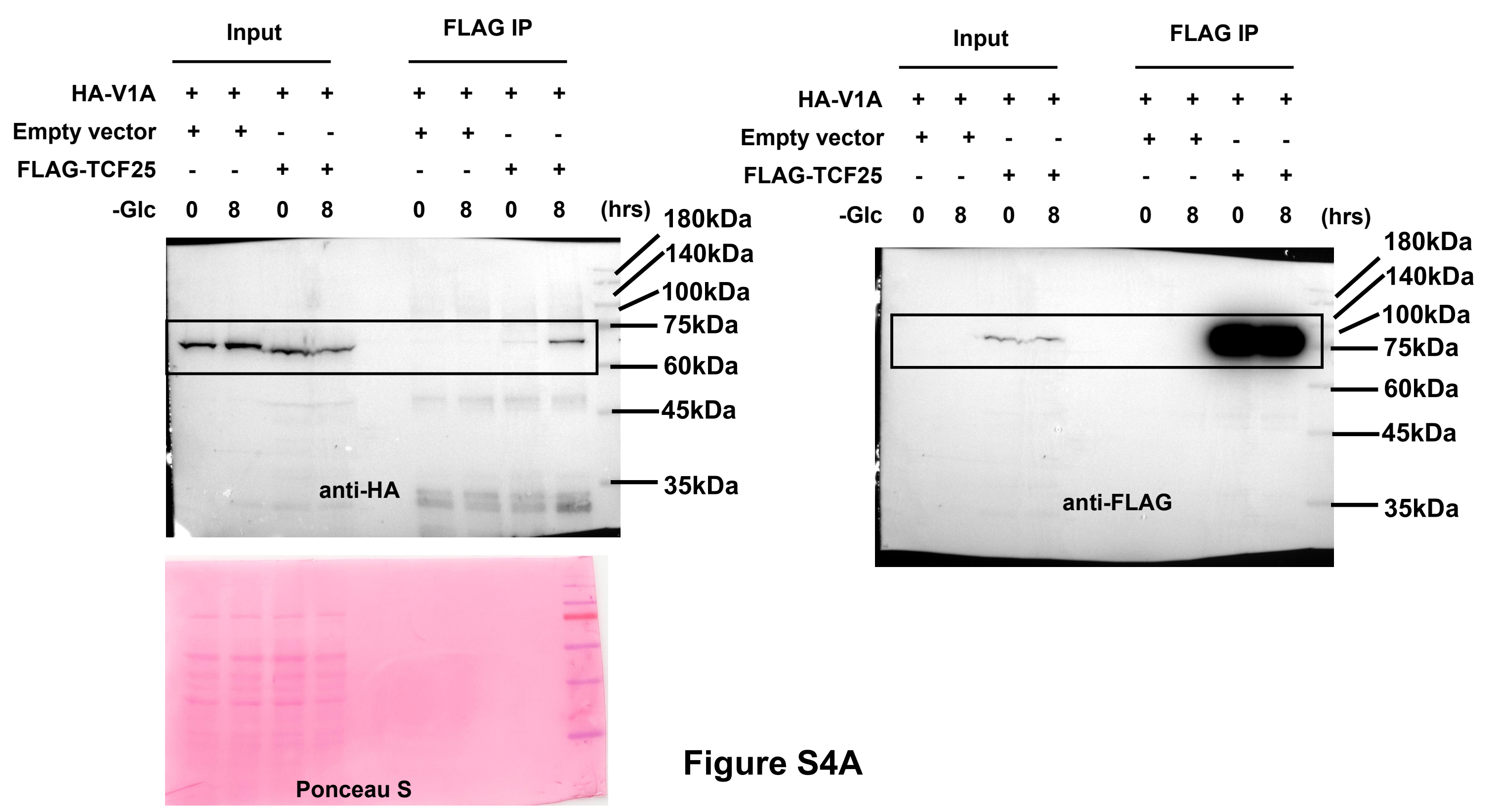

Figure S4A

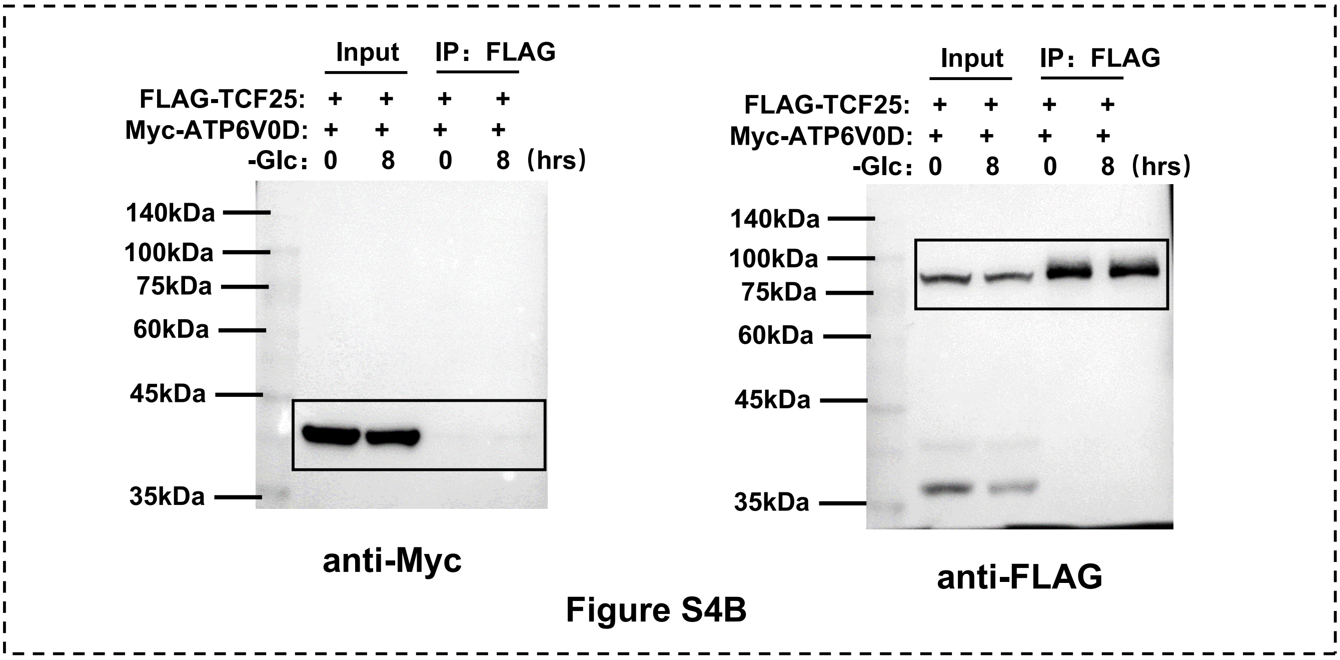

Figure S4B

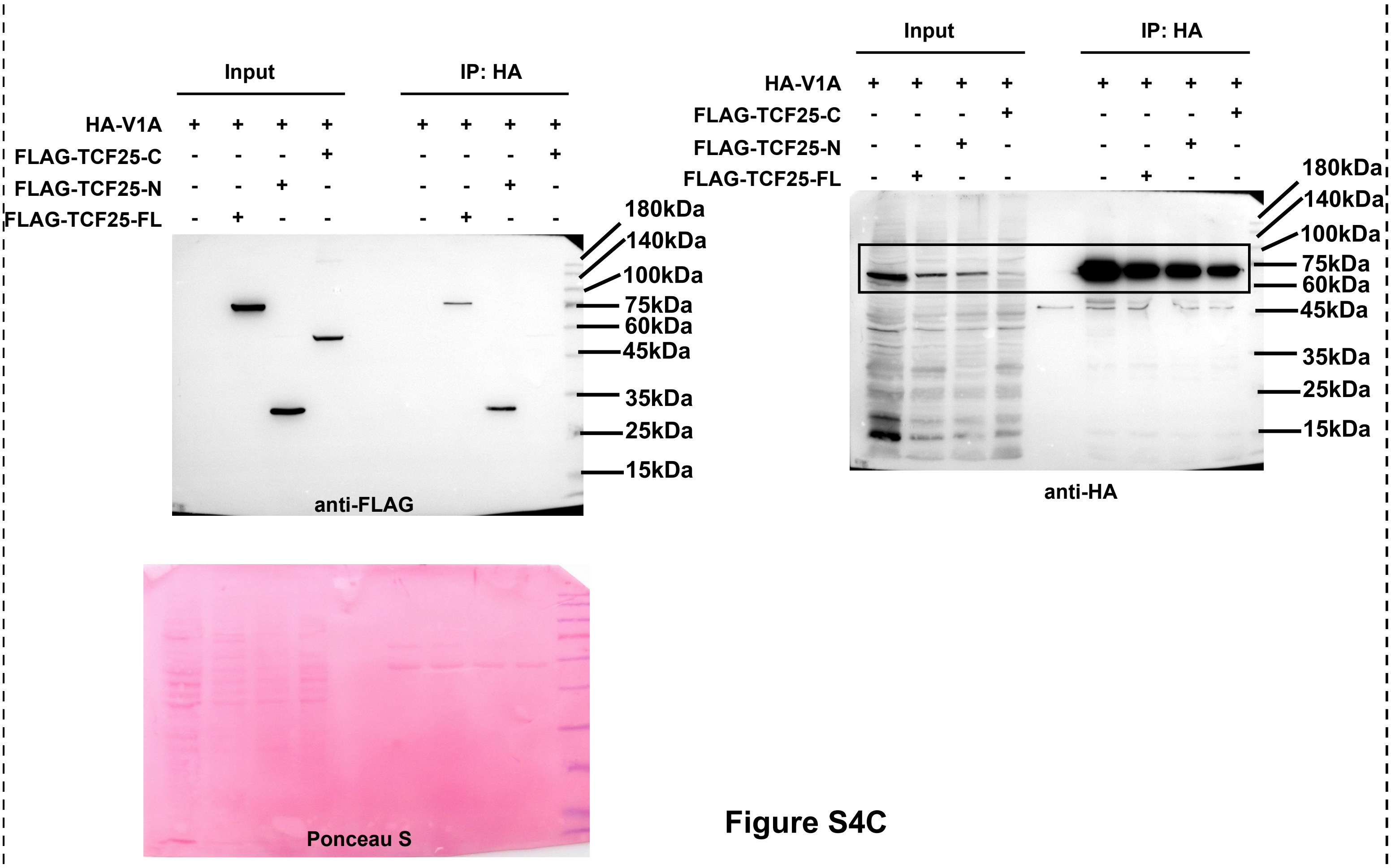

Figure S4C

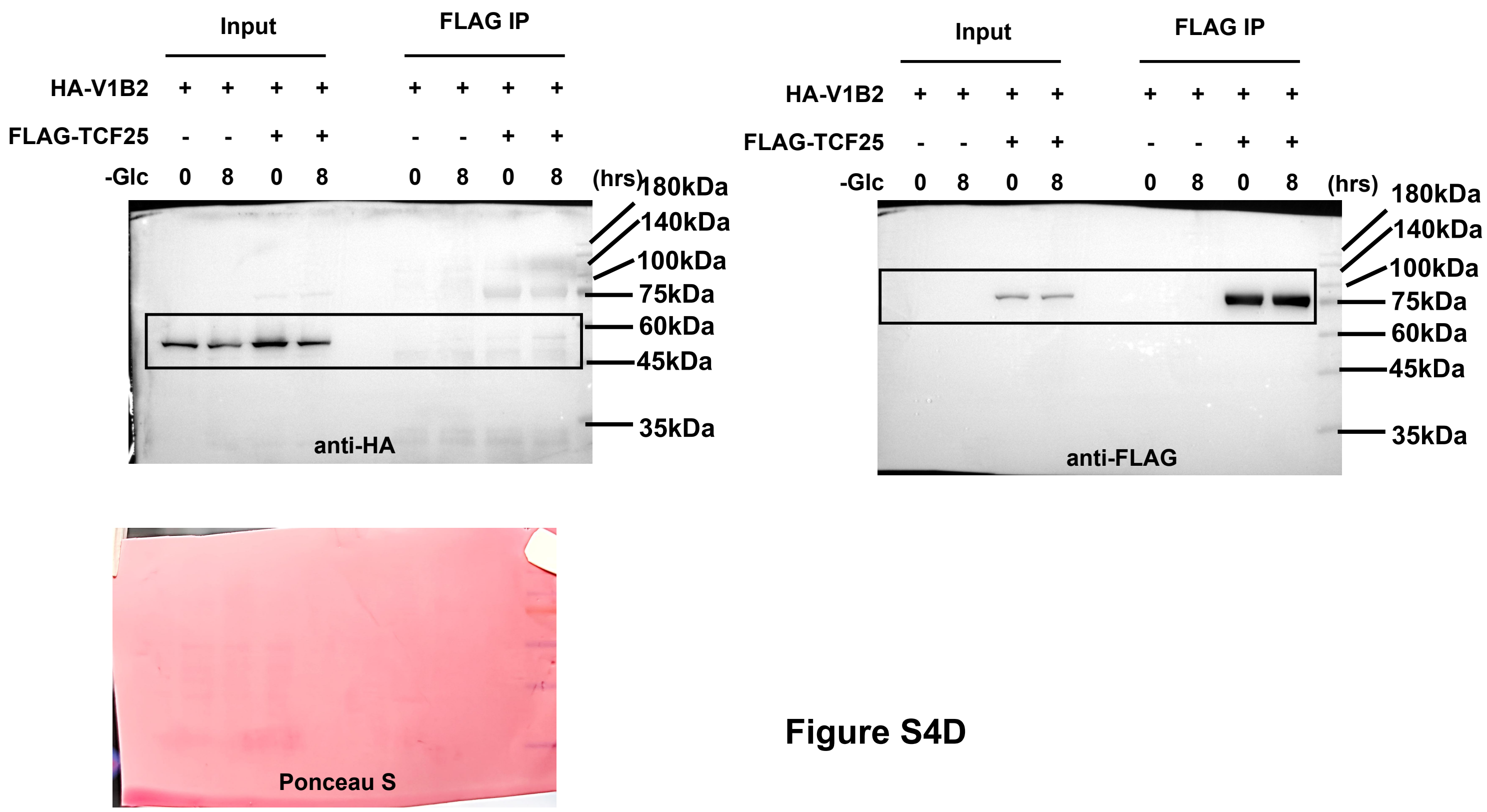

Figure S4D

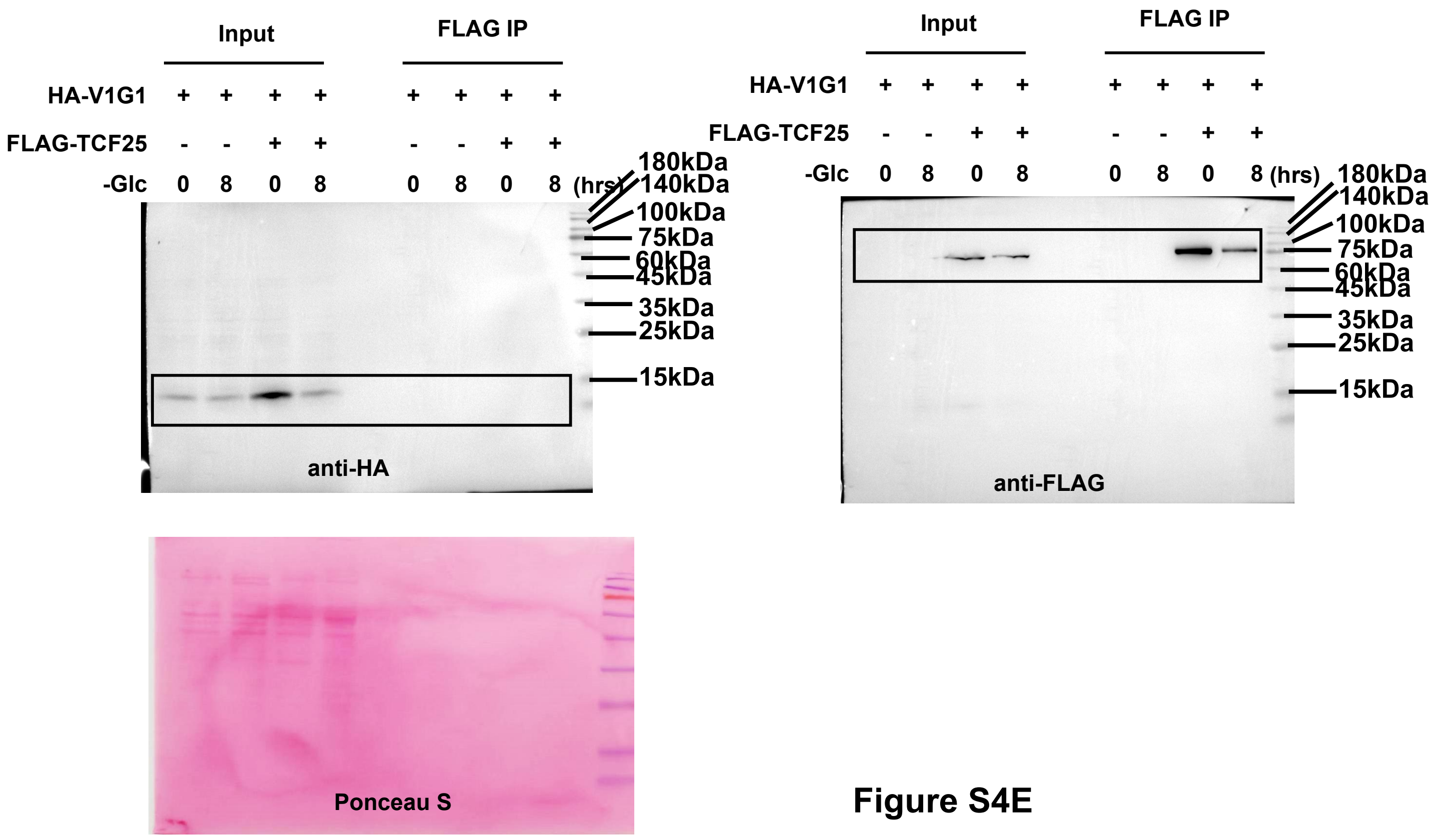

Figure S4E

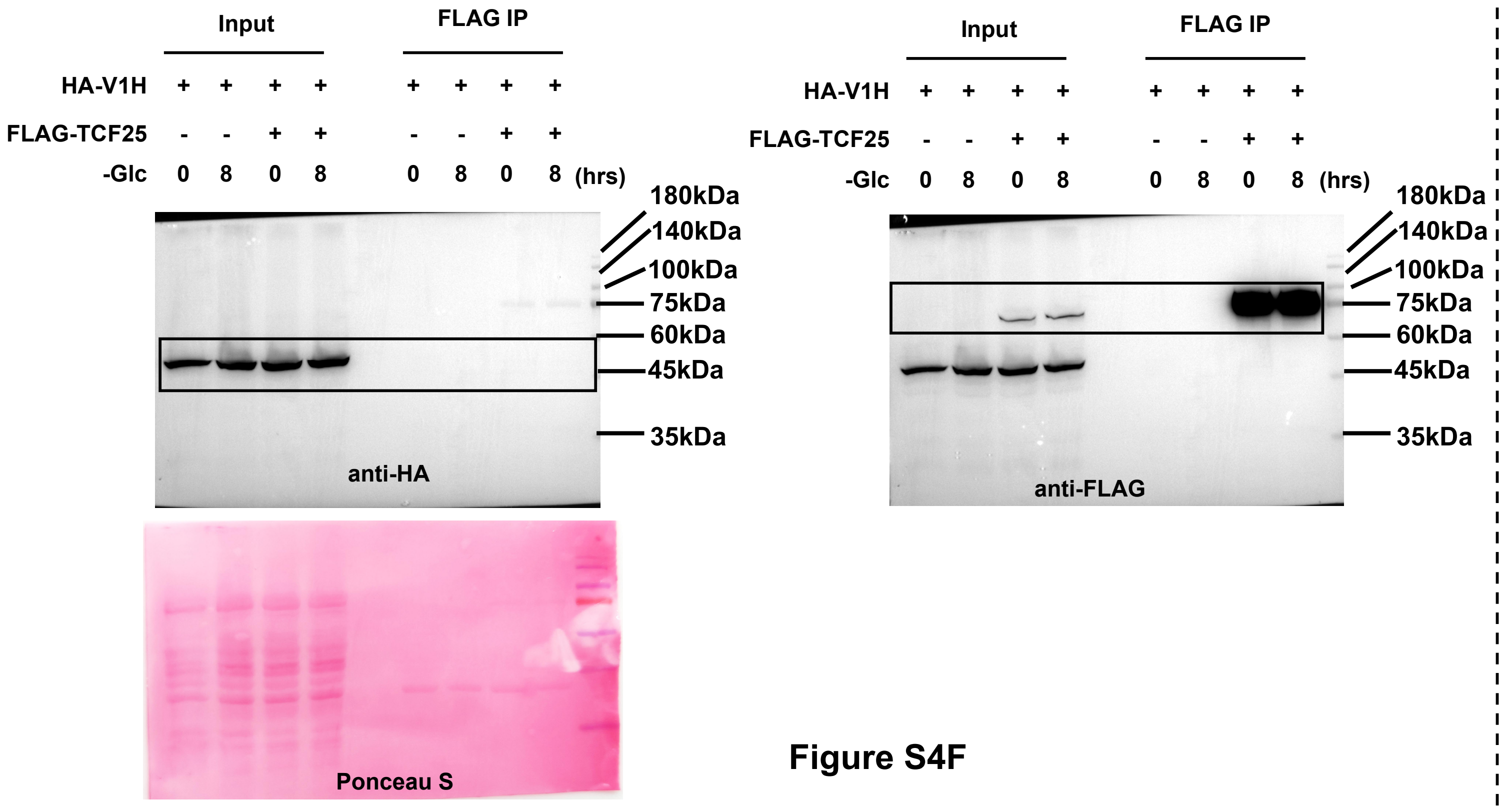

Figure S4F

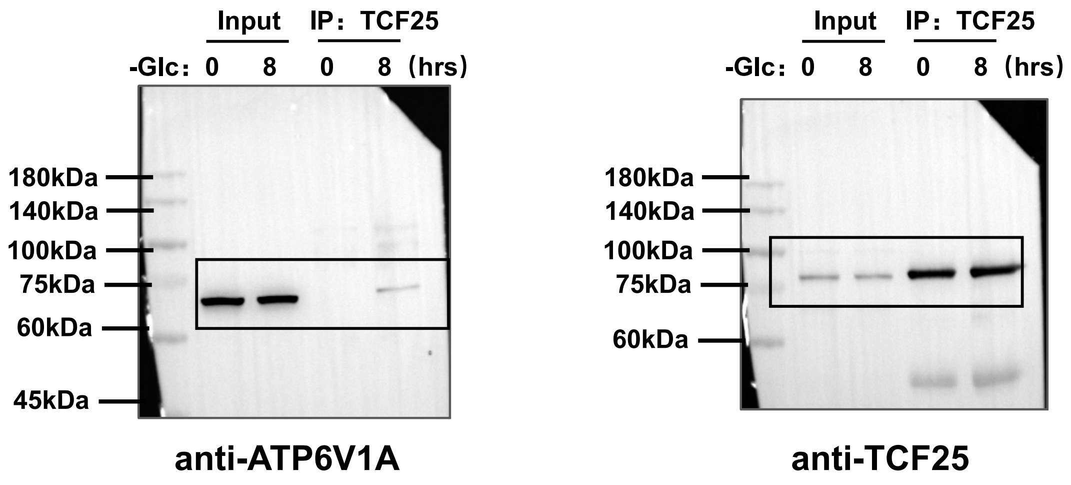

Figure S4G

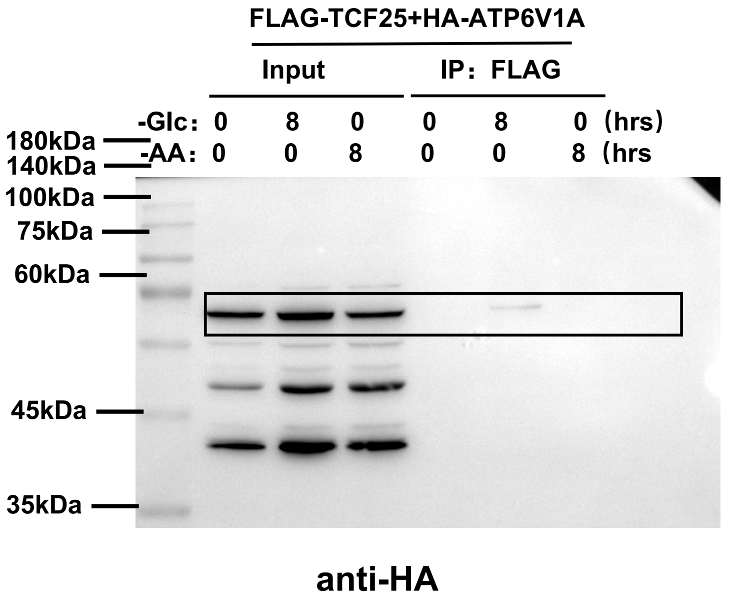

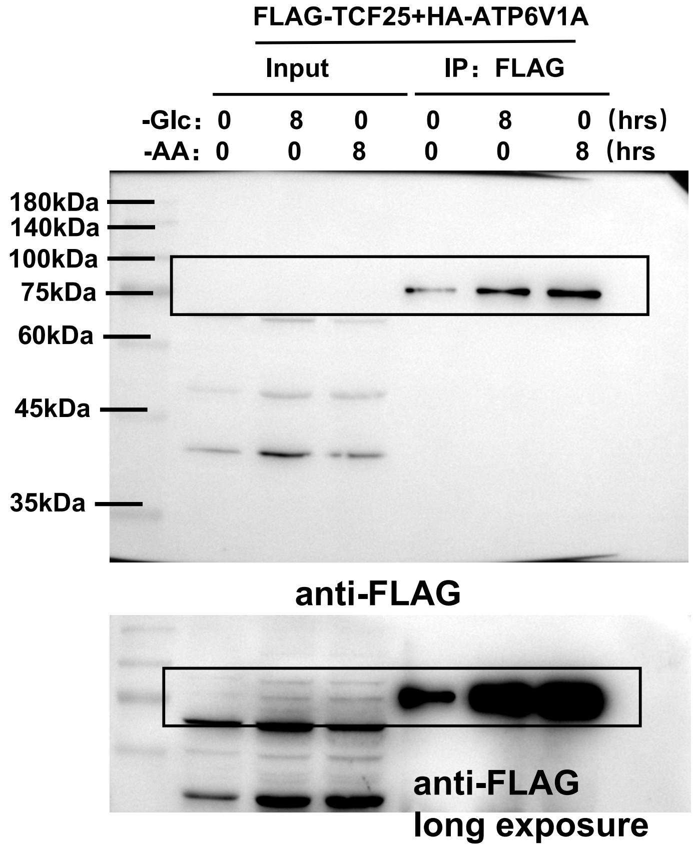

Figure S4H

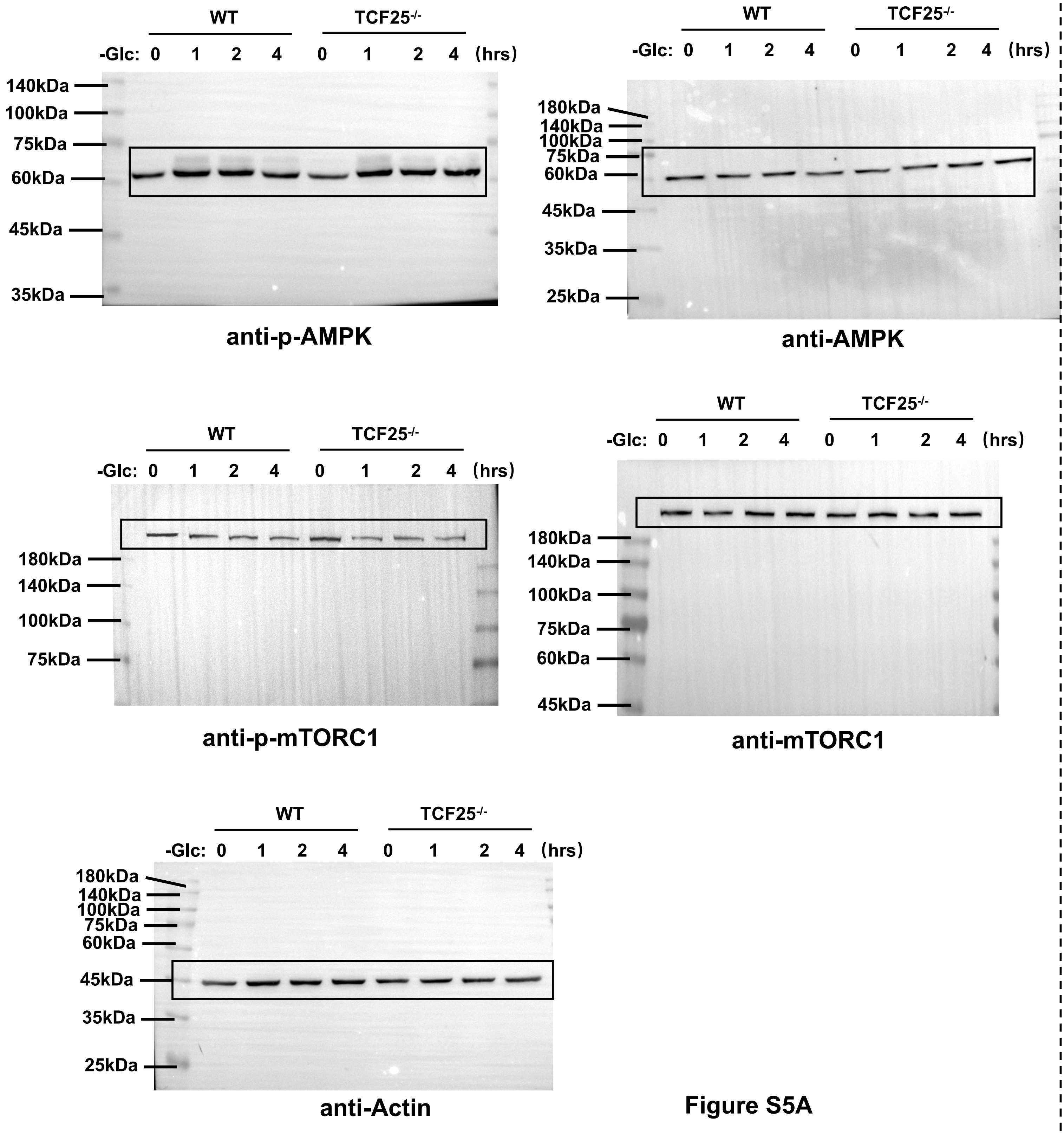
WT
TCF25-/-
-Glc: 0 1 2 4 0 1 2 4 (hrs)
140kDa
100kDa
75kDa
60kDa
45kDa
35kDa
anti-p-AMPK
WT
TCF25-/-
-Glc: 0 1 2 4 0 1 2 4 (hrs)
180kDa
140kDa
100kDa
75kDa
60kDa
45kDa
35kDa
25kDa
anti-AMPK
WT
TCF25-/-
-Glc: 0 1 2 4 0 1 2 4 (hrs)
180kDa
140kDa
100kDa
75kDa
anti-p-mTORC1
WT
TCF25-/-
-Glc: 0 1 2 4 0 1 2 4 (hrs)
180kDa
140kDa
100kDa
75kDa
60kDa
45kDa
anti-mTORC1
WT
TCF25-/-
-Glc: 0 1 2 4 0 1 2 4 (hrs)
180kDa
140kDa
100kDa
75kDa
60kDa
45kDa
35kDa
25kDa
anti-Actin

Figure S5A

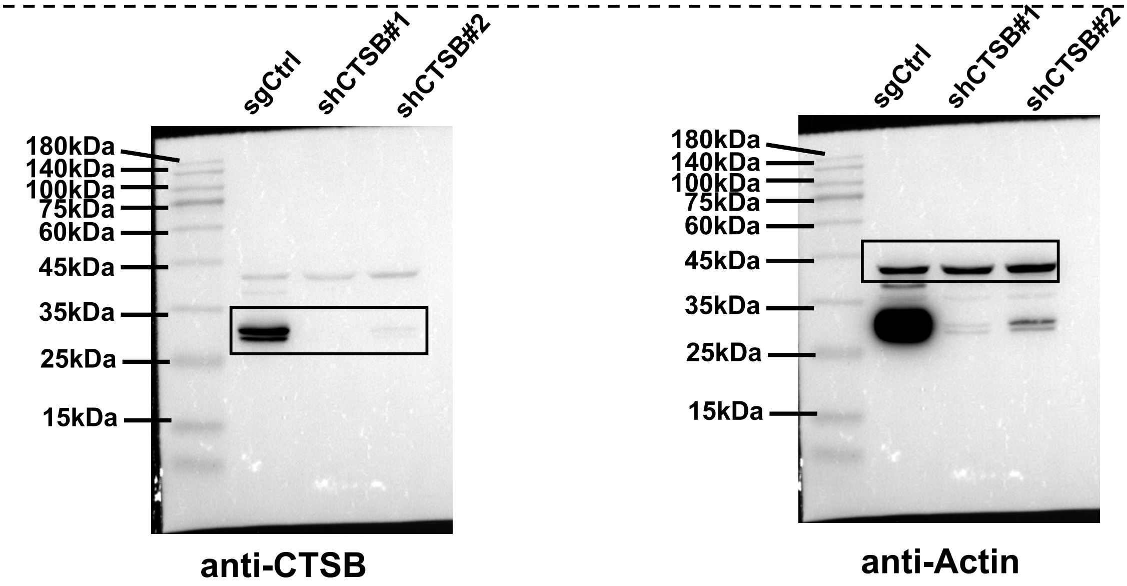

Figure S6B

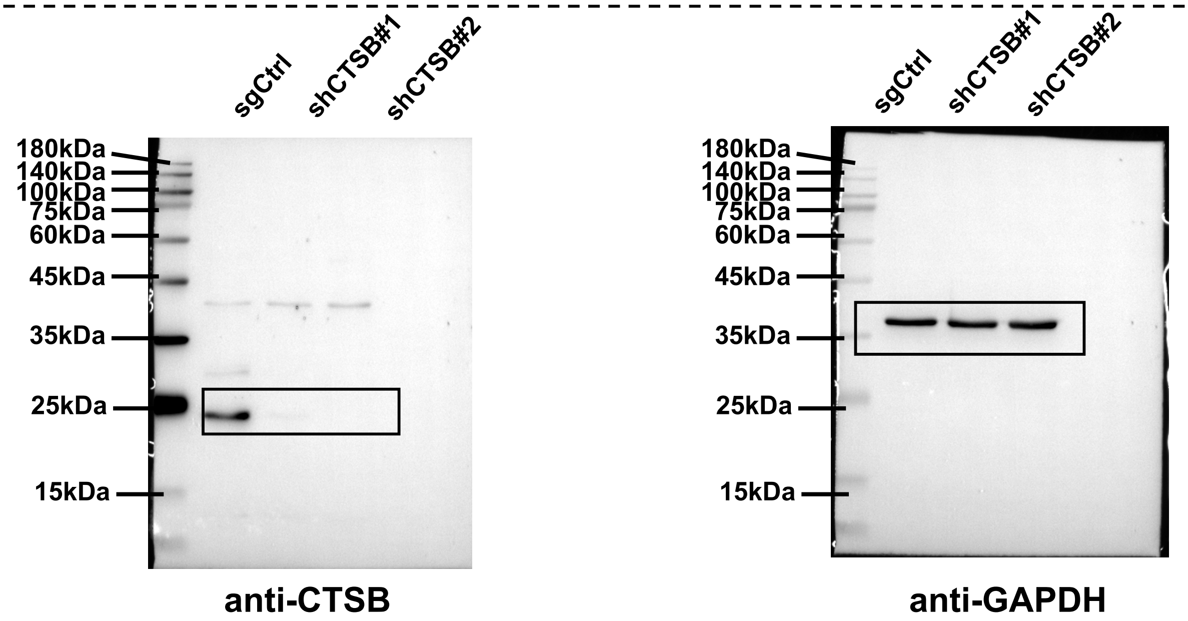

Figure S6D

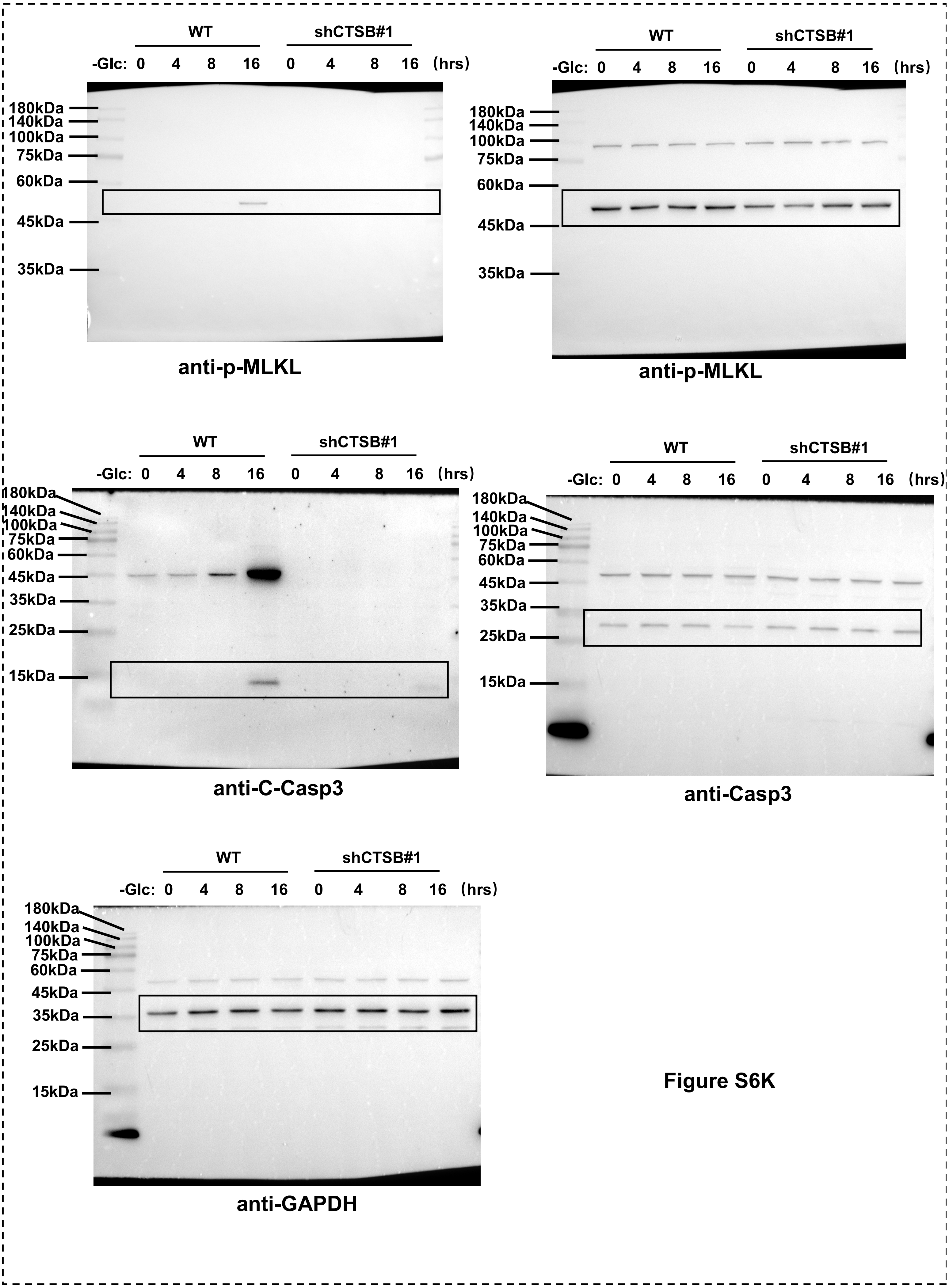
WT
shCTSB#1
-Glc: 0 4 8 16 0 4 8 16 (hrs)
180kDa
140kDa
100kDa
75kDa
60kDa
45kDa
35kDa
25kDa
15kDa
anti-p-MLKL
anti-p-MLKL
anti-C-Casp3
anti-Casp3
anti-GAPDH

Figure S6K

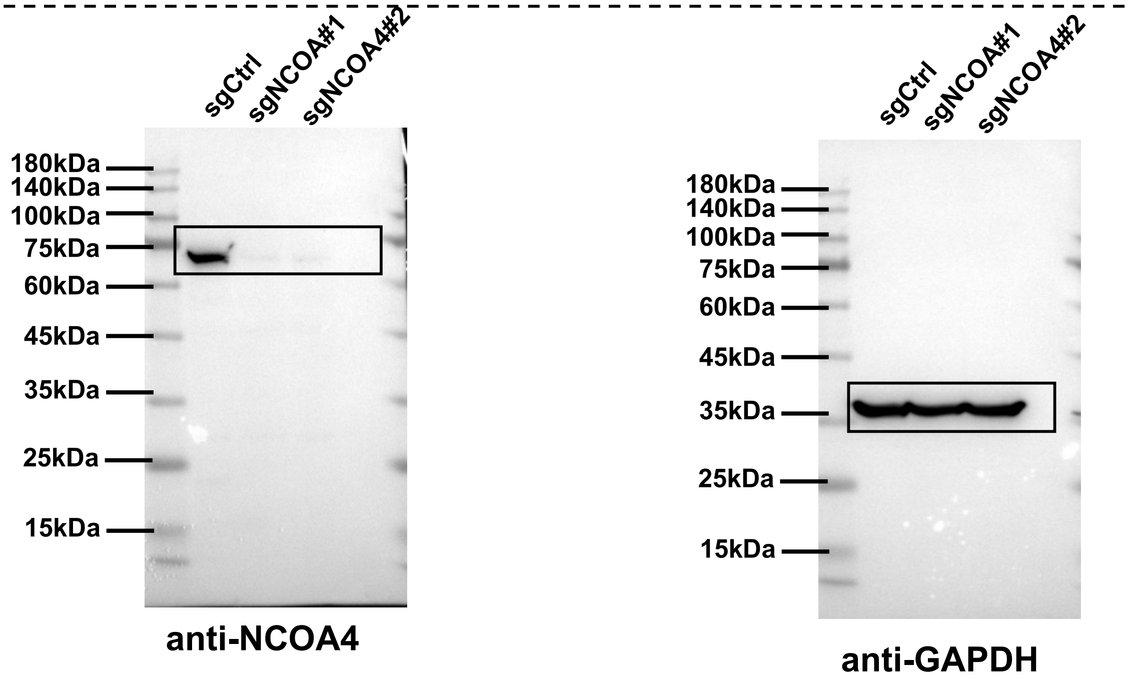

Figure S7B

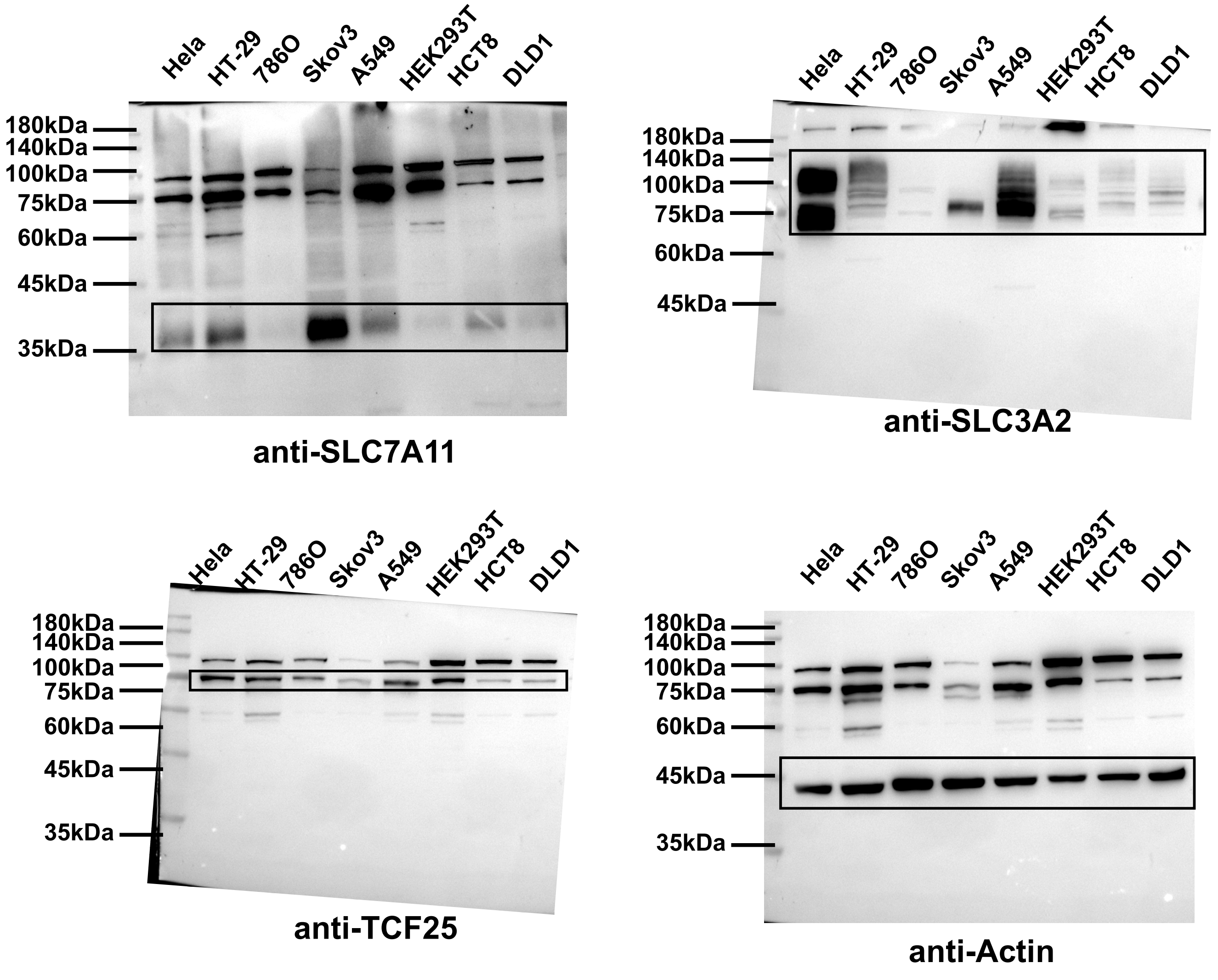

Figure S8A

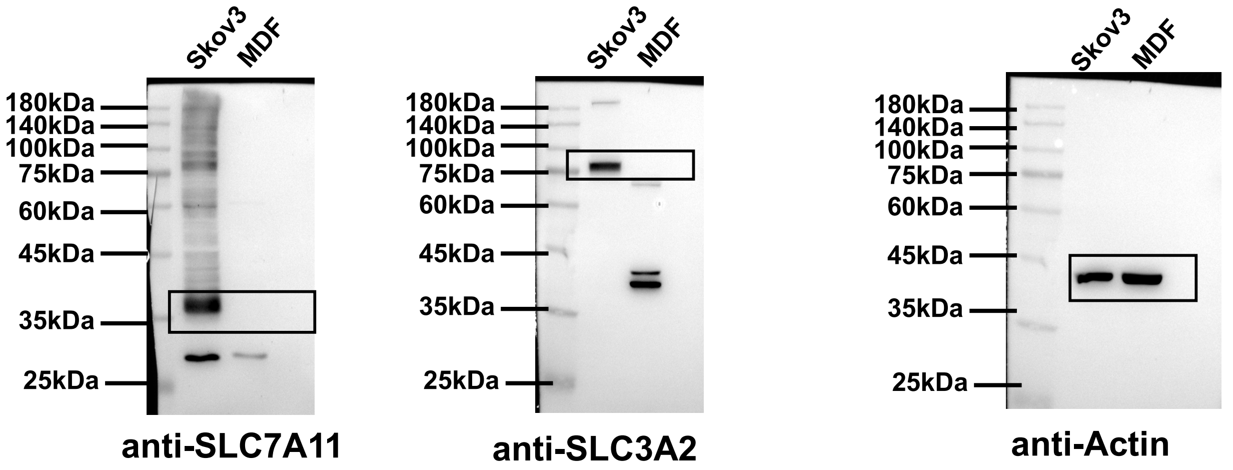

Figure S8A

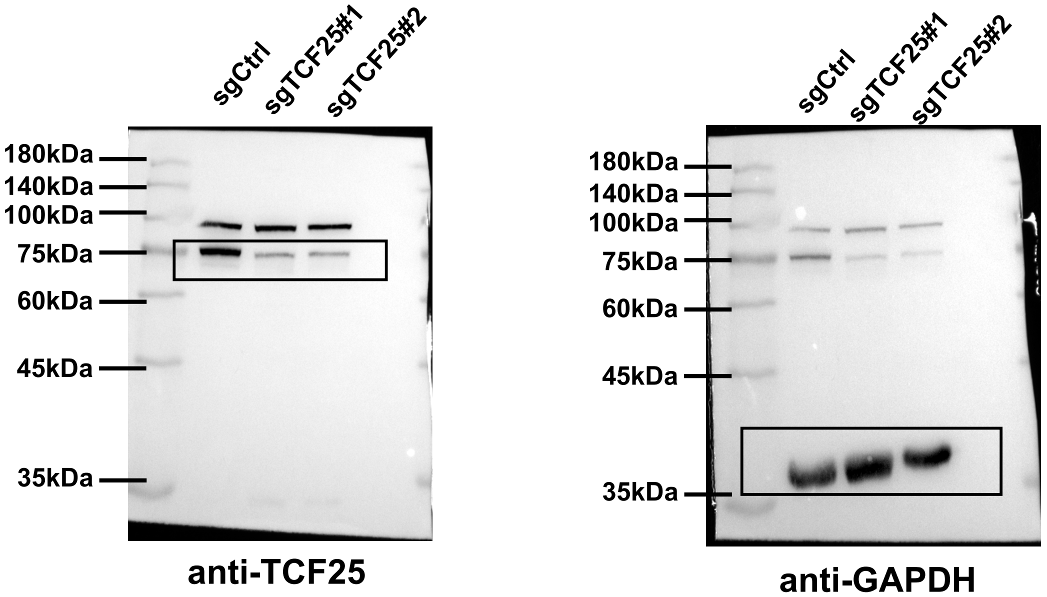

Figure S8B

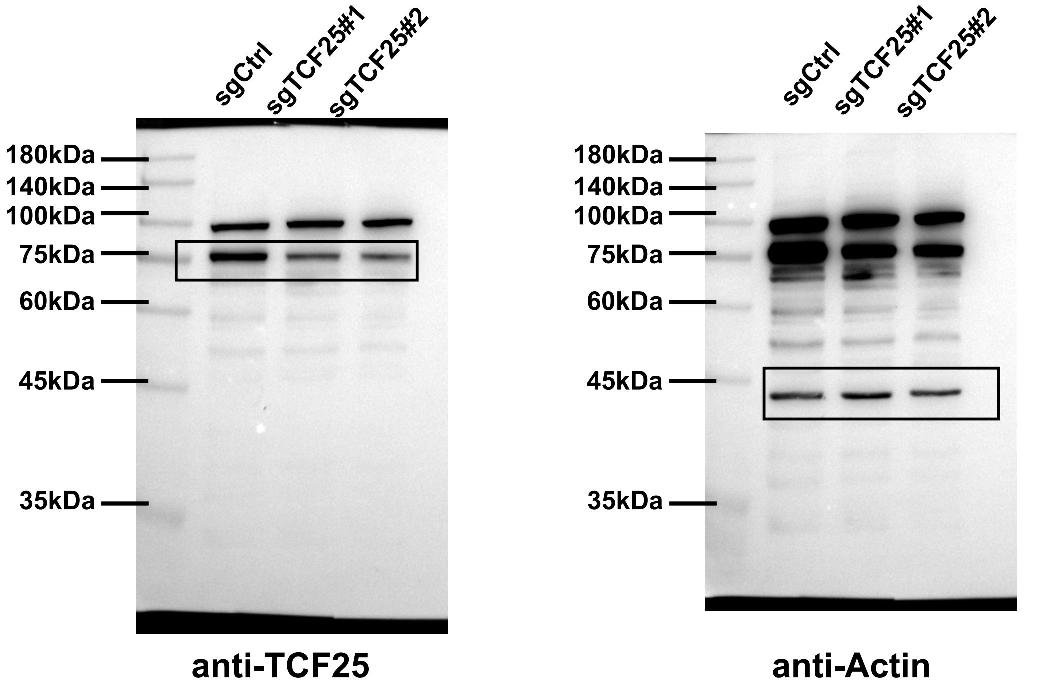

Figure S8D

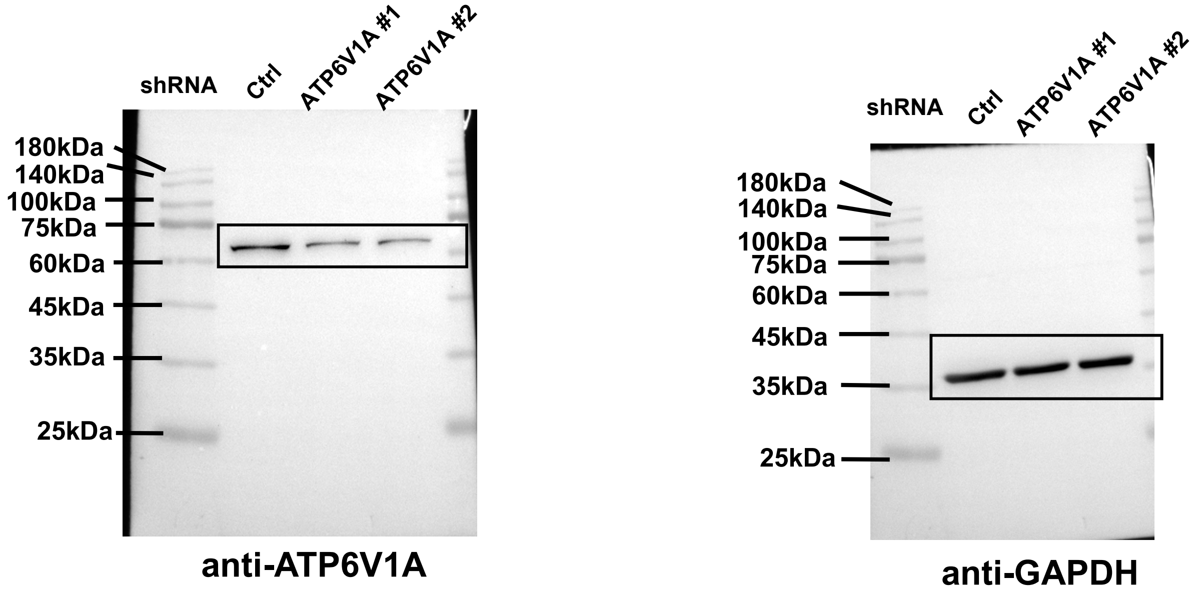

Figure S9A

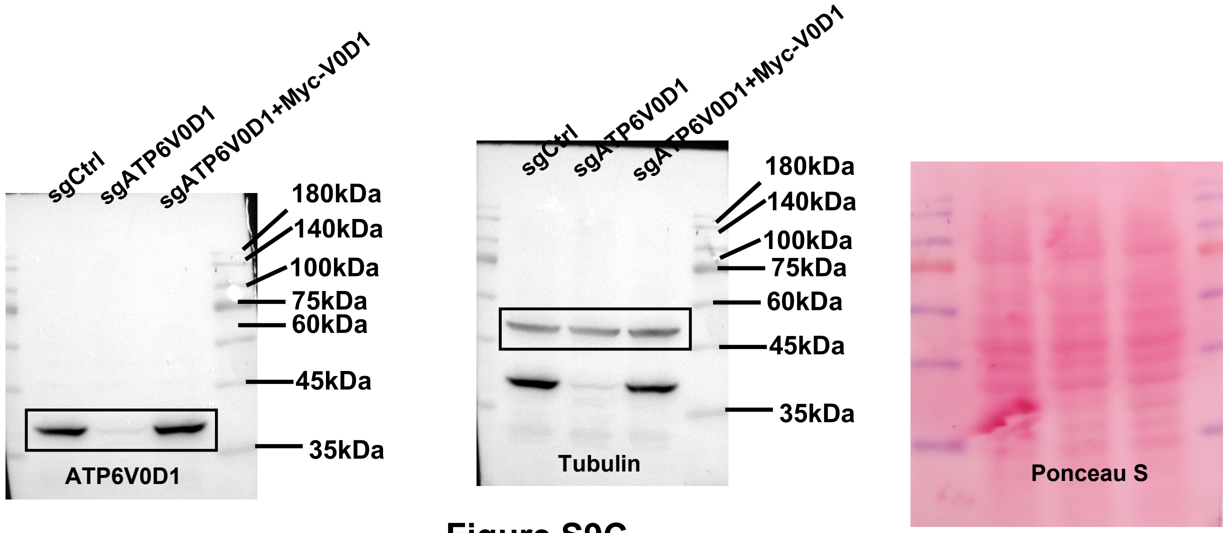

Figure S9C

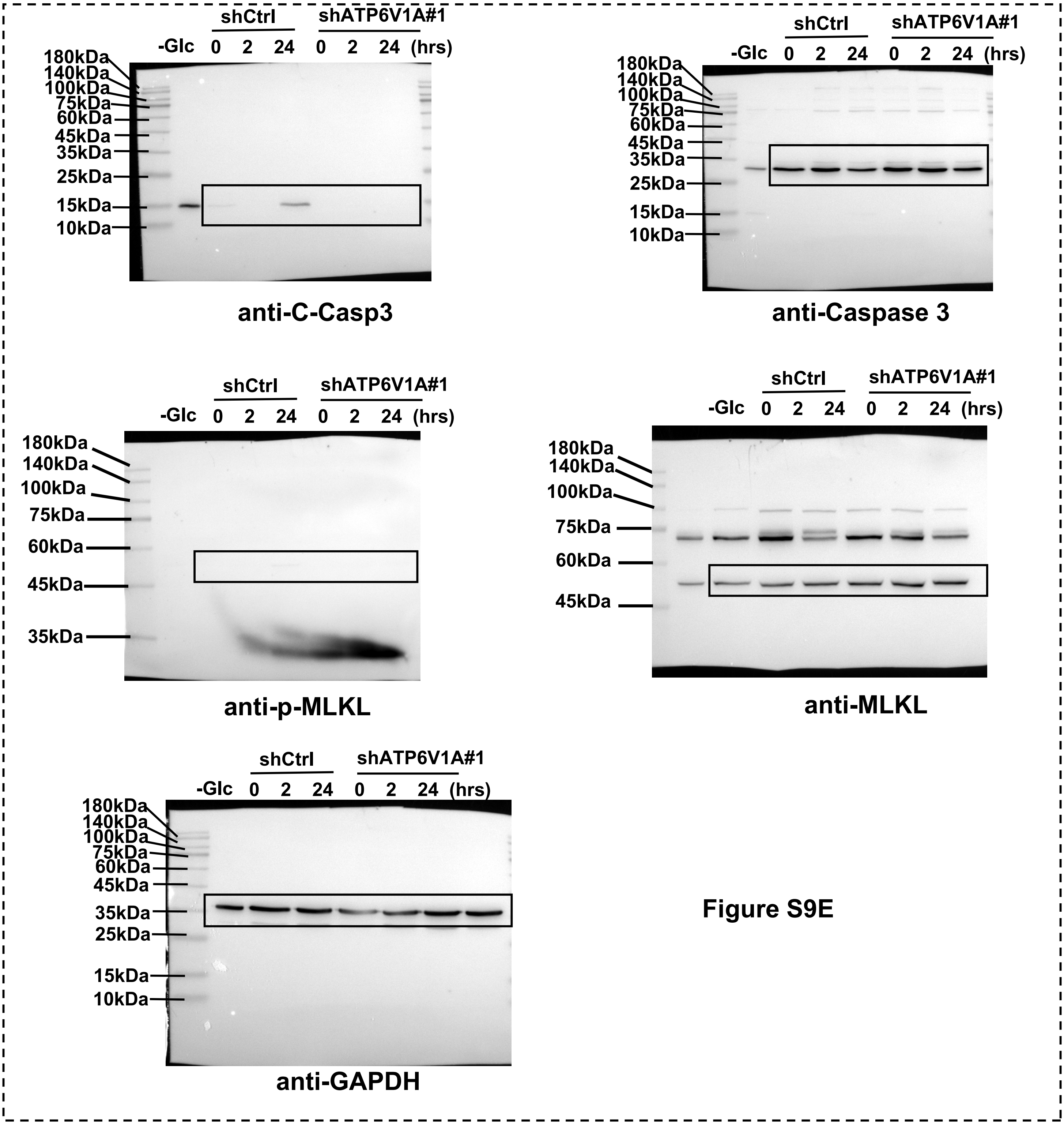
shCtrl
shATP6V1A#1
-Glc 0 2 24 0 2 24 (hrs)
180kDa
140kDa
100kDa
75kDa
60kDa
45kDa
35kDa
25kDa
15kDa
10kDa
anti-C-Casp3
anti-Caspase 3
anti-p-MLKL
anti-MLKL
anti-GAPDH

Figure S9E

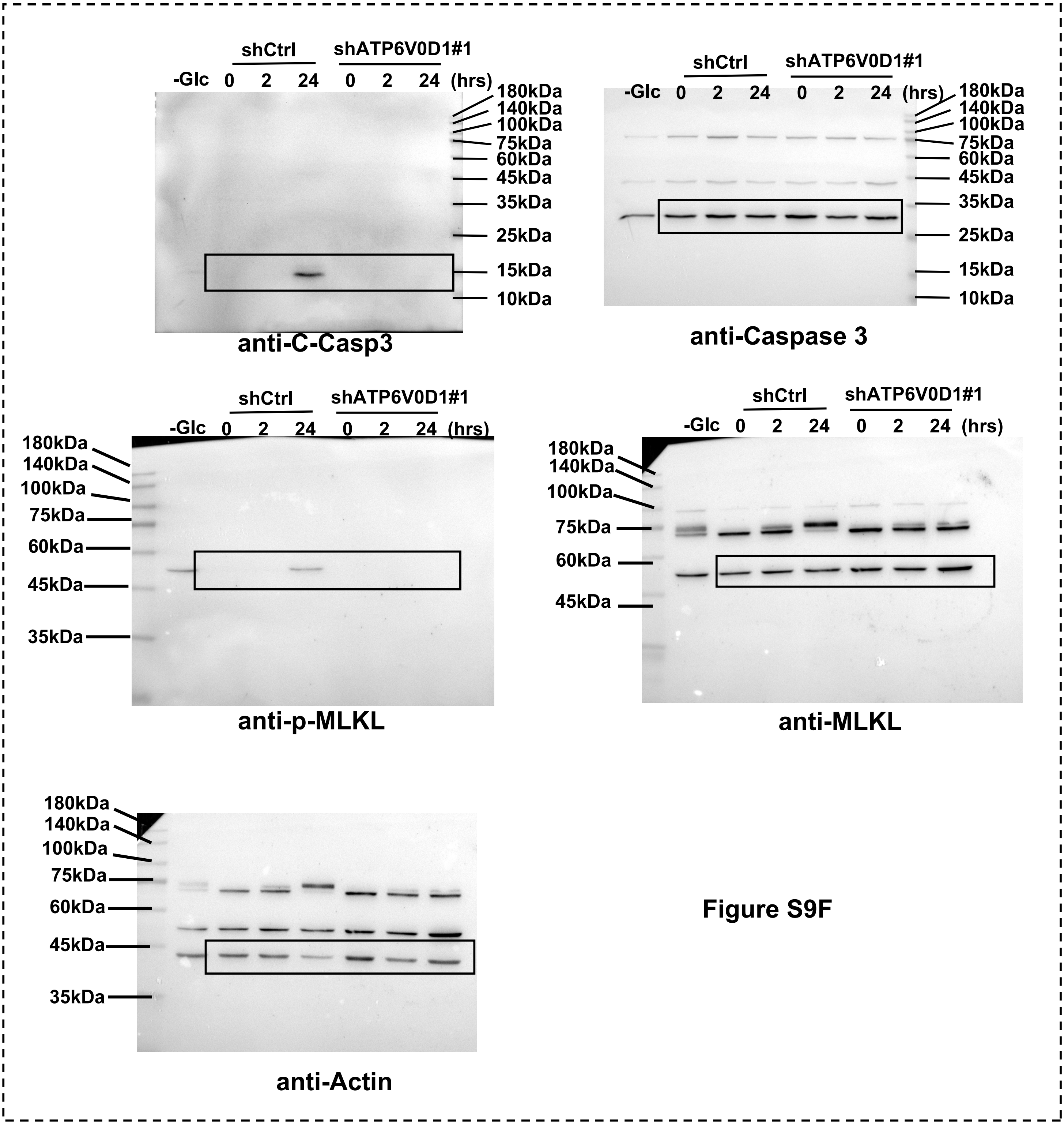
shCtrl
shATP6V0D1#1
-Glc 0 2 24 0 2 24 (hrs)
180kDa
140kDa
100kDa
75kDa
60kDa
45kDa
35kDa
25kDa
15kDa
10kDa
anti-C-Casp3
anti-Caspase 3
anti-p-MLKL
anti-MLKL
anti-Actin

Figure S9F
